# Bayesian and frequentist analysis of an Austrian genome-wide association study of colorectal cancer and advanced adenomas

**DOI:** 10.18632/oncotarget.21697

**Published:** 2017-10-09

**Authors:** Philipp Hofer, Michael Hagmann, Stefanie Brezina, Erich Dolejsi, Karl Mach, Gernot Leeb, Andreas Baierl, Stephan Buch, Hedwig Sutterlüty-Fall, Judith Karner-Hanusch, Michael M. Bergmann, Thomas Bachleitner-Hofmann, Anton Stift, Armin Gerger, Katharina Rötzer, Josef Karner, Stefan Stättner, Melanie Waldenberger, Thomas Meitinger, Konstantin Strauch, Jakob Linseisen, Christian Gieger, Florian Frommlet, Andrea Gsur

**Affiliations:** ^1^ Institute of Cancer Research, Medical University of Vienna, Vienna, Austria; ^2^ Center for Medical Statistics, Informatics, and Intelligent Systems, Section for Medical Statistics, Medical University of Vienna, Vienna, Austria; ^3^ Hospital Oberpullendorf, Oberpullendorf, Austria; ^4^ Department of Statistics and Operations Research, University of Vienna, Vienna, Austria; ^5^ University Hospital Dresden, Dresden, Germany; ^6^ Department of Surgery, Medical University of Vienna, Vienna, Austria; ^7^ Division of Oncology, Medical University of Graz, Graz, Austria; ^8^ Sozialmedizinisches Zentrum Süd, Vienna, Austria; ^9^ Helmholtz Center Munich, German Research Center for Environmental Health, Neuherberg, Germany; ^10^ Chair of Genetic Epidemiology, IBE, Faculty of Medicine, LMU Munich, Munich, Germany

**Keywords:** advanced colorectal adenomas, colorectal cancer, GWAS, model selection, MOSGWA

## Abstract

Most genome-wide association studies (GWAS) were analyzed using single marker tests in combination with stringent correction procedures for multiple testing. Thus, a substantial proportion of associated single nucleotide polymorphisms (SNPs) remained undetected and may account for missing heritability in complex traits. Model selection procedures present a powerful alternative to identify associated SNPs in high-dimensional settings. In this GWAS including 1060 colorectal cancer cases, 689 cases of advanced colorectal adenomas and 4367 controls we pursued a dual approach to investigate genome-wide associations with disease risk applying both, single marker analysis and model selection based on the modified Bayesian information criterion, mBIC2, implemented in the software package MOSGWA. For different case-control comparisons, we report models including between 1-14 candidate SNPs. A genome-wide significant association of rs17659990 (P=5.43×10^-9^, *DOCK3*, chromosome 3p21.2) with colorectal cancer risk was observed. Furthermore, 56 SNPs known to influence susceptibility to colorectal cancer and advanced adenoma were tested in a hypothesis-driven approach and several of them were found to be relevant in our Austrian cohort. After correction for multiple testing (α=8.9×10^-4^), the most significant associations were observed for SNPs rs10505477 (P=6.08×10^-4^) and rs6983267 (P=7.35×10^-4^) of *CASC8*, rs3802842 (P=8.98×10^-5^, *COLCA1,2*), and rs12953717 (P=4.64×10^-4^, *SMAD7*). All previously unreported SNPs demand replication in additional samples. Reanalysis of existing GWAS datasets using model selection as tool to detect SNPs associated with a complex trait may present a promising resource to identify further genetic risk variants not only for colorectal cancer.

## INTRODUCTION

Numerous genome-wide association studies (GWAS) in diverse complex diseases have uncovered hundreds of genetic risk factors by determining hundred thousands of single nucleotide polymorphisms (SNPs) in cohorts of thousands of individuals in a hypothesis-free approach. Although these findings provide valuable insights into the genetic architecture of common diseases they collectively account for a relatively small proportion of heritability [1].

Colorectal carcinogenesis is a complex multi-step process influenced by both, genetic and environmental risk factors. Only 5-10% [2] of all colorectal cancer (CRC) cases can be ascribed to hereditary syndromes and explained by rare but high-penetrant germline mutations. Another 30% of CRCs can be attributed to non-syndromic familial cases with increased familial risk but without evidence of predisposing mutations. The remaining CRCs evolve sporadically and are influenced by numerous genetic variants with low penetrance but of high prevalence in the population (>1%). This common disease-common variant hypothesis was formulated in the early days of GWAS, but was relativized when identified risk loci explained only a small fraction of genetic variance in complex traits. More refined concepts include the common disease-rare variant hypothesis [2], the infinitesimal and the broad sense heritability model (discussed in [3]).

GWAS of CRC conducted in European but also Asian populations have discovered so far more than 50 risk variants [4–29] mapping to 23 susceptibility loci. Although GWAS have successfully identified multiple associations of genetic variants with risk of CRC, collectively the CRC SNPs identified in European populations account only for 8% of familial CRC risk [30]. Additional rare risk variants still remain undetected and in part may account for the missing heritability of CRC.

Typically, GWAS aims at the identification of a relatively small set of SNPs associated with the investigated phenotype. SNPs exceeding a genome-wide significance threshold (P < 5×10^-8^) are tested for replication in independent samples. Inevitably, these necessarily stringent penalties for multiple testing have the consequence that a relatively large proportion of associated SNPs cannot be detected. Consequently, the majority of missing heritability may be due to SNPs with effects below the level of genome-wide significant associations [3].

The vast majority of GWAS have been analyzed via single marker analysis. One advantage of this approach is its computational inexpensiveness. However, this standard approach to analyze association with disease risk for each SNP individually assumes complete independence of the analyzed SNPs [31]. In contrast, genetic risk often can be explained as the influence of multiple SNPs mapping to various chromosomal regions resulting in a phenotype [32]. Furthermore, single marker tests cannot take into consideration the distinct correlation structure among SNPs caused by linkage disequilibrium (LD) and interaction effects [31]. Usually, individual effect sizes of SNPs are small, but collectively their impact on the phenotype can be substantial [32]. There are other weighty reasons for considering all genotyped SNPs simultaneously in analysis of GWAS. The predictive power of a single SNP is usually very low, but considering more disease relevant SNPs can improve the accuracy of prediction [33]. In the context of complex diseases multiple genes are involved in disease etiology, thus a joint analysis of multiple SNPs can be more informative and better reflect the relationship between genotype and phenotype than single SNP models [34].

A comprehensive overview of the advantages of model selection based approaches to analysis of GWAS is provided in Frommlet et al. 2016 [35], particularly addressing selection procedures based on modifications of the Bayesian information criterion (BIC) [36]. In high dimensional settings like GWAS where only a small number of SNPs is expected to be associated with disease (under sparsity), it has been shown repeatedly that BIC tends to select too large models. Various modifications of BIC have been proposed to solve this problem, among them mBIC2 [37, 38] which was designed to control the false discovery rate (FDR).

Here, we pursued a dual analysis strategy, reporting results from both single marker tests and MOSGWA [39], an implementation of a model selection procedure based on mBIC2. Genome-wide SNP data of 1060 CRC cases, 689 patients with advanced colorectal adenomas and 4367 controls were analyzed presenting the first GWAS of CRC in an Austrian population.

## RESULTS

Downstream analysis was performed for 492,217 SNPs using the software package MOSGWA. Additionally, results from single marker analysis via PLINK are reported using Cochran Armitage trend test (CAT) as well as univariate logistic regression models including the first four principle components as covariates to account for population structure.

Our study population consisted of four different case and control groups, CRC cases (A), advanced adenomas (B), colonoscopy-negative CORSA controls (C) and KORA controls (D) (Table 1). Further clinical characteristics of CRC cases and advanced adenomas are provided in Supplementary Table 1. Specifically, we report the following four case-control comparisons: A vs. C, A vs. CD, AB vs. CD and B vs CD (Table 2).

**Table 1:** Study population

	Total_Pre-QC_	Total_Post-QC_ (%)	Male (%)	Female (%)	Mean age ± SD [y]
**CRC (A)**	1060	978 (100.0)	584 (59.7)	394 (40.3)	63.5 ± 12.0
**AA (B)**	689	636 (100.0)	428 (67.3)	208 (32.7)	64.5 ± 10.3
**Control_CORSA_ (C)**	928	855 (100.0)	496 (58.0)	359 (42.0)	65.1 ± 11.8
**Control_KORA_ (D)**	3439	3439 (100.0)	1690 (49.1)	1749 (50.9)	53.8 ± 14.0
**Total**	6116	5908	3198	2710	58.2 ± 13.9

CRC Colorectal cancer cases.

AA Advanced adenomas.

**Table 2:** Single marker tests and model selection

SNP	Chromosome	Gene	OR (Logistic)	P (Logistic)	Rank (Logistic)	OR (Model)	P (SM)
**A vs. C (978 cases vs. 855 controls)**							
rs1912804	16q23.1	*WWOX*	1.69	3.39E-07	1	1.70	1.96E-07
rs9583269	13q33.3	*MYO16*	0.69	6.19E-07	2	0.69	1.24E-06
rs10495672	2p24.2	*KCNS3*	1.43	3.23E-06	7	1.46	1.95E-06
**A vs. CD (978 cases vs. 4294 controls)**							
rs17659990	3p21.2	*DOCK3*	1.93	1.35E-07	1	1.98	1.59E-08
rs694339	18q22.3	*CBLN2*	1.97	1.41E-07	2	1.99	7.89E-07
rs12916300	15q13.1	*HERC2*	1.35	3.75E-07	4	1.35	1.37E-04
rs16845107	3q13.2	*WDR52*	0.52	5.94E-07	6	0.52	1.49E-06
rs11927424	3p11.1	*C3orf38*	1.31	1.17E-06	11	1.32	1.07E-07
rs16869961	4p15.31	*KCNIP4*	0.75	1.51E-06	13	0.74	1.25E-04
rs7774435	6p21.32	*HLA-DQA2*	-	-	-	1.46	3.52E-04
**AB vs. CD (1614 cases vs. 4294 controls)**							
rs17659990	3p21.2	*DOCK3*	1.88	**5.43E-09**	1	1.96	2.94E-10
rs7742915	6p21.2	*BTBD9*	1.31	8.52E-08	2	1.32	1.44E-06
rs16944613	15q26.1	*CRTC3*	1.32	1.49E-07	4	1.31	3.71E-07
rs13129679	4p16.3	*RNF4*	2.30	2.38E-07	5	2.42	8.82E-07
rs12953717	18q21.1	*SMAD7*	1.26	3.00E-07	6	1.27	6.68E-08
rs742223	6p24.1	*TMEM170B*	0.60	5.47E-07	9	0.59	9.49E-08
rs2184857	1q43	*CHRM3*	0.79	7.10E-07	11	0.78	3.35E-09
rs4954585	2q22.1	*CXCR4*	1.26	9.84E-07	12	1.26	2.29E-08
rs7942260	11q21	*PIWIL4*	0.69	7.28E-06	30	0.67	1.84E-06
rs7221059	17q25.2	*LINC00338*	0.76	1.02E-05	45	0.74	2.92E-05
rs4361767	8p23.1	*LOC157273*	0.77	1.31E-05	62	0.75	6.91E-05
rs340145	3q13.2	*TMPRSS7*	0.82	3.91E-05	142	0.79	1.45E-04
rs7774435	6p21.32	*HLA-DQA2*	-	-	-	1.65	5.82E-05
rs3130954	6p21.33	*HCG27*	-	-	-	1.79	1.04E-04
**B vs. CD (636 cases vs. 4294 controls)**							
rs7944251	11q14.3	*FAT3*	0.66	3.97E-07	2	0.66	1.27E-08

A  CRC cases (CORSA).

B  Advanced adenomas (CORSA).

C  Controls (CORSA).

D  Controls (KORA).

OR (Model) Odds ratio based on the coefficients of the model selected by MOSGWA.

P (SM) Single marker test P-value (Cochran Armitage trend test).

OR (Logistic) Odds ratio based on univariate logistic model.

P (Logistic) P-value of univariate logistic model.

Rank (Logistic) Rank of the SNP in the top SNP list of P (Logistic) sorted by P-value.

-  HLA region excluded from logistic models.

Table 2 provides for each of the four comparisons some basic information and odds ratios for those SNPs corresponding to the model selected by MOSGWA. Additionally, P-values from CAT test, odds ratios, and P-values based on the univariate logistic model as well as the corresponding rank of each SNP according to the logistic model are presented. A list of the 200 top ranking SNPs for each contrast is provided as Supplementary Materials (Supplementary Table 2).

### A vs. C

For the comparison A vs. C, considering only Austrian cases and controls, MOSGWA selected a model of size three including SNPs rs1912804, rs9583269, and rs10495672. The best SNP rs1912804 has a marginal P-value of 3.39×10^-7^ that is not significant at the commonly adopted genome-wide significance level α=5.0×10^-8^. The three selected SNPs are among the top seven single marker SNPs (ranks 1, 2 and 7).

### A vs. CD

Adding KORA controls increased power to detect associated SNPs. Accordingly, the comparison A vs. CD yielded a model containing seven SNPs including the top SNP rs17659990 (P=1.35×10^-7^; *DOCK3*).

### AB vs. CD

For the joint analysis of CRC and advanced adenomas versus all controls, AB vs. CD, MOSGWA selected 14 SNPs, including rs17659990 (P=5.43×10^-9^, *DOCK3*) that reached the generally accepted level of genome-wide significance, followed by borderline significant rs7742915 (P=8.52×10^-8^, *BTBD9*), rs16944613 (P=1.49×10^-7^, *CRTC3*), rs13129679 (P=2.38×10^-7^, *RNF4*) and rs12953717 (P=3.00×10^-7^, *SMAD7*), a well-known CRC susceptibility variant.

### B vs. CD

For the comparison of advanced adenomas against the combined control (B vs. CD) MOSGWA identified one SNP on 11q14 (rs7944251, P=3.97×10^-7^, *FAT3*). Using only CORSA controls (B vs. C) there was not sufficient power to detect any SNP and MOSGWA selected the null model.

Genotype distributions of 56 CRC or colorectal adenoma susceptibility SNPs previously identified by GWAS were analyzed in the present genome-wide data set. Uncorrected P-values for all calculated case-control comparisons are provided in Table 3. For CRC SNPs, not covered by Axiom array, the distance of the proxy SNP from the original CRC SNP is provided in base pairs. P-values below 0.05 are given in bold, P-values below Bonferroni corrected significance level α=8.9×10^-4^ are given in bold and are underlined. Several SNPs previously identified by CRC GWAS exhibit significantly different genotype distributions in cases and controls. The strongest associations were found for rs12953717 of *SMAD7* on chromosome arm 18q21.1 (P_(Avs.C)_=4.64×10^-4^, P_(Avs.CD)_=2.83×10^-5^, P_(ABvs.CD)_=8.64×10^-6^). Significant associations were also observed for the *SMAD7* SNP rs4939827 (P_(Avs.CD)_=4.03×10^-4^, P_(ABvs.CD)_=1.53×10^-4^) and the *RHPN2* SNP rs10411210 on 19q13.11 (P_(Avs.CD)_=3.28×10^-4^). Several SNPs of the well-known CRC susceptibility loci on chromosome 8 showed differentially distributed genotypes, among them rs16892766 on 8q23.3 (P_(Avs.CD)_=5.48×10^-4^, *EIF3H*), rs10505477 on 8q24.21 (P_(Avs.C)_=6.08×10^-4^, *CASC8*), and rs6983267 also located on 8q24.21 (P_(Avs.C)_=7.35×10^-4^, *MYC*). Also rs3802842 on chromosome 11q23.1 showed significant associations with CRC risk across different comparisons (P_(Avs.C)_=8.98×10^-5^, P_(Avs.CD)_=8.62×10^-5^, P_(ABvs.CD)_=1.86×10^-5^, *COLCA1,2*).

**Table 3:** Associations of CRC susceptibility SNPs identified by preceding GWAS

SNP	Chr.	Gene	Ref.	Distance	P_AvC	P_AvCD	P_ABvC	P_ABvCD	P_BvC	P_BvCD
rs10911251	1q25.3	*LAMC1*	^23^	3492	2.03E-01	5.59E-01	2.26E-01	6.08E-01	3.38E-01	8.03E-01
rs6687758	1q41	*DUSP10*	^13^	3769	2.40E-01	5.32E-01	4.24E-01	2.57E-01	8.29E-01	3.59E-01
rs6691170	1q41	*DUSP10*	^13^	265	2.29E-01	1.19E-01	3.96E-01	3.40E-01	9.64E-01	9.23E-01
rs2373859	2p22.1	*SLC8A1*	^20^	797	4.48E-01	9.68E-01	2.75E-01	8.66E-01	1.98E-01	5.54E-01
rs11903757	2q32.3	*NABP1/SDPR*	^23^	4909	7.26E-01	2.92E-01	9.02E-01	2.66E-01	5.29E-01	5.34E-01
rs10936599	3q26.2	*TERC*	^13^	8296	5.38E-01	8.38E-01	7.26E-01	9.83E-01	9.48E-01	5.98E-01
rs35509282	4q32.2	*FSTL5*	^27^	1030	9.40E-01	7.64E-01	9.20E-01	8.85E-01	9.35E-01	9.10E-01
rs275454	5p15.31	*PAPD7*	^20^	0	4.71E-01	2.87E-01	6.38E-01	5.04E-01	9.34E-01	9.80E-01
rs2853668	5p15.33	*TERT*	^20^	0	5.61E-01	8.88E-01	4.40E-01	8.84E-01	5.15E-01	8.38E-01
rs647161	5q31.1	*PITX1/H2AFY*	^22^	0	**3.80E-03**	6.79E-02	**6.78E-03**	8.45E-02	7.56E-02	3.31E-01
rs1321311	6p21.2	*SRSF3/CDKN1A*	^19^	1541	2.62E-01	9.68E-01	1.88E-01	7.48E-01	3.09E-01	5.60E-01
rs1525461	7q35	*TPK1*	^20^	3217	4.43E-01	5.59E-01	2.97E-01	2.87E-01	2.33E-01	1.84E-01
rs16888522	8q23.3	*EIF3H*	^20^	1580	8.15E-02	7.24E-02	2.27E-01	2.52E-01	9.43E-01	6.36E-01
rs16892766^*^	8q23.3	*TRPS1/EIF3H/UTP23*	^10^	0	**7.75E-03**	**5.48E-04**	**3.67E-02**	**3.23E-03**	4.64E-01	5.74E-01
rs10505477	8q24.21	*CASC8*	^25^	0	**6.08E-04**	**3.48E-03**	**5.44E-03**	**5.10E-02**	3.38E-01	8.22E-01
rs10808555	8q24.21	*CASC8, MYC*	^11^	0	**3.20E-03**	**2.08E-02**	**1.22E-02**	1.28E-01	2.54E-01	9.78E-01
rs6983267^*^	8q24.21	*CASC8, MYC*	^4^	0	**7.35E-04**	**3.03E-03**	**5.10E-03**	**4.36E-02**	3.03E-01	9.34E-01
rs7014346	8q24.21	*CASC8*	^9^	0	**2.31E-03**	**1.26E-02**	**4.42E-03**	**3.91E-02**	9.48E-02	5.69E-01
rs7837328	8q24.21	*CASC8*	^11^	214	**7.89E-03**	9.95E-02	**4.86E-03**	1.43E-01	**4.49E-02**	6.62E-01
rs719725	9p24.1	*TPD52L3/UHRF2/GLDC*	^6^	34073	3.07E-01	5.52E-01	2.16E-01	3.59E-01	2.57E-01	2.28E-01
rs10795668	10p14	*KRT8P16/TCEB1P3*	^10^	0	3.26E-01	2.32E-01	1.56E-01	1.40E-01	1.65E-01	2.58E-01
rs704017	10q23.2	*ZMIZ1-AS1*	^29^	10425	6.97E-02	**2.32E-02**	1.65E-01	2.08E-01	9.04E-01	7.31E-01
rs1035209	10q24.2	*ABCC2/MRP2*	^26^	0	4.37E-01	5.15E-01	7.78E-01	9.56E-01	6.37E-01	4.96E-01
rs11196172	10q25.2	*TCF7L2*	^29^	224	8.12E-01	6.13E-01	5.86E-01	7.73E-01	2.26E-01	7.71E-01
rs12241008	10q25.2	*VTI1A*	^28^	513	3.40E-01	5.47E-01	6.91E-01	8.73E-01	4.73E-01	1.92E-01
rs1665650	10q26.2	*HSPA12A*	^22^	1647	8.07E-01	5.09E-01	5.57E-01	8.96E-01	3.95E-01	8.20E-01
rs1535	11q12.2	*FADS2*	^29^	7243	2.21E-01	**2.31E-02**	4.43E-01	**3.77E-02**	9.01E-01	4.78E-01
rs174550	11q12.2	*FADS1*	^29^	96	2.15E-01	**2.18E-02**	3.55E-01	**3.99E-02**	9.99E-01	4.17E-01
rs4246215	11q12.2	*FEN1*	^17^	5531	2.13E-01	**3.07E-02**	3.40E-01	5.52E-02	9.63E-01	4.76E-01
rs3824999	11q13.4	*POLD3*	^19^	1383	1.26E-01	**2.32E-02**	2.23E-01	5.58E-02	6.20E-01	4.41E-01
rs3802842^*^	11q23.1	*COLCA1,2*	^7^	0	**8.98E-05**	**8.62E-05**	**1.11E-04**	**1.86E-05**	**4.85E-03**	**2.91E-03**
rs10849432	12p13.31	*CD9*	^29^	1952	6.48E-01	7.73E-01	9.37E-01	3.89E-01	6.47E-01	2.32E-01
rs10774214	12p13.32	*CCND2*	^22^	1816	**3.85E-02**	**4.02E-02**	6.87E-02	**3.68E-02**	3.37E-01	4.48E-01
rs3217810	12p13.32	*CCND2*	^23^	887	4.27E-01	1.78E-01	2.74E-01	**2.81E-02**	2.94E-01	6.89E-02
rs3217901	12p13.32	*CCND2*	^23^	0	3.20E-01	3.11E-01	2.41E-01	2.18E-01	1.65E-01	3.00E-01
rs11169552	12q13.12	*ATF1*	^13^	0	5.08E-01	3.61E-01	2.72E-01	**4.37E-02**	2.05E-01	**4.71E-02**
rs7136702	12q13.12	*LARP4/DIP2B*	^13^	1753	7.08E-01	5.18E-01	5.40E-01	2.69E-01	3.91E-01	2.14E-01
rs59336	12q24.21	*TBX3*	^23^	1817	5.48E-01	6.78E-01	7.01E-01	7.19E-01	9.24E-01	7.89E-01
rs7315438	12q24.21	*TBX3*	^20^	481	1.47E-01	2.54E-01	2.78E-01	1.82E-01	5.32E-01	4.60E-01
rs1957636	14q22.2	*BMP4/ATP5C1P1/CDKN3/MIR5580*	^16^	3869	5.53E-01	1.18E-01	9.52E-01	3.92E-01	4.20E-01	7.87E-01
rs4444235^*^	14q22.2	*BMP4/ATP5C1P1/CDKN3/MIR5580*	^7^	0	6.22E-01	3.63E-01	9.03E-01	5.40E-01	4.58E-01	7.19E-01
rs11632715	15q13.3	*SCG5, GREM1, FMN1*	^16^	989	5.01E-01	3.09E-01	3.03E-01	1.58E-01	2.61E-01	3.17E-01
rs16969681	15q13.3	*SCG5, GREM1, FMN1*	^16^	0	5.25E-01	2.82E-01	5.79E-01	2.46E-01	8.45E-01	3.70E-01
rs4779584	15q13.3	*SCG5, GREM1, FMN1*	^7^	0	7.37E-02	**1.03E-02**	8.98E-02	**8.61E-03**	2.63E-01	5.18E-02
rs9929218	16q22.1	*CDH1/ZFP90*	^7^	0	7.72E-01	4.05E-01	5.89E-01	8.57E-01	1.52E-01	1.06E-01
rs12603526	17p13.3	*NXN*	^29^	0	2.93E-01	1.85E-01	1.14E-01	**4.43E-02**	1.03E-01	6.63E-02
rs12953717	18q21.1	*SMAD7*	^5^	0	**4.64E-04**	**2.83E-05**	**4.55E-04**	**8.64E-06**	**3.21E-02**	**5.04E-03**
rs4464148	18q21.1	*SMAD7*	^5^	82	6.75E-02	1.80E-01	**3.80E-02**	1.11E-01	1.08E-01	3.22E-01
rs4939827^*^	18q21.1	*SMAD7*	^7^	0	**8.37E-03**	**4.03E-04**	**9.92E-03**	**1.53E-04**	1.31E-01	**1.42E-02**
rs7229639	18q21.1	*SMAD7*	^25^	170	2.69E-01	9.11E-02	1.36E-01	**1.90E-02**	1.96E-01	**4.29E-02**
rs10411210	19q13.11	*RHPN2*	^7^	0	**3.94E-03**	**3.28E-04**	**2.07E-02**	**2.66E-03**	4.64E-01	2.91E-01
rs2241714	19q13.2	*TGFB1, B9D2*	^21^	12506	6.30E-01	8.95E-01	6.57E-01	9.93E-01	8.08E-01	8.30E-01
rs2423279	20p12.3	*BMP2/HAO1/FERMT1*	^22^	10815	8.46E-01	4.13E-01	7.65E-01	8.11E-01	3.03E-01	5.80E-01
rs4813802	20p12.3	*BMP2/HAO1/FERMT1*	^16^	0	**4.40E-02**	**3.49E-02**	5.59E-02	**1.88E-02**	2.09E-01	1.48E-01
rs961253	20p12.3	*BMP2/HAO1/FERMT1*	^7^	0	1.71E-01	1.12E-01	6.02E-01	4.29E-01	4.78E-01	3.46E-01
rs4925386	20q13.33	*LAMA5*	^13^	53263	9.93E-02	9.76E-02	**1.25E-02**	**4.64E-03**	**7.60E-03**	**1.11E-03**

P-values are uncorrected and P-values <0.05 (5.00E-02) are given in bold.

P-values <0.00089 (8.90E-04) are given in bold and are underlined.

Rs number followed by ^*^ indicates CRC SNP with experimentally confirmed functional relevance [52].

Several SNPs previously associated not only with risk of CRC but also with risk of colorectal adenoma exhibited borderline significant P-values in comparisons B vs. C and B vs. CD (rs7837328, P_(Bvs.C)_=4.49×10^-2^; rs3802842, P_(Bvs.C)_=4.85×10^-3^, P_(Bvs.CD)_=2.91×10^-3^; rs4939827, P_(Bvs.CD)_=1.42×10^-2^; rs4925386, P_(Bvs.C)_=7.60×10^-3^, P_(Bvs.CD)_=1.11×10^-3^).

## DISCUSSION

Most published GWAS are based on single marker analysis in combination with correction for multiple testing, a strategy which has been shown to suffer both from unnecessarily low power and a relatively high risk of false positive detections in case of complex traits [38]. Reduced statistical power reflects one aspect of missing heritability in GWAS [1]. Simulation studies based on real SNP data provided evidence that model selection strategies may outperform multiple testing in detecting causal SNPs [39] while controlling the type I error rate of false detections and therefore, should be used to complement (standard) analysis of GWAS.

We performed – to our best knowledge – the first GWAS of CRC in an Austrian cohort including 1060 CRC cases, 689 patients with advanced colorectal adenomas, 928 colonoscopy-negative controls, and additional genotype data of 3439 population-based KORA controls from southern Germany. Model selection analysis was based on MOSGWA [39], a bioinformatical tool for analysis of GWAS using the FDR controlling modification of BIC, mBIC2, which has been shown to have certain optimality properties with respect to the number of missclassifications. Due to its fixed selection criterion, MOSGWA requires no parameter tuning like LASSO-based approaches [40]. In simulation studies [39], MOSGWA exceeded the performance of competing approaches and when re-analyzing data of complex diseases from the Wellcome Trust Case-Control Consortium [41] several SNPs could be identified, which were not detected by other algorithms, but were later confirmed by independent studies [39].

In this study, MOSGWA selected models for different case-control comparisons, including between one and 14 SNPs. The theoretically well-founded advantage of the model selection approach is its larger power to detect candidate SNPs compared to single marker tests while at the same time strictly controlling the false discovery rate. Among all four studied contrasts, single marker tests yielded only one significant SNP (rs17659990, P=5.43×10^-9^, *DOCK3*) at the usually recommended genome-wide significance level for the comparison AB vs. CD when considering the entire study population. Rs17659990 is an intronic variant of dedicator of cytokinesis 3 (*DOCK3*) gene, a gene specifically expressed in the central nervous system, that was associated with an attention deficit hyperactivity disorder-like phenotype [42]. *DOCK3*, also referred to as modifier of cell adhesion (*MOCA*), was also shown to be an inhibitor of Wnt/beta-catenin signaling [43], a pathway known to play an important role in colorectal carcinogenesis [44]. Moreover, multiple studies reported *DOCK3* to be implicated in cancer cell invasion and migration (as recently reviewed [45]). The SNP rs17659990 was also included in the model A vs. CD (model size 7).

For the comparison AB vs. CD, MOSGWA selected a model including 14 SNPs, including apart from rs17659990 another borderline significant SNP (rs7742915, P=8.52×10^-8^, *BTBD9*). Rs7742915 of BTB domain containing 9 (*BTBD9*) gene, a locus encoding a BTB/POZ domain-containing protein, is involved in protein-protein interactions. Genetic variation of *BTBD9* was associated with susceptibility to Restless Legs Syndrome [46]. Aside from rs17659990 and rs7742915, further 12 variants with marginal P-values (P>5.0×10^-8^) were selected for AB vs. CD comparison including rs16944613 (P=1.49×10^-7^, *CRTC3*), rs13129679 (P=2.38×10^-7^, *RNF4*), and rs12953717 (P=3.00×10^-7^, *SMAD7*). Rs12953717 located in intron 3 of *SMAD7* gene has been previously linked to CRC risk by two GWAS [5, 9] and was subsequently confirmed as CRC susceptibility variant [47, 48] as recently discussed by Stolfi et al. [49]. *SMAD7* is a negative regulator of transforming growth factor-β signaling. Depending on single marker tests only, *SMAD7* rs12953717 may not have been regarded as a candidate SNP in our study.

Interestingly, rs1912804 of WW domain-containing oxidoreductase (*WWOX*) gene emerged in this study of CRC (A vs. C). Defects in this tumor suppressor gene were associated with multiple cancers [50] and altered *WWOX* expression was observed in tissues of CRC [51]. Recently, *WWOX* was shown to be involved in double-strand break repair [50]. Although defects in mismatch repair (MMR) genes influence both, hereditary and sporadic CRCs (recently reviewed [52]), no CRC risk SNPs annotating to MMR genes were identified by GWAS thus far.

In this study, we used model selection as a tool to detect SNPs associated with CRC, not aiming at the identification of a model which can be used later for prediction. Therefore, we do not provide model coefficients obtained by MOSGWA but only report the detected SNPs. This is crucial to understand the principle and function of model selection as tool for analysis of GWAS. Considering the identification of disease associated SNPs as a high-dimensional classification problem, SNPs can be classified as either associated or not associated with the trait. Theoretical results showed that performing model selection using the FDR controlling mBIC2 selection criterion yields a classification procedure which asymptotically minimizes the misclassification rate. The expected proportion of false positive SNPs is controlled at a level which decreases with sample size and which will be for this study below 5%. Therefore, about one or two false positive detections can be expected among the reported 14 SNPs in model AB vs. CD.

CRC SNPs identified by preceding GWAS were tested in a hypothesis-driven approach and a number of these SNPs exhibited relevant differences between cases and controls in our data set. Several risk variants were replicated in this study for the first time in the Austrian population. The strongest associations were observed for SNPs annotating to the following genes: *SMAD7, RHPN2, EIF3H, CASC8, MYC*, and *COLCA1,2*. Functional relevance was experimentally confirmed for only five common CRC risk loci [52]. Four of them (rs16892766, *EIF3H*; rs6983267, *MYC*; rs3802842, *COLCA2* and rs4939827, *SMAD7*) also play a role in our study population.

Sporadic CRCs usually arise from premalignant lesions (adenoma-carcinoma sequence), thus high-risk adenomas impact CRC risk [53, 54]. Removal of advanced adenomas during colonoscopy reduces mortality from CRC [55]. We included advanced colorectal adenomas into this study because these precursors are important targets for CRC prevention. Previously unreported rs7944251 of FAT tumor suppressor homolog 3 (*FAT3*) was associated with reduced risk of advanced adenoma (OR=0.66, P=3.97×10^-7^) and the SNP was also selected when comparing advanced adenomas with the combined control group (B vs. CD). All previously unreported candidate SNPs demand replication in independent CRC cohorts.

A strength of this study is the dual approach to analyze genotype distributions in a genome-wide SNP dataset including CRC cases, advanced adenomas and controls. CORSA controls (C) received a complete colonoscopy within B-PREDICT screening and were known to be free of colorectal polyps and CRC. Sometimes, these colonoscopy-negative controls are also referred to as “super-controls” [12]. A recent study indicated that exclusion of controls with a family history of CRC and of controls with record of colorectal adenomas can increase power [56]. To our knowledge, this is the first GWAS of CRC investigating Austrian CRC cases and premalignant colorectal tumors. However, limitations of the study are the limited sample size, especially in the subgroup of advanced adenomas as well as limited availability of environmental data of CRC cases impeding stratification analysis for environmental risk factors. To increase statistical power, individual level genotype data of additional controls (KORA) were included in the study. Because CORSA recruitment is ongoing, further Austrian CRC cases will be genotyped and integrated into the analysis to investigate population specific SNP signatures of CRC risk. Meta-analysis of GWAS present a powerful strategy to enhance the power of identifying weak genetic associations with disease phenotype, but is often complicated by between-study heterogeneity. Precision gained by combination of datasets may be spurious due to different study designs, divergent LD structures, different patterns of correlated phenotypes or dissimilar gene-environment interactions across populations [57, 58].

The application of CRC SNP signatures to improve screening decisions is presently impeded by the fact that single risk variants account only for little heritability and thereby explain a small increment of risk. We hypothesize that potentially disease relevant variants not reaching genome-wide significance may explain a substantial part of missing heritability and are worth exploration and follow-up. Also epigenetic alterations play an important role in colorectal carcinogenesis [59]. The combination of genetic and epigenetic biomarkers to a multi-marker panel considering also environmental risk factors could be suited to complement present screening strategies and for instance be applied after a positive fecal occult blood test, but prior to an invasive colonoscopy. Genetic risk variants are ideal candidates for the development of minimal-invasive and cost-effective biomarker tests enabling personal risk profiling. In the near future, management of CRC will increasingly focus on personalized screening and treatment strategies aiming at early detection and prevention of disease. A combination of single marker tests and model selection in high dimensions may facilitate the identification of marker candidates otherwise not detected due to stringent penalties for multiple testing.

## MATERIALS AND METHODS

### Study population

In this GWAS, 2677 individuals of our ongoing Colorectal Cancer Study of Austria (CORSA) [60, 61] were genotyped including 1060 CRC cases, 689 patients with advanced adenomas and 928 colonoscopy-negative controls. CRC cases were patients with histologically confirmed, sporadic CRC. CRC cases with clinical record of inflammatory bowel disease (IBD) were excluded from the study. Advanced adenomas included adenomatous villous, adenomatous tubulovillous and tubular polyps larger than 1cm in diameter. All controls received a complete colonoscopy and exhibited no pathological findings.

From June 2003 to November 2012 CORSA participants had been recruited in four hospitals in the province Burgenland (Oberpullendorf, Kittsee, Oberwart and Güssing), Austria, at the Medical University of Vienna (Department of Surgery), and the Medical University of Graz (Department of Internal Medicine).

To augment statistical power, individual level genotype data of 3439 additional control individuals from the German “Cooperative Health Research in the Region of Augsburg” (KORA) platform were included in this study [62]. Population-based controls from the studies S4 and F4 were integrated. To ensure exclusion of CRC cases from the KORA control set, all individuals with evidence of malignant diseases were removed from the dataset. In total, 6116 individuals (1749 colorectal tumors and 4367 controls) were included in this study.

### Ethics statement

Written informed consent was obtained from all participants of CORSA. The study was approved by the ethical review committee of the Medical University of Vienna (MUW, EK Nr. 703/2010) and the “Ethikkommission Burgenland” (KRAGES, 33/2010). Conduct of the study followed the approved study protocol and all methods were performed in accordance with the relevant guidelines and regulations. Approval for the use of KORA data was obtained from the KORA-Study Group (K072/13).

### Genotyping

Genomic DNA was purified from peripheral blood following the QIAamp DNA Blood Midi Spin Protocol (QIAGEN, Valencia, CA). Genotyping was performed using population-optimized Axiom Genome-Wide CEU 1 Arrays (Affymetrix, Santa Clara, CA) analyzing 587,532 SNPs. Array processing was performed at the Institute of Human Genetics, Helmholtz Center Munich. KORA samples were genotyped on the same array type.

### Statistical analysis

Extensive quality control and genotype calling was performed with Affymetrix Genotyping Console Software 4.1.3.840 (www.affymetrix.com). 2469 genotyped CORSA subjects survived QC filtering (Dish QC >0.82, call rate >97.5%).

Inclusion criteria for SNPs eligible for downstream analysis were a minor allele frequency (MAF) >1%, Hardy-Weinberg equilibrium (HWE) P-value cut-off >1.00×10^-8^, a SNP call rate >97.5%, and >95% calls per individual. 271 SNPs were discarded due to showing significant difference between the CORSA and KORA control group (P-values smaller than 1.00×10^-7^ in a simple Fisher exact test comparing controls as suggested in [63]). After filtering, 492,217 SNPs remained for which imputation of missing genotypes was performed using Beagle software v.4.0 r1274 [64].

The primary aim of the study was to find SNPs which are associated with CRC or with advanced adenomas, respectively. To this end we performed traditional single marker based analysis as well as a more involved model selection based approach. Single marker analysis was performed with PLINK 1.9 beta 3 (www.cog-genomics.org/plink2) [65]. We report P-values of CAT as well as from a logistic regression model including the factors age and the leading four principal components from a principal component analysis (PCA) which was used to adjust for population structure [66]. A PCA plot of the first four principal components plotted against each other is provided in Supplementary Figure 1. Genotype cluster plots of all reported SNPs underwent visual inspection.

For model selection analysis, the software package MOSGWA was applied (http://mosgwa.sourceforge.net) [39] using multi-marker logistic regression models including again the factors age and the leading four principal components as covariates which were not under selection. In addition to the genome-wide analysis we inspected specifically 56 SNPs which were previously reported in the GWAS literature to be involved in colorectal carcinogenesis. For SNPs not represented on the array, suitable proxies were identified and tested.

## SUPPLEMENTARY MATERIALS FIGURES AND TABLES




