# Supplementary material for: Bayesian and frequentist analysis of an Austrian genome-wide association study of colorectal cancer and advanced adenomas
Source: Oncotarget. 2017 Oct 9;8(58):98623–34. doi: 10.18632/oncotarget.21697 (PMC5716755; doi:10.18632/oncotarget.21697)
Supplement: Supplementary file 1 [file oncotarget-08-98623-s001.pdf]

Bayesian and frequentist analysis of an Austrian genome-wide association study of colorectal cancer and advanced adenomas

SUPPLEMENTARY MATERIALS

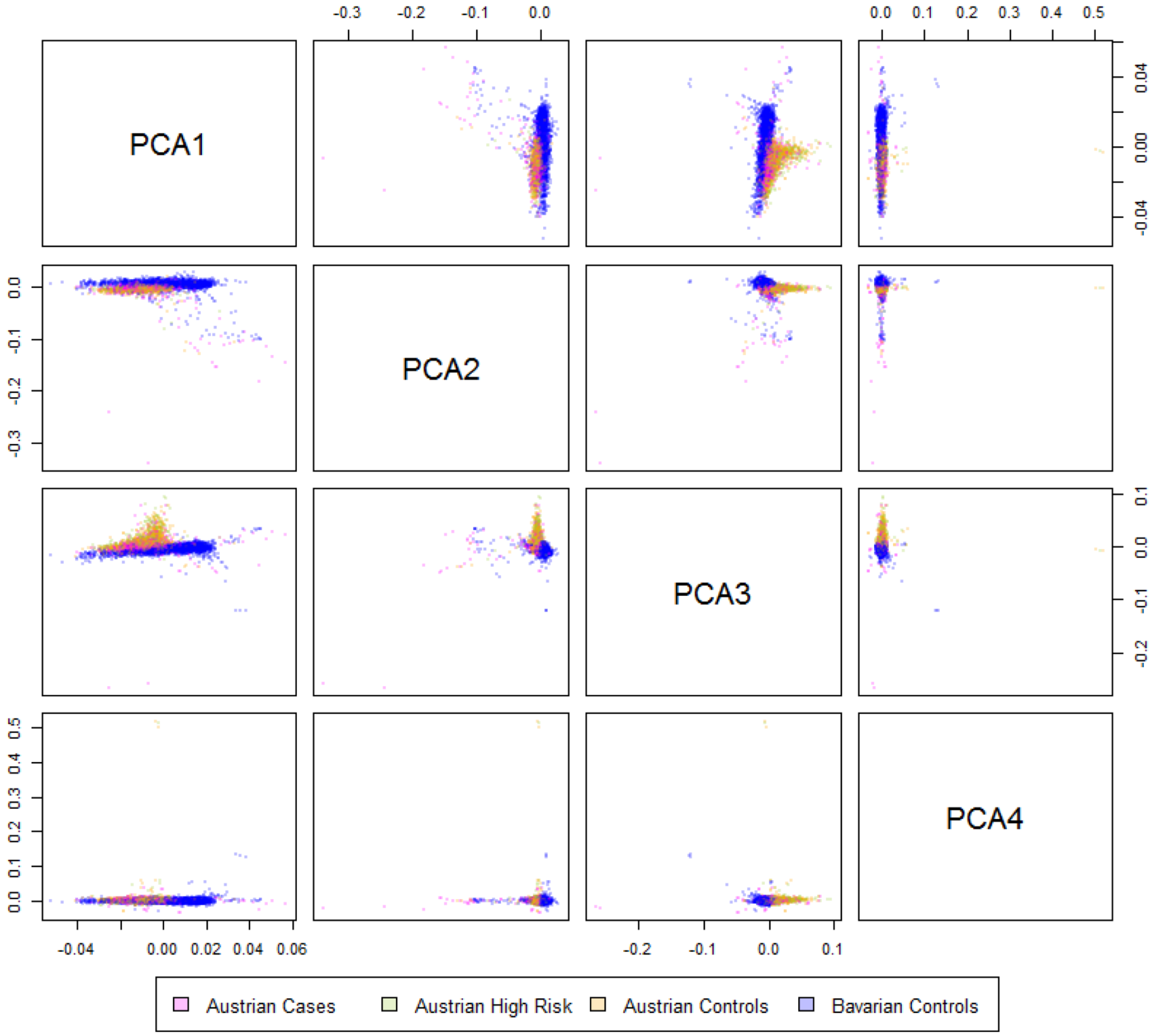

Supplementary Figure 1: PCA plot. The first 4 PCs are plotted against each other. Case-control status is indicated by coloured shading.

Supplementary Table 1: UICC staging and localization of post-QC samples

	CRC (A)	AA (B)
**UICC Stage**		
**I**	153	-
**II**	224	-
**III**	376	-
**IV**	112	-
**X**[1]	66	-
**Missing**	47	-
**Location**		
**Colon – distal**[2]	296	288
**Colon – proximal**[3]	257	215
**Rectal**[4]	401	113
**Missing**	24	20
**Amount of polpys**		
**one**	-	436
**two**	-	93
**more than two**	-	88
**Missing**	-	19

CRC Colorectal cancer cases.
AA Advanced adenomas.
[1] UICC staging not possible (e.g. incomplete TNM or palliative surgery).
[2] sigmoid colon, descending colon, splenic flexure.
[3] transverse colon, hepatic flexure, ascending colon, cecum, appendix.
[4] rectum, rectosigmoid junction.

Supplementary Table 2: The 200 top ranking SNPs for each case-control contrast. The four different case and control groups were colorectal cancer cases **(A)**, advanced colorectal adenomas **(B)**, CORSA controls **(C)** and KORA controls **(D)**.

See Supplementary File 1
