# Supplementary material for: Bayesian and frequentist analysis of an Austrian genome-wide association study of colorectal cancer and advanced adenomas
Source: Oncotarget. 2017 Oct 9;8(58):98623–34. doi: 10.18632/oncotarget.21697 (PMC5716755; doi:10.18632/oncotarget.21697)
Supplement: Supplementary file 2 [file oncotarget-08-98623-s002.docx]

**Supplementary Table 2: The 200 top ranking SNPs for each case-control contrast**

| **A vs. C** | | | | | | |
| --- | --- | --- | --- | --- | --- | --- |
| RANK | SNP | RS ID | CHR | BP | OR | P |
| 1 | AX-11352279 | rs1912804 | 16 | 78626583 | 1.693 | 3.39E-07 |
| 2 | AX-11696145 | rs9583269 | 13 | 109283030 | 0.6931 | 6.19E-07 |
| 3 | AX-11285363 | rs16869961 | 4 | 20780315 | 0.7049 | 1.24E-06 |
| 4 | AX-11669320 | rs845868 | 6 | 998813 | 0.6818 | 2.11E-06 |
| 5 | AX-11269952 | rs1505758 | 2 | 117078901 | 0.7233 | 2.53E-06 |
| 6 | AX-11364453 | rs2062961 | 2 | 117077958 | 0.7251 | 2.62E-06 |
| 7 | AX-11111213 | rs10495672 | 2 | 18494455 | 1.433 | 3.23E-06 |
| 8 | AX-11633012 | rs7604753 | 2 | 117135650 | 0.721 | 3.40E-06 |
| 9 | AX-11145214 | rs11123333 | 2 | 116909251 | 0.7269 | 3.56E-06 |
| 10 | AX-11530867 | rs4849449 | 2 | 116955767 | 0.7283 | 3.84E-06 |
| 11 | AX-11693976 | rs954980 | 9 | 3761909 | 0.7177 | 5.26E-06 |
| 12 | AX-11280329 | rs1654668 | 19 | 55174213 | 0.7347 | 6.05E-06 |
| 13 | AX-11648584 | rs7831492 | 8 | 41614503 | 0.73 | 6.11E-06 |
| 14 | AX-11331925 | rs17626293 | 1 | 195651519 | 0.4726 | 7.08E-06 |
| 15 | AX-11128916 | rs10878977 | 12 | 69799690 | 1.407 | 8.15E-06 |
| 16 | AX-11609081 | rs7134911 | 12 | 30975469 | 0.7384 | 9.38E-06 |
| 17 | AX-11595761 | rs6937873 | 6 | 998948 | 0.7237 | 9.89E-06 |
| 18 | AX-11125859 | rs10838159 | 11 | 43762210 | 0.7108 | 1.11E-05 |
| 19 | AX-11633133 | rs7606512 | 2 | 235609674 | 2.248 | 1.18E-05 |
| 20 | AX-11378193 | rs2244884 | 21 | 19709819 | 1.425 | 1.24E-05 |
| 21 | AX-11586365 | rs6800634 | 3 | 154723947 | 1.347 | 1.26E-05 |
| 22 | AX-11426967 | rs2918101 | 10 | 132984326 | 0.7346 | 1.61E-05 |
| 23 | AX-11133204 | rs10941252 | 5 | 35643615 | 1.357 | 1.66E-05 |
| 24 | AX-11631811 | rs7588002 | 2 | 117200339 | 0.7452 | 1.77E-05 |
| 25 | AX-11489822 | rs40719 | 5 | 9171202 | 0.7481 | 1.79E-05 |
| 26 | AX-11269926 | rs1505521 | 8 | 118076115 | 2.188 | 1.82E-05 |
| 27 | AX-11274913 | rs1554395 | 3 | 183664873 | 0.7215 | 1.82E-05 |
| 28 | AX-11421506 | rs28655514 | 14 | 62493964 | 1.837 | 1.90E-05 |
| 29 | AX-11540334 | rs4979489 | 9 | 117768680 | 0.7184 | 1.93E-05 |
| 30 | AX-11109554 | rs10485879 | 7 | 77905130 | 1.458 | 1.96E-05 |
| 31 | AX-11465091 | rs35505947 | 18 | 13954109 | 0.6703 | 2.01E-05 |
| 32 | AX-11577254 | rs6668441 | 1 | 102242917 | 0.4924 | 2.10E-05 |
| 33 | AX-11150713 | rs11205451 | 1 | 48647860 | 2.768 | 2.38E-05 |
| 34 | AX-11349269 | rs1877720 | 1 | 48824407 | 2.372 | 2.43E-05 |
| 35 | AX-11310150 | rs17146516 | 7 | 123597121 | 0.6809 | 2.65E-05 |
| 36 | AX-11458231 | N/A | 17 | 32288423 | 0.652 | 2.74E-05 |
| 37 | AX-11606183 | rs7091587 | 10 | 83600326 | 0.5652 | 2.76E-05 |
| 38 | AX-11187081 | rs12029406 | 1 | 199905828 | 1.334 | 2.77E-05 |
| 39 | AX-11336162 | rs17707229 | 16 | 78585339 | 1.698 | 2.83E-05 |
| 40 | AX-11401706 | rs2619283 | 3 | 101890895 | 1.331 | 2.90E-05 |
| 41 | AX-11306651 | rs17106529 | 12 | 69804612 | 1.554 | 3.11E-05 |
| 42 | AX-11510652 | rs4552773 | 6 | 8794834 | 0.5013 | 3.11E-05 |
| 43 | AX-11713749 | rs9977229 | 21 | 19689010 | 1.448 | 3.13E-05 |
| 44 | AX-11353136 | rs1924717 | 1 | 195263620 | 1.328 | 3.14E-05 |
| 45 | AX-11135614 | rs10974595 | 9 | 4527121 | 1.856 | 3.15E-05 |
| 46 | AX-11232873 | rs12936143 | 17 | 2556971 | 1.379 | 3.24E-05 |
| 47 | AX-11322058 | rs17382698 | 8 | 129139206 | 1.533 | 3.32E-05 |
| 48 | AX-11605224 | rs7077543 | 10 | 56111483 | 0.712 | 3.46E-05 |
| 49 | AX-11632286 | rs759455 | 2 | 206505305 | 1.321 | 3.94E-05 |
| 50 | AX-11334896 | rs17682748 | 6 | 6571187 | 0.4759 | 4.01E-05 |
| 51 | AX-11086712 | rs10002854 | 4 | 7521146 | 0.4158 | 4.13E-05 |
| 52 | AX-11087310 | rs10012525 | 4 | 16912717 | 0.5525 | 4.28E-05 |
| 53 | AX-11514909 | rs4644524 | 1 | 199945630 | 0.7512 | 4.33E-05 |
| 54 | AX-11580164 | rs6713607 | 2 | 226372129 | 0.7436 | 4.37E-05 |
| 55 | AX-11373235 | rs2183478 | 1 | 244314952 | 0.6788 | 4.53E-05 |
| 56 | AX-11707736 | rs9875549 | 3 | 95886501 | 1.318 | 4.54E-05 |
| 57 | AX-11169308 | rs11707642 | 3 | 193820138 | 0.646 | 4.63E-05 |
| 58 | AX-11384978 | rs2325858 | 3 | 95908409 | 1.317 | 4.65E-05 |
| 59 | AX-11293371 | rs16958219 | 15 | 47012483 | 2.144 | 4.78E-05 |
| 60 | AX-11165981 | rs11659657 | 18 | 13989970 | 0.6825 | 4.80E-05 |
| 61 | AX-11322385 | rs17390391 | 1 | 80868144 | 1.656 | 4.97E-05 |
| 62 | AX-11588070 | rs6824647 | 4 | 26177372 | 0.7124 | 4.97E-05 |
| 63 | AX-11516168 | rs4663979 | 2 | 234803432 | 0.7455 | 5.15E-05 |
| 64 | AX-11208681 | rs12473004 | 2 | 234808569 | 0.7143 | 5.30E-05 |
| 65 | AX-11696256 | rs9585596 | 13 | 101671500 | 1.577 | 5.32E-05 |
| 66 | AX-11619933 | rs7303284 | 12 | 104707546 | 2.092 | 5.42E-05 |
| 67 | AX-11640967 | rs7719458 | 5 | 35703042 | 1.324 | 5.48E-05 |
| 68 | AX-11450205 | rs34628136 | 3 | 192533897 | 1.626 | 5.50E-05 |
| 69 | AX-11528764 | rs4820887 | 22 | 31016914 | 1.707 | 5.52E-05 |
| 70 | AX-11599162 | rs6984966 | 8 | 38736089 | 0.7633 | 5.55E-05 |
| 71 | AX-11657883 | rs798300 | 7 | 77953248 | 1.402 | 5.58E-05 |
| 72 | AX-11181573 | rs11911274 | 21 | 19702383 | 1.495 | 5.76E-05 |
| 73 | AX-11342292 | rs1782633 | 6 | 161249326 | 1.517 | 5.91E-05 |
| 74 | AX-11339042 | rs1776284 | 1 | 80738763 | 1.493 | 6.00E-05 |
| 75 | AX-11387116 | rs2361095 | 9 | 634862 | 1.575 | 6.02E-05 |
| 76 | AX-11103328 | rs10271676 | 7 | 17808141 | 0.7632 | 6.06E-05 |
| 77 | AX-11423669 | rs2876841 | 7 | 39537647 | 0.6344 | 6.15E-05 |
| 78 | AX-11394655 | rs2489944 | 6 | 161292913 | 1.473 | 6.17E-05 |
| 79 | AX-11127442 | rs10860021 | 12 | 96747710 | 1.332 | 6.19E-05 |
| 80 | AX-11621921 | rs7330717 | 13 | 114948574 | 0.7534 | 6.47E-05 |
| 81 | AX-11324205 | rs17437573 | 2 | 165328097 | 0.5926 | 6.57E-05 |
| 82 | AX-11476083 | rs366676 | 6 | 88717766 | 0.764 | 6.66E-05 |
| 83 | AX-11158490 | rs11539086 | 3 | 58552329 | 1.665 | 6.69E-05 |
| 84 | AX-11545277 | rs563487 | 15 | 46933050 | 1.308 | 6.72E-05 |
| 85 | AX-11292308 | rs16947390 | 16 | 49452087 | 1.937 | 6.77E-05 |
| 86 | AX-11095544 | rs10141419 | 14 | 89454718 | 0.3515 | 6.81E-05 |
| 87 | AX-11480877 | rs3790843 | 1 | 200010824 | 1.34 | 6.81E-05 |
| 88 | AX-11434068 | rs3110616 | 8 | 59711710 | 1.367 | 6.95E-05 |
| 89 | AX-11662062 | rs8043910 | 16 | 78698238 | 1.804 | 6.97E-05 |
| 90 | AX-11145139 | rs11122189 | 1 | 231145185 | 0.7551 | 7.06E-05 |
| 91 | AX-11530890 | rs4849925 | 2 | 3814425 | 0.737 | 7.09E-05 |
| 92 | AX-11159130 | rs1155750 | 6 | 54730344 | 0.6929 | 7.15E-05 |
| 93 | AX-11122068 | rs10790917 | 11 | 127645687 | 0.7653 | 7.31E-05 |
| 94 | AX-11515645 | rs4657015 | 1 | 161272441 | 1.313 | 7.33E-05 |
| 95 | AX-11209760 | rs12489230 | 3 | 169558821 | 0.7364 | 7.33E-05 |
| 96 | AX-11612691 | rs7182621 | 15 | 100424605 | 1.318 | 7.36E-05 |
| 97 | AX-11367511 | rs2105447 | 1 | 80814734 | 1.657 | 7.42E-05 |
| 98 | AX-11231906 | rs12915935 | 15 | 100412080 | 1.336 | 7.46E-05 |
| 99 | AX-11681807 | rs9341388 | 6 | 73506104 | 0.6085 | 7.48E-05 |
| 100 | AX-11099753 | rs1020806 | 11 | 112536152 | 1.385 | 7.53E-05 |
| 101 | AX-11121255 | rs10780947 | 9 | 73165078 | 0.752 | 7.53E-05 |
| 102 | AX-11559508 | rs6103012 | 20 | 41350758 | 1.786 | 7.62E-05 |
| 103 | AX-11134756 | rs10962619 | 9 | 16815861 | 0.7004 | 7.64E-05 |
| 104 | AX-11266725 | rs1472081 | 17 | 27395890 | 1.344 | 7.70E-05 |
| 105 | AX-11315696 | rs17239917 | 15 | 90333851 | 0.702 | 7.82E-05 |
| 106 | AX-11631270 | rs7580256 | 2 | 116918533 | 0.7592 | 7.87E-05 |
| 107 | AX-11587082 | rs6810643 | 4 | 187687030 | 1.317 | 7.96E-05 |
| 108 | AX-11162432 | rs11612399 | 12 | 97826927 | 0.4699 | 8.18E-05 |
| 109 | AX-11606272 | rs7092884 | 10 | 93483872 | 0.765 | 8.25E-05 |
| 110 | AX-11255120 | rs1348964 | 11 | 45663536 | 0.7584 | 8.29E-05 |
| 111 | AX-11547916 | rs582962 | 6 | 69521978 | 1.32 | 8.37E-05 |
| 112 | AX-11467387 | rs35637782 | 4 | 135640298 | 1.52 | 8.38E-05 |
| 113 | AX-11546267 | rs5750616 | 22 | 38918894 | 0.7384 | 8.43E-05 |
| 114 | AX-11526904 | rs4795487 | 17 | 27344430 | 1.402 | 8.48E-05 |
| 115 | AX-11094456 | rs10125105 | 9 | 111226188 | 1.295 | 8.49E-05 |
| 116 | AX-11313371 | rs17193172 | 17 | 32287763 | 0.6699 | 8.51E-05 |
| 117 | AX-11375405 | rs221170 | 7 | 29005441 | 1.397 | 8.58E-05 |
| 118 | AX-11159092 | rs11556518 | 3 | 184082931 | 1.931 | 8.63E-05 |
| 119 | AX-11132037 | rs10925648 | 1 | 238588516 | 0.468 | 8.97E-05 |
| 120 | AX-11394547 | rs2487423 | 6 | 70526777 | 0.7714 | 9.12E-05 |
| 121 | AX-11600602 | rs7003661 | 8 | 38727464 | 1.3 | 9.12E-05 |
| 122 | AX-11707540 | N/A | 3 | 142378798 | 1.787 | 9.42E-05 |
| 123 | AX-11641723 | rs7731626 | 5 | 55444683 | 0.7689 | 9.69E-05 |
| 124 | AX-11696118 | rs958267 | 3 | 95857855 | 0.7657 | 9.74E-05 |
| 125 | AX-11641181 | rs7722488 | 5 | 116268205 | 0.6755 | 0.0001005 |
| 126 | AX-11323303 | rs17414682 | 2 | 104140200 | 0.5437 | 0.0001011 |
| 127 | AX-11090967 | rs10072484 | 5 | 35707707 | 1.308 | 0.0001017 |
| 128 | AX-11206664 | rs12444232 | 16 | 20321467 | 0.536 | 0.0001025 |
| 129 | AX-11680168 | rs9313333 | 5 | 166038872 | 3.582 | 0.000104 |
| 130 | AX-11654710 | rs7935243 | 11 | 44094416 | 0.7671 | 0.0001045 |
| 131 | AX-11562471 | rs635926 | 1 | 187933982 | 0.6898 | 0.0001077 |
| 132 | AX-11123010 | rs10802909 | 1 | 240985581 | 0.7441 | 0.0001089 |
| 133 | AX-11673612 | rs903610 | 3 | 30468597 | 1.478 | 0.0001089 |
| 134 | AX-11308820 | rs17130693 | 1 | 68908869 | 0.5495 | 0.0001109 |
| 135 | AX-11534602 | rs4899192 | 14 | 66500182 | 0.4064 | 0.0001115 |
| 136 | AX-11407577 | rs2726595 | 8 | 59710250 | 1.403 | 0.0001133 |
| 137 | AX-11325175 | rs17461542 | 5 | 148310477 | 1.313 | 0.0001134 |
| 138 | AX-11264214 | rs1444867 | 3 | 55396479 | 1.91 | 0.0001151 |
| 139 | AX-11240755 | rs13127946 | 4 | 37042611 | 0.6971 | 0.0001165 |
| 140 | AX-11199103 | rs12285517 | 11 | 98195448 | 1.944 | 0.0001172 |
| 141 | AX-11244415 | rs13208448 | 6 | 21304987 | 0.4627 | 0.0001173 |
| 142 | AX-11112000 | rs10500019 | 7 | 112780408 | 1.511 | 0.0001173 |
| 143 | AX-11280889 | rs1672713 | 11 | 113955678 | 1.391 | 0.0001183 |
| 144 | AX-11333714 | rs17659990 | 3 | 50751149 | 1.901 | 0.0001186 |
| 145 | AX-11089439 | rs10047918 | 14 | 69172166 | 0.7307 | 0.0001189 |
| 146 | AX-11302642 | rs17064859 | 18 | 56020793 | 0.6015 | 0.0001196 |
| 147 | AX-11445099 | rs34328687 | 9 | 18608800 | 1.606 | 0.0001197 |
| 148 | AX-11299225 | rs17028348 | 2 | 64263931 | 0.2957 | 0.0001216 |
| 149 | AX-11350821 | rs1891785 | 10 | 132859229 | 1.367 | 0.0001234 |
| 150 | AX-11691173 | rs9514535 | 13 | 107088897 | 0.7726 | 0.0001236 |
| 151 | AX-11133972 | rs10952705 | 7 | 147417555 | 0.7608 | 0.0001244 |
| 152 | AX-11495372 | rs4141669 | 10 | 56070319 | 0.667 | 0.0001246 |
| 153 | AX-11307008 | rs17110136 | 14 | 40447949 | 0.6116 | 0.0001263 |
| 154 | AX-11425145 | rs2891240 | 9 | 25033044 | 1.293 | 0.0001267 |
| 155 | AX-11544732 | rs554096 | 18 | 66887311 | 1.345 | 0.0001286 |
| 156 | AX-11354464 | rs1941098 | 18 | 13988795 | 0.7227 | 0.0001304 |
| 157 | AX-11591717 | rs6879983 | 5 | 88935920 | 1.445 | 0.000132 |
| 158 | AX-11543565 | rs534170 | 15 | 46933950 | 1.3 | 0.0001327 |
| 159 | AX-11643869 | rs7762407 | 6 | 154165415 | 0.6979 | 0.0001339 |
| 160 | AX-11572533 | rs656730 | 11 | 94342444 | 0.6968 | 0.0001345 |
| 161 | AX-11634217 | rs762158 | 21 | 35653670 | 1.341 | 0.0001352 |
| 162 | AX-11357290 | rs1980428 | 11 | 9010497 | 1.292 | 0.0001368 |
| 163 | AX-11243153 | rs13180075 | 5 | 28079752 | 0.7698 | 0.0001368 |
| 164 | AX-11570146 | rs6534458 | 4 | 125976652 | 2.202 | 0.0001393 |
| 165 | AX-11179761 | rs11872901 | 18 | 62367856 | 1.327 | 0.0001397 |
| 166 | AX-11102415 | rs10254660 | 7 | 123625077 | 0.7692 | 0.0001408 |
| 167 | AX-11179592 | rs11869615 | 17 | 32245868 | 1.295 | 0.0001425 |
| 168 | AX-11167432 | rs11680720 | 2 | 125093388 | 1.871 | 0.0001428 |
| 169 | AX-11546699 | rs5761119 | 22 | 25953851 | 2.031 | 0.0001433 |
| 170 | AX-11658064 | rs7985095 | 13 | 108681130 | 1.311 | 0.0001437 |
| 171 | AX-11409418 | rs2764095 | 6 | 7329873 | 0.7486 | 0.0001441 |
| 172 | AX-11567409 | rs6485905 | 11 | 12602389 | 0.7369 | 0.0001445 |
| 173 | AX-11641792 | rs7732774 | 5 | 159036818 | 0.7264 | 0.0001464 |
| 174 | AX-11572762 | rs6570675 | 6 | 145523536 | 1.324 | 0.0001477 |
| 175 | AX-11148067 | rs11161729 | 1 | 86551864 | 0.7735 | 0.0001498 |
| 176 | AX-11357719 | rs1984791 | 11 | 9035027 | 1.323 | 0.0001515 |
| 177 | AX-11535269 | rs4907104 | 1 | 85316955 | 0.7677 | 0.0001517 |
| 178 | AX-11595293 | rs6931801 | 6 | 143961610 | 1.446 | 0.0001525 |
| 179 | AX-11568222 | rs6496649 | 15 | 90849518 | 0.7727 | 0.0001527 |
| 180 | AX-11646360 | rs7799788 | 7 | 66965701 | 1.503 | 0.0001539 |
| 181 | AX-11562044 | rs628715 | 11 | 94363720 | 0.713 | 0.0001548 |
| 182 | AX-11376226 | rs2222132 | 15 | 46966189 | 2.039 | 0.0001548 |
| 183 | AX-11617257 | rs7246814 | 19 | 33538414 | 0.7682 | 0.0001551 |
| 184 | AX-11627977 | rs7532188 | 1 | 162431320 | 0.6338 | 0.0001556 |
| 185 | AX-11499189 | rs427375 | 2 | 8381906 | 0.7431 | 0.000158 |
| 186 | AX-11475097 | rs36097961 | 19 | 6850767 | 0.6039 | 0.0001595 |
| 187 | AX-11189731 | rs12085938 | 1 | 5595934 | 0.5165 | 0.0001605 |
| 188 | AX-11262086 | rs1422908 | 5 | 174680410 | 0.7371 | 0.0001629 |
| 189 | AX-11471977 | rs35910787 | 5 | 28060810 | 0.7638 | 0.0001644 |
| 190 | AX-11210818 | rs12503428 | 4 | 183688428 | 1.691 | 0.0001663 |
| 191 | AX-11684206 | rs9377506 | 6 | 103962411 | 0.4683 | 0.0001673 |
| 192 | AX-11710498 | rs9924017 | 16 | 25849321 | 1.29 | 0.0001675 |
| 193 | AX-11253002 | rs13407665 | 2 | 231717328 | 0.7043 | 0.0001685 |
| 194 | AX-11507576 | rs4486278 | 9 | 81882552 | 1.316 | 0.0001701 |
| 195 | AX-11440506 | rs34063379 | 5 | 146513079 | 2.319 | 0.0001711 |
| 196 | AX-11511473 | rs4560826 | 8 | 18943192 | 0.7761 | 0.000172 |
| 197 | AX-11233015 | rs12939997 | 17 | 3324720 | 3.312 | 0.0001725 |
| 198 | AX-11537961 | rs4945574 | 6 | 104545642 | 1.487 | 0.0001728 |
| 199 | AX-11713135 | rs9965938 | 18 | 70885038 | 0.6533 | 0.0001732 |
| 200 | AX-11391005 | rs2421059 | 5 | 159022275 | 0.7092 | 0.0001734 |

| **A vs. CD** | | | | | | |
| --- | --- | --- | --- | --- | --- | --- |
| RANK | SNP | RS ID | CHR | BP | OR | P |
| 1 | AX-11333714 | rs17659990 | 3 | 50751149 | 1.925 | 1.35E-07 |
| 2 | AX-11596121 | rs694339 | 18 | 70221980 | 1.973 | 1.41E-07 |
| 3 | AX-11256416 | rs1362126 | 6 | 29691019 | 0.719 | 2.99E-07 |
| 4 | AX-11231931 | rs12916300 | 15 | 28410491 | 1.35 | 3.75E-07 |
| 5 | AX-11231785 | rs12913832 | 15 | 28365618 | 1.343 | 5.12E-07 |
| 6 | AX-11283097 | rs16845107 | 3 | 113127991 | 0.5244 | 5.94E-07 |
| 7 | AX-11546389 | rs5753618 | 22 | 31838518 | 1.366 | 6.17E-07 |
| 8 | AX-11233548 | rs12953717 | 18 | 46453929 | 1.302 | 9.15E-07 |
| 9 | AX-11530011 | rs4836891 | 9 | 125273574 | 1.582 | 9.42E-07 |
| 10 | AX-11633133 | rs7606512 | 2 | 235609674 | 1.837 | 9.46E-07 |
| 11 | AX-11182373 | rs11927424 | 3 | 88757831 | 1.307 | 1.17E-06 |
| 12 | AX-11419097 | rs28541881 | 19 | 34768590 | 1.604 | 1.34E-06 |
| 13 | AX-11285363 | rs16869961 | 4 | 20780315 | 0.7526 | 1.51E-06 |
| 14 | AX-11292018 | rs16944613 | 15 | 91139098 | 1.343 | 1.84E-06 |
| 15 | AX-11429372 | rs2965280 | 19 | 34917700 | 1.606 | 1.85E-06 |
| 16 | AX-11323366 | rs17416314 | 5 | 31804351 | 0.6064 | 2.18E-06 |
| 17 | AX-11122068 | rs10790917 | 11 | 127645687 | 0.7761 | 2.37E-06 |
| 18 | AX-11432042 | rs302359 | 12 | 29950878 | 2.471 | 2.95E-06 |
| 19 | AX-11504018 | rs4397989 | 13 | 85078528 | 0.7571 | 3.18E-06 |
| 20 | AX-11224232 | rs1270639 | 7 | 157455459 | 1.437 | 3.27E-06 |
| 21 | AX-11152099 | rs11224824 | 11 | 101409138 | 1.436 | 3.29E-06 |
| 22 | AX-11210573 | rs12500234 | 4 | 39619870 | 0.7718 | 4.05E-06 |
| 23 | AX-11437554 | rs323473 | 13 | 105359644 | 1.578 | 4.13E-06 |
| 24 | AX-11471365 | rs35874699 | 16 | 26539262 | 0.776 | 4.50E-06 |
| 25 | AX-11487334 | rs3914272 | 3 | 171558536 | 1.38 | 4.73E-06 |
| 26 | AX-11242701 | rs13170463 | 5 | 7983252 | 1.667 | 5.03E-06 |
| 27 | AX-11218989 | rs1262463 | 18 | 52954421 | 1.595 | 5.30E-06 |
| 28 | AX-11186831 | rs1202519 | 1 | 230947140 | 0.7785 | 5.93E-06 |
| 29 | AX-11542255 | rs511674 | 13 | 37422515 | 2.155 | 6.13E-06 |
| 30 | AX-11373349 | rs2184857 | 1 | 240081747 | 0.7731 | 6.24E-06 |
| 31 | AX-11221914 | rs12667108 | 7 | 5133936 | 0.6851 | 6.27E-06 |
| 32 | AX-11176614 | rs11803759 | 1 | 14665131 | 1.851 | 6.37E-06 |
| 33 | AX-11490472 | rs4086865 | 11 | 26012193 | 1.665 | 6.56E-06 |
| 34 | AX-11178730 | rs11853542 | 15 | 88052767 | 1.36 | 6.70E-06 |
| 35 | AX-11235437 | rs12999616 | 2 | 98324381 | 1.338 | 7.40E-06 |
| 36 | AX-11590027 | rs6853410 | 4 | 82693536 | 1.315 | 7.46E-06 |
| 37 | AX-11312547 | rs17177725 | 14 | 37357412 | 0.608 | 7.90E-06 |
| 38 | AX-11676133 | rs926340 | 22 | 31139318 | 1.274 | 8.22E-06 |
| 39 | AX-11540334 | rs4979489 | 9 | 117768680 | 0.7513 | 8.61E-06 |
| 40 | AX-11370821 | rs2152775 | 13 | 105348972 | 1.554 | 8.77E-06 |
| 41 | AX-11700235 | rs9676308 | 19 | 57579077 | 1.643 | 8.94E-06 |
| 42 | AX-11582413 | rs674617 | 18 | 9701782 | 1.486 | 8.98E-06 |
| 43 | AX-11275238 | rs1557614 | 10 | 125247462 | 0.7734 | 9.07E-06 |
| 44 | AX-11158490 | rs11539086 | 3 | 58552329 | 1.522 | 9.46E-06 |
| 45 | AX-11567858 | rs6491880 | 13 | 105346831 | 1.559 | 9.92E-06 |
| 46 | AX-11216902 | rs12593759 | 15 | 91202601 | 1.313 | 1.02E-05 |
| 47 | AX-11666175 | rs8110638 | 19 | 57569719 | 1.616 | 1.06E-05 |
| 48 | AX-11688041 | rs9458376 | 6 | 162219854 | 2.154 | 1.22E-05 |
| 49 | AX-11384077 | rs2306792 | 3 | 113081835 | 0.4125 | 1.32E-05 |
| 50 | AX-11286848 | rs16887197 | 8 | 116246667 | 0.6068 | 1.32E-05 |
| 51 | AX-11418603 | rs28520849 | 4 | 58281562 | 1.54 | 1.34E-05 |
| 52 | AX-11098553 | rs10189164 | 2 | 25633368 | 1.303 | 1.35E-05 |
| 53 | AX-11087719 | rs10019252 | 4 | 40002314 | 0.7681 | 1.43E-05 |
| 54 | AX-11615721 | rs7226855 | 18 | 46454048 | 0.7895 | 1.50E-05 |
| 55 | AX-11151023 | rs11209710 | 1 | 71365018 | 1.277 | 1.50E-05 |
| 56 | AX-11203106 | rs12378118 | 9 | 6033278 | 0.7271 | 1.54E-05 |
| 57 | AX-11635712 | rs7642878 | 3 | 88792115 | 1.263 | 1.57E-05 |
| 58 | AX-11614261 | rs7203386 | 16 | 86972183 | 1.722 | 1.57E-05 |
| 59 | AX-11624322 | rs742223 | 6 | 11705700 | 0.5847 | 1.57E-05 |
| 60 | AX-11369864 | rs2139727 | 12 | 119083418 | 1.74 | 1.60E-05 |
| 61 | AX-11380867 | rs2277887 | 2 | 56145541 | 0.6267 | 1.64E-05 |
| 62 | AX-11193622 | rs12154234 | 7 | 123600607 | 1.267 | 1.70E-05 |
| 63 | AX-11213027 | rs12533277 | 7 | 96777930 | 1.694 | 1.74E-05 |
| 64 | AX-11589348 | rs6842825 | 4 | 57125176 | 2.311 | 1.74E-05 |
| 65 | AX-11408568 | rs2742345 | 2 | 179568682 | 1.683 | 1.80E-05 |
| 66 | AX-11509711 | rs4542868 | 2 | 161485791 | 1.476 | 1.83E-05 |
| 67 | AX-11564733 | rs6447984 | 4 | 20833134 | 0.7468 | 1.84E-05 |
| 68 | AX-11421506 | rs28655514 | 14 | 62493964 | 1.56 | 1.85E-05 |
| 69 | AX-11232333 | rs12924567 | 16 | 26539214 | 1.261 | 1.85E-05 |
| 70 | AX-11580488 | rs6718138 | 2 | 161555610 | 1.308 | 1.98E-05 |
| 71 | AX-11310150 | rs17146516 | 7 | 123597121 | 0.7237 | 2.00E-05 |
| 72 | AX-11274230 | rs1547553 | 18 | 77926197 | 1.365 | 2.09E-05 |
| 73 | AX-11530892 | rs4849957 | 2 | 4288725 | 1.258 | 2.09E-05 |
| 74 | AX-11356568 | rs1967855 | 14 | 90009698 | 1.354 | 2.11E-05 |
| 75 | AX-11661293 | rs8032475 | 15 | 92437798 | 1.348 | 2.17E-05 |
| 76 | AX-11620272 | rs7308665 | 12 | 102006501 | 0.7656 | 2.24E-05 |
| 77 | AX-11280889 | rs1672713 | 11 | 113955678 | 1.321 | 2.24E-05 |
| 78 | AX-11631376 | rs7581703 | 2 | 221913924 | 1.401 | 2.26E-05 |
| 79 | AX-11163429 | rs11625750 | 14 | 62391885 | 1.27 | 2.38E-05 |
| 80 | AX-11642476 | rs7742915 | 6 | 38147745 | 1.283 | 2.46E-05 |
| 81 | AX-11194751 | rs12190458 | 6 | 106875317 | 0.7065 | 2.46E-05 |
| 82 | AX-11315696 | rs17239917 | 15 | 90333851 | 0.7285 | 2.54E-05 |
| 83 | AX-11313212 | rs17189981 | 2 | 54666673 | 0.7361 | 2.54E-05 |
| 84 | AX-11380285 | rs2272613 | 8 | 1873540 | 1.516 | 2.58E-05 |
| 85 | AX-11310942 | rs17155568 | 10 | 14644455 | 1.829 | 2.58E-05 |
| 86 | AX-11213697 | rs12542761 | 8 | 72375596 | 1.685 | 2.60E-05 |
| 87 | AX-11224121 | rs1270449 | 10 | 30216662 | 1.293 | 2.66E-05 |
| 88 | AX-11318743 | rs17308449 | 10 | 6865955 | 1.773 | 2.76E-05 |
| 89 | AX-11567190 | rs6482354 | 10 | 24296524 | 2.566 | 2.78E-05 |
| 90 | AX-11674678 | rs917543 | 16 | 7743747 | 0.7835 | 2.81E-05 |
| 91 | AX-11137772 | rs11010633 | 10 | 36637021 | 1.281 | 2.84E-05 |
| 92 | AX-11566738 | rs6475831 | 9 | 25004901 | 0.7975 | 2.89E-05 |
| 93 | AX-11705993 | rs9850050 | 3 | 58476832 | 1.268 | 2.94E-05 |
| 94 | AX-11631749 | rs7587054 | 2 | 81171525 | 1.419 | 3.17E-05 |
| 95 | AX-11495551 | rs41429646 | 3 | 181255017 | 1.662 | 3.18E-05 |
| 96 | AX-11274913 | rs1554395 | 3 | 183664873 | 0.7652 | 3.23E-05 |
| 97 | AX-11173142 | rs11756830 | 6 | 38148413 | 1.254 | 3.31E-05 |
| 98 | AX-11686596 | rs941543 | 14 | 100014438 | 0.8016 | 3.39E-05 |
| 99 | AX-11538691 | rs4954585 | 2 | 136998394 | 1.258 | 3.40E-05 |
| 100 | AX-11345432 | rs1816922 | 2 | 176745836 | 1.25 | 3.41E-05 |
| 101 | AX-11426467 | rs2908838 | 12 | 11638238 | 1.476 | 3.44E-05 |
| 102 | AX-11096277 | rs10152168 | 15 | 92429670 | 1.274 | 3.46E-05 |
| 103 | AX-11611381 | rs7165476 | 15 | 91151948 | 1.276 | 3.47E-05 |
| 104 | AX-11294565 | rs16971113 | 18 | 36487508 | 1.321 | 3.49E-05 |
| 105 | AX-11217657 | rs12604637 | 18 | 8556716 | 1.377 | 3.67E-05 |
| 106 | AX-11583939 | rs6767291 | 3 | 58408910 | 1.311 | 3.70E-05 |
| 107 | AX-11280720 | rs1667394 | 15 | 28530182 | 1.32 | 3.73E-05 |
| 108 | AX-11564796 | rs6448887 | 4 | 12361202 | 1.249 | 3.78E-05 |
| 109 | AX-11636802 | rs765744 | 22 | 23324839 | 0.798 | 3.80E-05 |
| 110 | AX-11646401 | rs7800235 | 7 | 25135566 | 1.297 | 3.89E-05 |
| 111 | AX-11375405 | rs221170 | 7 | 29005441 | 1.311 | 3.93E-05 |
| 112 | AX-11495330 | rs41412849 | 14 | 94943281 | 1.709 | 3.96E-05 |
| 113 | AX-11513062 | rs4599370 | 4 | 169641509 | 1.29 | 3.96E-05 |
| 114 | AX-11169566 | rs11710823 | 3 | 58396493 | 1.31 | 3.98E-05 |
| 115 | AX-11209521 | rs12485999 | 3 | 58409763 | 1.31 | 4.04E-05 |
| 116 | AX-11387116 | rs2361095 | 9 | 634862 | 1.412 | 4.04E-05 |
| 117 | AX-11660773 | rs8025035 | 15 | 28377772 | 1.447 | 4.06E-05 |
| 118 | AX-11266725 | rs1472081 | 17 | 27395890 | 1.269 | 4.09E-05 |
| 119 | AX-11139144 | rs11032678 | 11 | 34438320 | 1.273 | 4.12E-05 |
| 120 | AX-11541629 | rs5028596 | 11 | 34438623 | 1.273 | 4.12E-05 |
| 121 | AX-11615364 | rs7221059 | 17 | 74988444 | 0.7403 | 4.13E-05 |
| 122 | AX-11239896 | rs13109321 | 4 | 37965492 | 1.394 | 4.15E-05 |
| 123 | AX-11537438 | rs4939827 | 18 | 46453463 | 0.8009 | 4.16E-05 |
| 124 | AX-11573149 | rs6575650 | 14 | 98754419 | 1.643 | 4.30E-05 |
| 125 | AX-11557802 | rs606826 | 18 | 70197285 | 1.55 | 4.33E-05 |
| 126 | AX-11162349 | rs11611201 | 12 | 13442314 | 0.787 | 4.35E-05 |
| 127 | AX-11546267 | rs5750616 | 22 | 38918894 | 0.7676 | 4.39E-05 |
| 128 | AX-11474994 | rs36092369 | 19 | 55698955 | 2.123 | 4.39E-05 |
| 129 | AX-11219629 | rs12635366 | 3 | 58398466 | 1.308 | 4.41E-05 |
| 130 | AX-11704149 | rs9822328 | 3 | 58407837 | 1.308 | 4.41E-05 |
| 131 | AX-11279720 | rs1635166 | 15 | 28539834 | 1.334 | 4.41E-05 |
| 132 | AX-11513655 | rs4613423 | 3 | 58341568 | 1.307 | 4.50E-05 |
| 133 | AX-11379207 | rs2255094 | 2 | 46232042 | 0.7942 | 4.52E-05 |
| 134 | AX-11607990 | rs7118878 | 11 | 26017508 | 1.579 | 4.54E-05 |
| 135 | AX-11713856 | rs9979119 | 21 | 20835175 | 1.774 | 4.55E-05 |
| 136 | AX-11503736 | rs4390943 | 3 | 58376639 | 1.308 | 4.55E-05 |
| 137 | AX-11634517 | rs7625947 | 3 | 58483630 | 1.257 | 4.59E-05 |
| 138 | AX-11608817 | rs7131401 | 11 | 79534897 | 0.7447 | 4.59E-05 |
| 139 | AX-11209746 | rs12489103 | 3 | 58373167 | 1.307 | 4.62E-05 |
| 140 | AX-11260378 | rs1404228 | 7 | 113050788 | 1.445 | 4.77E-05 |
| 141 | AX-11365284 | rs2070662 | 3 | 58415590 | 1.306 | 4.77E-05 |
| 142 | AX-11304155 | rs17078788 | 13 | 24020177 | 1.703 | 4.81E-05 |
| 143 | AX-11184746 | rs11974484 | 7 | 123584387 | 1.252 | 4.87E-05 |
| 144 | AX-11538243 | rs4949330 | 1 | 31449766 | 1.818 | 4.94E-05 |
| 145 | AX-11584022 | rs6768547 | 3 | 58434969 | 1.307 | 4.95E-05 |
| 146 | AX-11391402 | rs2426054 | 20 | 46567681 | 1.372 | 5.01E-05 |
| 147 | AX-11691659 | rs9520422 | 13 | 108034541 | 1.247 | 5.08E-05 |
| 148 | AX-11674354 | rs913294 | 10 | 14776029 | 1.244 | 5.11E-05 |
| 149 | AX-11340077 | rs17782474 | 2 | 100265936 | 1.41 | 5.12E-05 |
| 150 | AX-11502913 | rs4369418 | 11 | 132905369 | 0.799 | 5.25E-05 |
| 151 | AX-11609081 | rs7134911 | 12 | 30975469 | 0.7988 | 5.25E-05 |
| 152 | AX-11319713 | rs17327666 | 4 | 183613512 | 0.7563 | 5.26E-05 |
| 153 | AX-11283201 | rs16846234 | 2 | 162857452 | 1.584 | 5.32E-05 |
| 154 | AX-11694740 | rs956089 | 7 | 52611144 | 1.412 | 5.34E-05 |
| 155 | AX-11581656 | rs673439 | 18 | 41772564 | 0.7574 | 5.44E-05 |
| 156 | AX-11274408 | rs154929 | 18 | 58817565 | 0.7515 | 5.45E-05 |
| 157 | AX-11396358 | rs2517913 | 6 | 29683542 | 0.7423 | 5.46E-05 |
| 158 | AX-11423734 | rs75972823 | 3 | 121334527 | 0.7458 | 5.48E-05 |
| 159 | AX-11589381 | rs6843352 | 4 | 157060216 | 1.397 | 5.48E-05 |
| 160 | AX-11401697 | rs2619139 | 12 | 101932976 | 1.28 | 5.58E-05 |
| 161 | AX-11319064 | rs17314998 | 3 | 99785894 | 2.052 | 5.59E-05 |
| 162 | AX-11522097 | rs4737320 | 8 | 72415990 | 1.714 | 5.63E-05 |
| 163 | AX-11562997 | rs6419880 | 3 | 88756407 | 1.242 | 5.73E-05 |
| 164 | AX-11582814 | rs75440621 | 2 | 5958445 | 1.279 | 5.74E-05 |
| 165 | AX-11091657 | rs10084463 | 2 | 46219119 | 1.241 | 5.77E-05 |
| 166 | AX-11532985 | rs4876802 | 8 | 119222182 | 0.7799 | 5.78E-05 |
| 167 | AX-11155235 | N/A | 1 | 160061384 | 1.334 | 5.79E-05 |
| 168 | AX-11203839 | rs12406176 | 1 | 221678052 | 0.6296 | 5.83E-05 |
| 169 | AX-11208023 | rs12464563 | 2 | 46205320 | 0.8032 | 5.89E-05 |
| 170 | AX-11269926 | rs1505521 | 8 | 118076115 | 1.617 | 5.94E-05 |
| 171 | AX-11675388 | rs924873 | 5 | 168247565 | 1.24 | 6.02E-05 |
| 172 | AX-11545956 | rs5743291 | 16 | 50757276 | 1.406 | 6.02E-05 |
| 173 | AX-11650513 | rs7860446 | 9 | 125332930 | 1.404 | 6.04E-05 |
| 174 | AX-11653952 | rs7922424 | 10 | 133316452 | 1.521 | 6.07E-05 |
| 175 | AX-11617915 | rs7259160 | 19 | 34959979 | 1.359 | 6.17E-05 |
| 176 | AX-11619933 | rs7303284 | 12 | 104707546 | 1.685 | 6.24E-05 |
| 177 | AX-11358149 | rs1991695 | 8 | 124740499 | 0.7998 | 6.27E-05 |
| 178 | AX-11101428 | rs10236910 | 7 | 86530108 | 1.379 | 6.29E-05 |
| 179 | AX-11318058 | rs1729173 | 8 | 13224834 | 0.7853 | 6.41E-05 |
| 180 | AX-11329253 | rs17567255 | 7 | 66413334 | 1.677 | 6.41E-05 |
| 181 | AX-11429954 | rs2974658 | 5 | 2960267 | 0.8018 | 6.47E-05 |
| 182 | AX-11592971 | rs6899148 | 5 | 180205940 | 1.467 | 6.66E-05 |
| 183 | AX-11375413 | rs2211792 | 21 | 35471815 | 0.798 | 6.67E-05 |
| 184 | AX-11580428 | rs6717445 | 2 | 221935421 | 2.116 | 6.79E-05 |
| 185 | AX-11640072 | rs770569 | 11 | 99882216 | 1.367 | 6.87E-05 |
| 186 | AX-11562550 | rs637041 | 10 | 6236409 | 1.252 | 6.88E-05 |
| 187 | AX-11490084 | rs4075152 | 3 | 58385194 | 1.3 | 6.91E-05 |
| 188 | AX-11406922 | rs2717886 | 7 | 25174697 | 1.287 | 6.91E-05 |
| 189 | AX-11699878 | rs9664487 | 10 | 91038452 | 1.299 | 6.97E-05 |
| 190 | AX-11655589 | rs7950262 | 11 | 25671657 | 1.243 | 6.99E-05 |
| 191 | AX-11355590 | rs1953774 | 14 | 86443312 | 1.305 | 7.00E-05 |
| 192 | AX-11584315 | rs6772652 | 3 | 58406693 | 1.298 | 7.14E-05 |
| 193 | AX-11712523 | rs9956879 | 18 | 57715633 | 1.236 | 7.31E-05 |
| 194 | AX-11655161 | rs7942260 | 11 | 94345502 | 0.6761 | 7.32E-05 |
| 195 | AX-11150775 | rs11206733 | 1 | 56541706 | 1.292 | 7.39E-05 |
| 196 | AX-11697687 | rs961067 | 4 | 2505606 | 1.283 | 7.39E-05 |
| 197 | AX-11312454 | rs17175983 | 17 | 3938972 | 1.973 | 7.44E-05 |
| 198 | AX-11384966 | rs2325621 | 13 | 74929721 | 1.777 | 7.52E-05 |
| 199 | AX-11674615 | rs916977 | 15 | 28513364 | 1.311 | 7.55E-05 |
| 200 | AX-11667451 | rs8177061 | 10 | 30745304 | 1.886 | 7.69E-05 |

| **AB vs. C** | | | | | | |
| --- | --- | --- | --- | --- | --- | --- |
| RANK | SNP | RS ID | CHR | BP | OR | P |
| 1 | AX-11352279 | rs1912804 | 16 | 78626583 | 1.586 | 1.17E-06 |
| 2 | AX-11577254 | rs6668441 | 1 | 102242917 | 0.5191 | 4.51E-06 |
| 3 | AX-11465091 | rs35505947 | 18 | 13954109 | 0.6846 | 5.58E-06 |
| 4 | AX-11312645 | rs17179503 | 7 | 81987289 | 0.5432 | 6.22E-06 |
| 5 | AX-11322058 | rs17382698 | 8 | 129139206 | 1.522 | 8.85E-06 |
| 6 | AX-11159130 | rs1155750 | 6 | 54730344 | 0.6976 | 9.28E-06 |
| 7 | AX-11150713 | rs11205451 | 1 | 48647860 | 2.735 | 1.10E-05 |
| 8 | AX-11349269 | rs1877720 | 1 | 48824407 | 2.328 | 1.19E-05 |
| 9 | AX-11423669 | rs2876841 | 7 | 39537647 | 0.6482 | 1.23E-05 |
| 10 | AX-11702579 | rs979006 | 4 | 54452878 | 1.499 | 1.30E-05 |
| 11 | AX-11276757 | rs1571648 | 9 | 27332772 | 0.7208 | 1.38E-05 |
| 12 | AX-11165981 | rs11659657 | 18 | 13989970 | 0.6973 | 1.62E-05 |
| 13 | AX-11342292 | rs1782633 | 6 | 161249326 | 1.511 | 1.64E-05 |
| 14 | AX-11547916 | rs582962 | 6 | 69521978 | 1.319 | 1.65E-05 |
| 15 | AX-11645152 | rs7781557 | 7 | 102481891 | 0.7356 | 1.70E-05 |
| 16 | AX-11669725 | N/A | 7 | 147389581 | 0.692 | 1.77E-05 |
| 17 | AX-11418828 | rs28531799 | 9 | 79575623 | 1.778 | 1.78E-05 |
| 18 | AX-11109554 | rs10485879 | 7 | 77905130 | 1.41 | 1.81E-05 |
| 19 | AX-11331925 | rs17626293 | 1 | 195651519 | 0.5536 | 2.06E-05 |
| 20 | AX-11332564 | rs1763894 | 10 | 61286319 | 1.304 | 2.32E-05 |
| 21 | AX-11375683 | rs2215040 | 7 | 102496594 | 0.7555 | 2.36E-05 |
| 22 | AX-11514909 | rs4644524 | 1 | 199945630 | 0.7687 | 2.51E-05 |
| 23 | AX-11137764 | rs1101047 | 7 | 147392446 | 0.73 | 2.55E-05 |
| 24 | AX-11269926 | rs1505521 | 8 | 118076115 | 2.061 | 2.57E-05 |
| 25 | AX-11369564 | rs2135159 | 2 | 213214465 | 1.459 | 2.59E-05 |
| 26 | AX-11170793 | rs11726114 | 4 | 60010352 | 0.6586 | 2.62E-05 |
| 27 | AX-11133204 | rs10941252 | 5 | 35643615 | 1.302 | 2.75E-05 |
| 28 | AX-11364453 | rs2062961 | 2 | 117077958 | 0.7751 | 2.81E-05 |
| 29 | AX-11133972 | rs10952705 | 7 | 147417555 | 0.7652 | 2.87E-05 |
| 30 | AX-11295241 | rs16978467 | 18 | 43305518 | 0.548 | 2.88E-05 |
| 31 | AX-11609081 | rs7134911 | 12 | 30975469 | 0.774 | 2.98E-05 |
| 32 | AX-11274913 | rs1554395 | 3 | 183664873 | 0.7523 | 3.02E-05 |
| 33 | AX-11121255 | rs10780947 | 9 | 73165078 | 0.7666 | 3.19E-05 |
| 34 | AX-11128916 | rs10878977 | 12 | 69799690 | 1.333 | 3.58E-05 |
| 35 | AX-11111213 | rs10495672 | 2 | 18494455 | 1.342 | 3.66E-05 |
| 36 | AX-11480877 | rs3790843 | 1 | 200010824 | 1.318 | 4.03E-05 |
| 37 | AX-11348587 | rs1870944 | 12 | 89645588 | 0.3424 | 4.15E-05 |
| 38 | AX-11669320 | rs845868 | 6 | 998813 | 0.7464 | 4.18E-05 |
| 39 | AX-11145214 | rs11123333 | 2 | 116909251 | 0.7785 | 4.27E-05 |
| 40 | AX-11145388 | rs11125447 | 2 | 53055130 | 1.413 | 4.56E-05 |
| 41 | AX-11394655 | rs2489944 | 6 | 161292913 | 1.436 | 4.56E-05 |
| 42 | AX-11206664 | rs12444232 | 16 | 20321467 | 0.5706 | 4.91E-05 |
| 43 | AX-11530867 | rs4849449 | 2 | 116955767 | 0.7809 | 5.08E-05 |
| 44 | AX-11199103 | rs12285517 | 11 | 98195448 | 1.926 | 5.42E-05 |
| 45 | AX-11286766 | rs16886105 | 6 | 54735344 | 0.6684 | 5.54E-05 |
| 46 | AX-11302642 | rs17064859 | 18 | 56020793 | 0.632 | 5.55E-05 |
| 47 | AX-11187081 | rs12029406 | 1 | 199905828 | 1.284 | 5.56E-05 |
| 48 | AX-11354464 | rs1941098 | 18 | 13988795 | 0.7388 | 5.95E-05 |
| 49 | AX-11269952 | rs1505758 | 2 | 117078901 | 0.7833 | 6.27E-05 |
| 50 | AX-11288942 | rs16909124 | 12 | 13617949 | 0.5712 | 6.39E-05 |
| 51 | AX-11315256 | rs17230787 | 10 | 33708150 | 1.428 | 6.40E-05 |
| 52 | AX-11276363 | N/A | 12 | 71074730 | 1.454 | 6.46E-05 |
| 53 | AX-11421506 | rs28655514 | 14 | 62493964 | 1.699 | 6.53E-05 |
| 54 | AX-11127370 | rs10859378 | 12 | 93139234 | 0.5661 | 6.74E-05 |
| 55 | AX-11141123 | rs11062055 | 12 | 2107301 | 0.7206 | 7.04E-05 |
| 56 | AX-11168685 | rs11697255 | 20 | 52037408 | 0.6481 | 7.07E-05 |
| 57 | AX-11280329 | rs1654668 | 19 | 55174213 | 0.783 | 7.14E-05 |
| 58 | AX-11476083 | rs366676 | 6 | 88717766 | 0.7871 | 7.24E-05 |
| 59 | AX-11633012 | rs7604753 | 2 | 117135650 | 0.7808 | 7.32E-05 |
| 60 | AX-11633133 | rs7606512 | 2 | 235609674 | 2.008 | 7.33E-05 |
| 61 | AX-11535782 | rs4915390 | 1 | 199824908 | 1.282 | 7.37E-05 |
| 62 | AX-11102787 | rs10261983 | 7 | 37885121 | 0.7771 | 7.64E-05 |
| 63 | AX-11471977 | rs35910787 | 5 | 28060810 | 0.7765 | 7.66E-05 |
| 64 | AX-11232873 | rs12936143 | 17 | 2556971 | 1.318 | 7.74E-05 |
| 65 | AX-11280619 | rs166354 | 5 | 169330699 | 1.299 | 8.02E-05 |
| 66 | AX-11648584 | rs7831492 | 8 | 41614503 | 0.7827 | 8.06E-05 |
| 67 | AX-11578799 | rs6693264 | 1 | 48556937 | 2.474 | 8.09E-05 |
| 68 | AX-11087310 | rs10012525 | 4 | 16912717 | 0.614 | 8.18E-05 |
| 69 | AX-11512086 | rs4573161 | 7 | 46888951 | 0.765 | 8.31E-05 |
| 70 | AX-11187942 | rs12044374 | 1 | 94635986 | 1.272 | 8.35E-05 |
| 71 | AX-11596952 | rs6955613 | 7 | 133737365 | 1.283 | 8.85E-05 |
| 72 | AX-11285363 | rs16869961 | 4 | 20780315 | 0.78 | 8.86E-05 |
| 73 | AX-11439110 | rs33962189 | 4 | 157266122 | 1.281 | 8.95E-05 |
| 74 | AX-11380410 | rs2273765 | 9 | 77697526 | 0.6246 | 9.07E-05 |
| 75 | AX-11710498 | rs9924017 | 16 | 25849321 | 1.27 | 9.55E-05 |
| 76 | AX-11665892 | rs8103929 | 19 | 40279758 | 1.292 | 9.60E-05 |
| 77 | AX-11475052 | rs36095535 | 2 | 12289869 | 1.44 | 9.68E-05 |
| 78 | AX-11696145 | rs9583269 | 13 | 109283030 | 0.7757 | 9.68E-05 |
| 79 | AX-11485457 | rs3858320 | 10 | 127978792 | 1.277 | 9.95E-05 |
| 80 | AX-11092434 | rs10095646 | 8 | 119065253 | 0.7886 | 0.0001015 |
| 81 | AX-11557932 | rs6070316 | 20 | 56517205 | 0.5646 | 0.0001031 |
| 82 | AX-11619933 | rs7303284 | 12 | 104707546 | 1.941 | 0.0001039 |
| 83 | AX-11647376 | rs7813805 | 8 | 119048760 | 0.7881 | 0.0001056 |
| 84 | AX-11333714 | rs17659990 | 3 | 50751149 | 1.82 | 0.0001061 |
| 85 | AX-11627977 | rs7532188 | 1 | 162431320 | 0.6636 | 0.0001101 |
| 86 | AX-11093040 | rs10104828 | 8 | 72701830 | 1.304 | 0.0001137 |
| 87 | AX-11148067 | rs11161729 | 1 | 86551864 | 0.792 | 0.0001159 |
| 88 | AX-11590149 | rs6854985 | 4 | 68059850 | 1.339 | 0.0001174 |
| 89 | AX-11212471 | rs12526148 | 6 | 161967901 | 1.297 | 0.0001185 |
| 90 | AX-11199505 | rs12296006 | 11 | 98229808 | 1.57 | 0.0001186 |
| 91 | AX-11649587 | rs7846043 | 8 | 13412918 | 0.7854 | 0.000119 |
| 92 | AX-11300557 | rs1704299 | 18 | 68468584 | 0.5394 | 0.0001231 |
| 93 | AX-11150524 | rs11202641 | 10 | 89818871 | 0.7695 | 0.0001235 |
| 94 | AX-11137772 | rs11010633 | 10 | 36637021 | 1.3 | 0.0001237 |
| 95 | AX-11341249 | rs17805809 | 10 | 117894402 | 1.447 | 0.0001241 |
| 96 | AX-11200110 | rs12311795 | 12 | 23051623 | 0.6872 | 0.0001253 |
| 97 | AX-11640967 | rs7719458 | 5 | 35703042 | 1.268 | 0.0001256 |
| 98 | AX-11264214 | rs1444867 | 3 | 55396479 | 1.834 | 0.0001268 |
| 99 | AX-11334896 | rs17682748 | 6 | 6571187 | 0.5599 | 0.0001269 |
| 100 | AX-11395729 | rs250956 | 2 | 234993563 | 1.347 | 0.0001301 |
| 101 | AX-11301710 | rs17055742 | 13 | 38040751 | 0.4369 | 0.0001308 |
| 102 | AX-11130670 | rs10903747 | 10 | 2380746 | 0.7242 | 0.0001311 |
| 103 | AX-11095544 | rs10141419 | 14 | 89454718 | 0.4496 | 0.0001333 |
| 104 | AX-11124334 | rs10818024 | 9 | 120108044 | 1.284 | 0.0001341 |
| 105 | AX-11243153 | rs13180075 | 5 | 28079752 | 0.7908 | 0.0001342 |
| 106 | AX-11699557 | rs9655053 | 7 | 46897107 | 0.7711 | 0.0001347 |
| 107 | AX-11306651 | rs17106529 | 12 | 69804612 | 1.452 | 0.0001398 |
| 108 | AX-11420404 | rs2860290 | 11 | 129291462 | 1.285 | 0.0001409 |
| 109 | AX-11405595 | rs2698717 | 7 | 26403801 | 1.256 | 0.0001459 |
| 110 | AX-11231906 | rs12915935 | 15 | 100412080 | 1.288 | 0.0001463 |
| 111 | AX-11657883 | rs798300 | 7 | 77953248 | 1.338 | 0.0001472 |
| 112 | AX-11261780 | rs1420161 | 7 | 96988034 | 1.285 | 0.00015 |
| 113 | AX-11432826 | rs3087967 | 11 | 111156836 | 1.306 | 0.0001518 |
| 114 | AX-11481813 | rs3802842 | 11 | 111171709 | 1.306 | 0.0001518 |
| 115 | AX-11307008 | rs17110136 | 14 | 40447949 | 0.6554 | 0.0001543 |
| 116 | AX-11299225 | rs17028348 | 2 | 64263931 | 0.3991 | 0.0001547 |
| 117 | AX-11123010 | rs10802909 | 1 | 240985581 | 0.7739 | 0.0001583 |
| 118 | AX-11124326 | rs10817971 | 9 | 119766782 | 1.312 | 0.0001591 |
| 119 | AX-11595883 | rs6939914 | 6 | 145573729 | 0.7856 | 0.0001604 |
| 120 | AX-11513809 | rs4617472 | 10 | 66758618 | 1.267 | 0.0001611 |
| 121 | AX-11365290 | rs2070715 | 5 | 142024256 | 0.7948 | 0.0001629 |
| 122 | AX-11642108 | rs7737567 | 5 | 88184591 | 0.5121 | 0.0001643 |
| 123 | AX-11320385 | rs17342699 | 1 | 34339953 | 2.377 | 0.0001644 |
| 124 | AX-11642494 | rs7743188 | 6 | 54773639 | 0.709 | 0.0001644 |
| 125 | AX-11102292 | rs10252449 | 7 | 96981468 | 1.338 | 0.0001674 |
| 126 | AX-11708945 | rs9896061 | 17 | 11694246 | 0.433 | 0.0001701 |
| 127 | AX-11583374 | rs6760000 | 2 | 42337820 | 0.7899 | 0.0001712 |
| 128 | AX-11641788 | rs7732695 | 5 | 112495421 | 0.6116 | 0.000176 |
| 129 | AX-11369194 | rs2129954 | 11 | 42098693 | 0.64 | 0.0001763 |
| 130 | AX-11240830 | rs13129679 | 4 | 2576102 | 2.805 | 0.0001767 |
| 131 | AX-11127442 | rs10860021 | 12 | 96747710 | 1.275 | 0.0001776 |
| 132 | AX-11467387 | rs35637782 | 4 | 135640298 | 1.448 | 0.0001785 |
| 133 | AX-11174174 | rs11770345 | 7 | 24483969 | 0.791 | 0.0001789 |
| 134 | AX-11380285 | rs2272613 | 8 | 1873540 | 1.581 | 0.0001795 |
| 135 | AX-11686288 | rs9405753 | 6 | 4611829 | 0.796 | 0.0001805 |
| 136 | AX-11310150 | rs17146516 | 7 | 123597121 | 0.7418 | 0.0001819 |
| 137 | AX-11513427 | rs111388405 | 11 | 111172179 | 1.302 | 0.0001838 |
| 138 | AX-11691173 | rs9514535 | 13 | 107088897 | 0.7985 | 0.0001861 |
| 139 | AX-11293133 | rs16955607 | 18 | 50354434 | 0.6429 | 0.0001869 |
| 140 | AX-11119496 | rs10759915 | 9 | 120068606 | 1.257 | 0.0001875 |
| 141 | AX-11546699 | rs5761119 | 22 | 25953851 | 1.895 | 0.0001882 |
| 142 | AX-11544907 | rs557107 | 11 | 128645449 | 0.763 | 0.0001892 |
| 143 | AX-11235911 | rs13010690 | 2 | 217642427 | 0.7385 | 0.0001908 |
| 144 | AX-11516168 | rs4663979 | 2 | 234803432 | 0.7871 | 0.0001934 |
| 145 | AX-11657345 | rs7975356 | 12 | 29630081 | 0.7263 | 0.0001947 |
| 146 | AX-11595761 | rs6937873 | 6 | 998948 | 0.7856 | 0.0001956 |
| 147 | AX-11451549 | N/A | 3 | 725917 | 0.5092 | 0.0002018 |
| 148 | AX-11087719 | rs10019252 | 4 | 40002314 | 0.7803 | 0.0002028 |
| 149 | AX-11407577 | rs2726595 | 8 | 59710250 | 1.342 | 0.0002043 |
| 150 | AX-11265039 | rs1453439 | 11 | 5842628 | 0.5965 | 0.0002046 |
| 151 | AX-11193555 | rs12153246 | 5 | 90421498 | 0.7768 | 0.0002066 |
| 152 | AX-11255120 | rs1348964 | 11 | 45663536 | 0.794 | 0.0002105 |
| 153 | AX-11260530 | rs1406084 | 2 | 154688728 | 0.7561 | 0.0002117 |
| 154 | AX-11212264 | rs12523586 | 5 | 28747075 | 1.515 | 0.0002133 |
| 155 | AX-11086712 | rs10002854 | 4 | 7521146 | 0.528 | 0.000214 |
| 156 | AX-11693976 | rs954980 | 9 | 3761909 | 0.789 | 0.000214 |
| 157 | AX-11086758 | rs10003692 | 4 | 60124780 | 0.7273 | 0.0002142 |
| 158 | AX-11567409 | rs6485905 | 11 | 12602389 | 0.7688 | 0.0002153 |
| 159 | AX-11407212 | rs2722355 | 7 | 37897209 | 0.7708 | 0.0002166 |
| 160 | AX-11251819 | rs1338316 | 1 | 48961629 | 2.221 | 0.0002184 |
| 161 | AX-11339196 | rs17765780 | 3 | 55934415 | 1.447 | 0.0002214 |
| 162 | AX-11262086 | rs1422908 | 5 | 174680410 | 0.7685 | 0.0002214 |
| 163 | AX-11175266 | rs11783389 | 8 | 123168870 | 0.6363 | 0.0002219 |
| 164 | AX-11299922 | rs17035611 | 3 | 1253821 | 0.6247 | 0.000223 |
| 165 | AX-11324834 | rs17453291 | 8 | 5525983 | 0.6578 | 0.0002251 |
| 166 | AX-11273510 | rs1541282 | 18 | 13992614 | 0.751 | 0.0002284 |
| 167 | AX-11419403 | rs2855798 | 11 | 128732961 | 1.349 | 0.0002285 |
| 168 | AX-11469776 | rs357818 | 4 | 59995557 | 0.7232 | 0.0002293 |
| 169 | AX-11631811 | rs7588002 | 2 | 117200339 | 0.7989 | 0.0002303 |
| 170 | AX-11397314 | rs253482 | 5 | 16897446 | 0.7991 | 0.0002303 |
| 171 | AX-11378193 | rs2244884 | 21 | 19709819 | 1.311 | 0.0002322 |
| 172 | AX-11169308 | rs11707642 | 3 | 193820138 | 0.7102 | 0.0002336 |
| 173 | AX-11680168 | rs9313333 | 5 | 166038872 | 3.199 | 0.0002337 |
| 174 | AX-11309032 | rs17133451 | 7 | 138742981 | 0.7346 | 0.0002339 |
| 175 | AX-11207600 | rs12457067 | 18 | 75670461 | 0.7997 | 0.0002378 |
| 176 | AX-11480878 | rs3790845 | 1 | 200006861 | 1.294 | 0.0002384 |
| 177 | AX-11378767 | rs2250723 | 9 | 90831398 | 0.7601 | 0.0002388 |
| 178 | AX-11426967 | rs2918101 | 10 | 132984326 | 0.7936 | 0.0002393 |
| 179 | AX-11713749 | rs9977229 | 21 | 19689010 | 1.345 | 0.0002398 |
| 180 | AX-11352408 | rs1914837 | 4 | 166480208 | 0.7777 | 0.0002437 |
| 181 | AX-11689478 | rs9484015 | 6 | 137629936 | 0.7312 | 0.0002458 |
| 182 | AX-11641181 | rs7722488 | 5 | 116268205 | 0.723 | 0.0002461 |
| 183 | AX-11387116 | rs2361095 | 9 | 634862 | 1.472 | 0.0002486 |
| 184 | AX-11489822 | rs40719 | 5 | 9171202 | 0.8005 | 0.0002501 |
| 185 | AX-11230274 | rs12883250 | 14 | 88446876 | 1.258 | 0.000251 |
| 186 | AX-11696637 | rs9592121 | 13 | 62138319 | 1.58 | 0.0002518 |
| 187 | AX-11698775 | rs9641113 | 7 | 92967663 | 0.8001 | 0.0002548 |
| 188 | AX-11484309 | rs3843987 | 6 | 137646068 | 0.7481 | 0.0002552 |
| 189 | AX-11629793 | rs7559911 | 2 | 213186351 | 1.815 | 0.0002576 |
| 190 | AX-11258857 | rs1388011 | 4 | 157319984 | 1.286 | 0.0002584 |
| 191 | AX-11434345 | rs3115243 | 2 | 34983529 | 1.399 | 0.000259 |
| 192 | AX-11300799 | rs17045646 | 4 | 114050591 | 0.7452 | 0.0002592 |
| 193 | AX-11177095 | rs11815242 | 10 | 35995340 | 0.6809 | 0.0002593 |
| 194 | AX-11597356 | rs6961571 | 7 | 6517700 | 0.7404 | 0.0002596 |
| 195 | AX-11119475 | rs10759766 | 9 | 118067925 | 1.253 | 0.00026 |
| 196 | AX-11150748 | rs11206321 | 1 | 54762317 | 0.797 | 0.0002601 |
| 197 | AX-11287981 | rs16899063 | 8 | 102176188 | 2.937 | 0.0002605 |
| 198 | AX-11104529 | rs1032608 | 1 | 167131321 | 1.384 | 0.0002618 |
| 199 | AX-11711223 | rs9935276 | 16 | 24242392 | 1.412 | 0.000263 |
| 200 | AX-11421741 | rs2866705 | 20 | 40330362 | 1.247 | 0.0002648 |

| **AB vs. CD** | | | | | | |
| --- | --- | --- | --- | --- | --- | --- |
| RANK | SNP | RS ID | CHR | BP | OR | P |
| 1 | AX-11333714 | rs17659990 | 3 | 50751149 | 1.875 | 5.43E-09 |
| 2 | AX-11642476 | rs7742915 | 6 | 38147745 | 1.308 | 8.52E-08 |
| 3 | AX-11256416 | rs1362126 | 6 | 29691019 | 0.7497 | 1.27E-07 |
| 4 | AX-11292018 | rs16944613 | 15 | 91139098 | 1.319 | 1.49E-07 |
| 5 | AX-11240830 | rs13129679 | 4 | 2576102 | 2.3 | 2.38E-07 |
| 6 | AX-11233548 | rs12953717 | 18 | 46453929 | 1.261 | 3.00E-07 |
| 7 | AX-11596121 | rs694339 | 18 | 70221980 | 1.792 | 3.81E-07 |
| 8 | AX-11216902 | rs12593759 | 15 | 91202601 | 1.302 | 4.74E-07 |
| 9 | AX-11624322 | rs742223 | 6 | 11705700 | 0.6007 | 5.47E-07 |
| 10 | AX-11242701 | rs13170463 | 5 | 7983252 | 1.62 | 6.12E-07 |
| 11 | AX-11373349 | rs2184857 | 1 | 240081747 | 0.789 | 7.10E-07 |
| 12 | AX-11538691 | rs4954585 | 2 | 136998394 | 1.258 | 9.84E-07 |
| 13 | AX-11478264 | rs3753366 | 1 | 68195133 | 1.326 | 1.26E-06 |
| 14 | AX-11380285 | rs2272613 | 8 | 1873540 | 1.504 | 1.54E-06 |
| 15 | AX-11286848 | rs16887197 | 8 | 116246667 | 0.6398 | 1.90E-06 |
| 16 | AX-11203107 | rs12378124 | 9 | 9668869 | 1.41 | 2.15E-06 |
| 17 | AX-11651385 | rs7873807 | 9 | 7582632 | 1.246 | 2.71E-06 |
| 18 | AX-11710831 | rs9929215 | 16 | 86521490 | 1.767 | 2.93E-06 |
| 19 | AX-11152099 | rs11224824 | 11 | 101409138 | 1.367 | 2.96E-06 |
| 20 | AX-11437554 | rs323473 | 13 | 105359644 | 1.498 | 3.22E-06 |
| 21 | AX-11596483 | rs6948772 | 7 | 155400895 | 0.7979 | 3.57E-06 |
| 22 | AX-11538243 | rs4949330 | 1 | 31449766 | 1.802 | 3.62E-06 |
| 23 | AX-11524474 | rs476605 | 16 | 5429577 | 0.8034 | 3.94E-06 |
| 24 | AX-11676133 | rs926340 | 22 | 31139318 | 1.235 | 4.47E-06 |
| 25 | AX-11146879 | rs11144217 | 9 | 77696677 | 1.276 | 4.75E-06 |
| 26 | AX-11251054 | rs13355819 | 5 | 90576279 | 0.8048 | 5.08E-06 |
| 27 | AX-11448714 | rs34541550 | 7 | 29107992 | 1.302 | 5.23E-06 |
| 28 | AX-11615721 | rs7226855 | 18 | 46454048 | 0.8128 | 5.83E-06 |
| 29 | AX-11116485 | rs1056107 | 9 | 115087127 | 0.8136 | 6.35E-06 |
| 30 | AX-11655161 | rs7942260 | 11 | 94345502 | 0.6945 | 7.28E-06 |
| 31 | AX-11231785 | rs12913832 | 15 | 28365618 | 1.252 | 7.34E-06 |
| 32 | AX-11217657 | rs12604637 | 18 | 8556716 | 1.346 | 7.53E-06 |
| 33 | AX-11176967 | rs11812485 | 10 | 83048037 | 0.6145 | 7.58E-06 |
| 34 | AX-11136632 | rs10989517 | 9 | 104222708 | 1.494 | 7.65E-06 |
| 35 | AX-11546389 | rs5753618 | 22 | 31838518 | 1.274 | 7.69E-06 |
| 36 | AX-11547916 | rs582962 | 6 | 69521978 | 1.236 | 8.00E-06 |
| 37 | AX-11203106 | rs12378118 | 9 | 6033278 | 0.7609 | 8.07E-06 |
| 38 | AX-11688041 | rs9458376 | 6 | 162219854 | 2.005 | 8.09E-06 |
| 39 | AX-11674678 | rs917543 | 16 | 7743747 | 0.8051 | 8.14E-06 |
| 40 | AX-11219147 | rs12627687 | 21 | 35517484 | 1.3 | 8.34E-06 |
| 41 | AX-11654244 | rs7927484 | 11 | 123138960 | 1.287 | 8.75E-06 |
| 42 | AX-11352801 | rs1920324 | 3 | 103880034 | 0.7299 | 9.13E-06 |
| 43 | AX-11582413 | rs674617 | 18 | 9701782 | 1.408 | 9.14E-06 |
| 44 | AX-11666190 | rs811100 | 6 | 24547027 | 1.553 | 9.24E-06 |
| 45 | AX-11615364 | rs7221059 | 17 | 74988444 | 0.7641 | 1.02E-05 |
| 46 | AX-11199505 | rs12296006 | 11 | 98229808 | 1.428 | 1.03E-05 |
| 47 | AX-11224232 | rs1270639 | 7 | 157455459 | 1.345 | 1.05E-05 |
| 48 | AX-11668305 | rs825836 | 16 | 73542760 | 1.31 | 1.09E-05 |
| 49 | AX-11176862 | rs11809649 | 1 | 12808591 | 1.902 | 1.11E-05 |
| 50 | AX-11417427 | rs28457812 | 8 | 9211166 | 0.7888 | 1.14E-05 |
| 51 | AX-11356568 | rs1967855 | 14 | 90009698 | 1.304 | 1.20E-05 |
| 52 | AX-11530892 | rs4849957 | 2 | 4288725 | 1.221 | 1.21E-05 |
| 53 | AX-11446734 | rs34426783 | 4 | 2587020 | 1.651 | 1.21E-05 |
| 54 | AX-11087719 | rs10019252 | 4 | 40002314 | 0.7995 | 1.22E-05 |
| 55 | AX-11283097 | rs16845107 | 3 | 113127991 | 0.642 | 1.25E-05 |
| 56 | AX-11370821 | rs2152775 | 13 | 105348972 | 1.462 | 1.27E-05 |
| 57 | AX-11210573 | rs12500234 | 4 | 39619870 | 0.8138 | 1.28E-05 |
| 58 | AX-11126699 | rs10848804 | 12 | 3222935 | 1.231 | 1.29E-05 |
| 59 | AX-11186831 | rs1202519 | 1 | 230947140 | 0.8156 | 1.30E-05 |
| 60 | AX-11176614 | rs11803759 | 1 | 14665131 | 1.691 | 1.31E-05 |
| 61 | AX-11538673 | rs4954402 | 2 | 137012402 | 1.263 | 1.31E-05 |
| 62 | AX-11502603 | rs4361767 | 8 | 9217449 | 0.7707 | 1.31E-05 |
| 63 | AX-11686307 | rs940592 | 9 | 115018065 | 0.8199 | 1.33E-05 |
| 64 | AX-11546307 | rs5752176 | 22 | 25940733 | 1.352 | 1.33E-05 |
| 65 | AX-11260378 | rs1404228 | 7 | 113050788 | 1.403 | 1.41E-05 |
| 66 | AX-11714695 | rs999493 | 17 | 46625519 | 0.8148 | 1.44E-05 |
| 67 | AX-11702579 | rs979006 | 4 | 54452878 | 1.324 | 1.50E-05 |
| 68 | AX-11466528 | rs35589484 | 13 | 55826792 | 1.402 | 1.51E-05 |
| 69 | AX-11433889 | rs3107431 | 4 | 107735838 | 1.311 | 1.59E-05 |
| 70 | AX-11675388 | rs924873 | 5 | 168247565 | 1.217 | 1.60E-05 |
| 71 | AX-11584546 | rs6775899 | 3 | 121319824 | 1.271 | 1.60E-05 |
| 72 | AX-11631749 | rs7587054 | 2 | 81171525 | 1.363 | 1.70E-05 |
| 73 | AX-11711235 | rs9935437 | 16 | 52492479 | 0.7364 | 1.72E-05 |
| 74 | AX-11646401 | rs7800235 | 7 | 25135566 | 1.259 | 1.75E-05 |
| 75 | AX-11633133 | rs7606512 | 2 | 235609674 | 1.607 | 1.81E-05 |
| 76 | AX-11310281 | rs17148423 | 4 | 71121196 | 1.328 | 1.83E-05 |
| 77 | AX-11203569 | rs12401936 | 1 | 163328756 | 1.261 | 1.92E-05 |
| 78 | AX-11369864 | rs2139727 | 12 | 119083418 | 1.619 | 1.92E-05 |
| 79 | AX-11359120 | rs2000796 | 11 | 123136552 | 1.285 | 1.94E-05 |
| 80 | AX-11669601 | rs849565 | 2 | 206610051 | 0.7791 | 1.95E-05 |
| 81 | AX-11537438 | rs4939827 | 18 | 46453463 | 0.8235 | 1.96E-05 |
| 82 | AX-11616460 | rs7236448 | 18 | 6913534 | 0.8016 | 1.96E-05 |
| 83 | AX-11428378 | rs294813 | 9 | 10203246 | 1.326 | 1.98E-05 |
| 84 | AX-11254443 | rs13437464 | 6 | 133969599 | 1.887 | 2.01E-05 |
| 85 | AX-11681548 | rs9329185 | 8 | 9204425 | 0.8003 | 2.08E-05 |
| 86 | AX-11592603 | rs6893517 | 5 | 148250573 | 1.217 | 2.12E-05 |
| 87 | AX-11344563 | rs1799945 | 6 | 26091179 | 0.7412 | 2.16E-05 |
| 88 | AX-11562550 | rs637041 | 10 | 6236409 | 1.225 | 2.16E-05 |
| 89 | AX-11566907 | rs6477891 | 9 | 114962309 | 0.8244 | 2.25E-05 |
| 90 | AX-11132895 | rs10936764 | 3 | 173336649 | 1.227 | 2.27E-05 |
| 91 | AX-11611381 | rs7165476 | 15 | 91151948 | 1.236 | 2.32E-05 |
| 92 | AX-11221914 | rs12667108 | 7 | 5133936 | 0.7487 | 2.34E-05 |
| 93 | AX-11525289 | rs4775048 | 15 | 58748517 | 0.7281 | 2.40E-05 |
| 94 | AX-11530011 | rs4836891 | 9 | 125273574 | 1.415 | 2.41E-05 |
| 95 | AX-11569436 | rs6517312 | 21 | 37277069 | 1.676 | 2.44E-05 |
| 96 | AX-11399912 | rs25876 | 5 | 132793068 | 0.821 | 2.45E-05 |
| 97 | AX-11245374 | rs13230698 | 7 | 3361125 | 1.212 | 2.47E-05 |
| 98 | AX-11651479 | rs7875332 | 9 | 114927535 | 0.8232 | 2.60E-05 |
| 99 | AX-11566037 | rs6466817 | 7 | 122021647 | 1.213 | 2.67E-05 |
| 100 | AX-11646219 | rs7797378 | 7 | 132216449 | 1.314 | 2.75E-05 |
| 101 | AX-11567858 | rs6491880 | 13 | 105346831 | 1.448 | 2.86E-05 |
| 102 | AX-11432826 | rs3087967 | 11 | 111156836 | 1.241 | 2.88E-05 |
| 103 | AX-11564733 | rs6447984 | 4 | 20833134 | 0.79 | 2.88E-05 |
| 104 | AX-11557802 | rs606826 | 18 | 70197285 | 1.478 | 2.91E-05 |
| 105 | AX-11438728 | rs338241 | 1 | 58934155 | 1.524 | 2.94E-05 |
| 106 | AX-11223448 | rs12691881 | 2 | 137029668 | 1.209 | 2.94E-05 |
| 107 | AX-11253806 | rs13424039 | 2 | 4280385 | 0.8257 | 2.98E-05 |
| 108 | AX-11173142 | rs11756830 | 6 | 38148413 | 1.214 | 2.98E-05 |
| 109 | AX-11486264 | rs3888018 | 12 | 30783546 | 1.237 | 3.00E-05 |
| 110 | AX-11286482 | rs16882479 | 8 | 34443390 | 1.426 | 3.04E-05 |
| 111 | AX-11182373 | rs11927424 | 3 | 88757831 | 1.214 | 3.05E-05 |
| 112 | AX-11423734 | rs75972823 | 3 | 121334527 | 0.7771 | 3.13E-05 |
| 113 | AX-11711786 | rs9944757 | 18 | 74461083 | 0.6941 | 3.15E-05 |
| 114 | AX-11149684 | rs11189412 | 10 | 99611713 | 0.816 | 3.17E-05 |
| 115 | AX-11323366 | rs17416314 | 5 | 31804351 | 0.7048 | 3.18E-05 |
| 116 | AX-11283698 | rs16852097 | 3 | 141819038 | 1.49 | 3.25E-05 |
| 117 | AX-11310942 | rs17155568 | 10 | 14644455 | 1.698 | 3.29E-05 |
| 118 | AX-11698047 | rs962092 | 9 | 7580824 | 1.213 | 3.30E-05 |
| 119 | AX-11231931 | rs12916300 | 15 | 28410491 | 1.233 | 3.30E-05 |
| 120 | AX-11151023 | rs11209710 | 1 | 71365018 | 1.22 | 3.32E-05 |
| 121 | AX-11285376 | rs16870061 | 5 | 2578112 | 1.238 | 3.32E-05 |
| 122 | AX-11348997 | rs1875059 | 12 | 32702900 | 1.425 | 3.40E-05 |
| 123 | AX-11481813 | rs3802842 | 11 | 111171709 | 1.239 | 3.43E-05 |
| 124 | AX-11646902 | rs7806931 | 7 | 109356739 | 1.21 | 3.44E-05 |
| 125 | AX-11248741 | rs13294474 | 9 | 82815148 | 0.7935 | 3.49E-05 |
| 126 | AX-11465883 | rs35551835 | 1 | 187942253 | 1.427 | 3.49E-05 |
| 127 | AX-11406922 | rs2717886 | 7 | 25174697 | 1.249 | 3.52E-05 |
| 128 | AX-11137772 | rs11010633 | 10 | 36637021 | 1.231 | 3.60E-05 |
| 129 | AX-11240776 | rs13128318 | 4 | 156863252 | 1.239 | 3.61E-05 |
| 130 | AX-11240006 | rs13111143 | 4 | 2755447 | 1.603 | 3.63E-05 |
| 131 | AX-11460088 | rs35211132 | 4 | 187969899 | 1.282 | 3.65E-05 |
| 132 | AX-11213697 | rs12542761 | 8 | 72375596 | 1.571 | 3.65E-05 |
| 133 | AX-11110145 | rs10489877 | 1 | 192570150 | 1.241 | 3.67E-05 |
| 134 | AX-11314371 | rs17212065 | 14 | 25695907 | 1.322 | 3.67E-05 |
| 135 | AX-11487134 | rs3911097 | 3 | 168341654 | 1.474 | 3.68E-05 |
| 136 | AX-11213027 | rs12533277 | 7 | 96777930 | 1.563 | 3.69E-05 |
| 137 | AX-11136163 | rs10981340 | 9 | 115048181 | 1.215 | 3.73E-05 |
| 138 | AX-11124252 | rs10817301 | 9 | 115015710 | 1.217 | 3.74E-05 |
| 139 | AX-11405873 | rs2702934 | 8 | 6707885 | 1.293 | 3.78E-05 |
| 140 | AX-11274913 | rs1554395 | 3 | 183664873 | 0.8011 | 3.78E-05 |
| 141 | AX-11233793 | rs12958248 | 18 | 24384976 | 0.791 | 3.80E-05 |
| 142 | AX-11439661 | rs340145 | 3 | 111783125 | 0.823 | 3.91E-05 |
| 143 | AX-11141123 | rs11062055 | 12 | 2107301 | 0.7661 | 3.94E-05 |
| 144 | AX-11408568 | rs2742345 | 2 | 179568682 | 1.551 | 3.94E-05 |
| 145 | AX-11552886 | rs596240 | 18 | 70223420 | 1.754 | 3.98E-05 |
| 146 | AX-11329726 | rs17578878 | 4 | 37900725 | 1.338 | 4.01E-05 |
| 147 | AX-11577705 | rs6676084 | 1 | 197094030 | 1.222 | 4.08E-05 |
| 148 | AX-11598318 | rs6974075 | 7 | 3392071 | 1.205 | 4.08E-05 |
| 149 | AX-11464398 | rs35464786 | 4 | 37915414 | 1.351 | 4.08E-05 |
| 150 | AX-11535636 | rs4912239 | 1 | 56878569 | 1.932 | 4.10E-05 |
| 151 | AX-11221057 | rs12654325 | 5 | 2892699 | 0.7678 | 4.10E-05 |
| 152 | AX-11414287 | rs2834372 | 21 | 35427276 | 1.204 | 4.10E-05 |
| 153 | AX-11354748 | rs1944158 | 11 | 100138732 | 1.46 | 4.12E-05 |
| 154 | AX-11490870 | rs4121594 | 18 | 13511842 | 1.208 | 4.13E-05 |
| 155 | AX-11096280 | rs10152186 | 15 | 58791920 | 1.228 | 4.13E-05 |
| 156 | AX-11391402 | rs2426054 | 20 | 46567681 | 1.317 | 4.18E-05 |
| 157 | AX-11206330 | rs12440000 | 15 | 32412930 | 0.7638 | 4.22E-05 |
| 158 | AX-11421506 | rs28655514 | 14 | 62493964 | 1.451 | 4.31E-05 |
| 159 | AX-11274230 | rs1547553 | 18 | 77926197 | 1.293 | 4.33E-05 |
| 160 | AX-11286156 | rs16878549 | 5 | 7279325 | 1.503 | 4.46E-05 |
| 161 | AX-11483431 | rs3822784 | 5 | 9254823 | 0.628 | 4.46E-05 |
| 162 | AX-11595889 | rs693998 | 7 | 123465342 | 1.242 | 4.50E-05 |
| 163 | AX-11155411 | rs1132990 | 19 | 50028163 | 1.244 | 4.53E-05 |
| 164 | AX-11447892 | rs344945 | 3 | 187845956 | 1.236 | 4.56E-05 |
| 165 | AX-11634141 | rs7620457 | 3 | 183747266 | 0.7834 | 4.65E-05 |
| 166 | AX-11342243 | rs17825286 | 17 | 69336174 | 1.391 | 4.67E-05 |
| 167 | AX-11423669 | rs2876841 | 7 | 39537647 | 0.723 | 4.68E-05 |
| 168 | AX-11536845 | rs4932358 | 15 | 91172099 | 1.269 | 4.74E-05 |
| 169 | AX-11429462 | rs2966732 | 5 | 136576511 | 1.213 | 4.75E-05 |
| 170 | AX-11273662 | rs1542546 | 4 | 189528882 | 0.76 | 4.78E-05 |
| 171 | AX-11548137 | N/A | 21 | 35435374 | 0.8272 | 4.80E-05 |
| 172 | AX-11513427 | rs111388405 | 11 | 111172179 | 1.233 | 4.83E-05 |
| 173 | AX-11497312 | rs4235253 | 4 | 8045863 | 1.222 | 4.92E-05 |
| 174 | AX-11495974 | rs4146264 | 4 | 34872407 | 0.4779 | 4.96E-05 |
| 175 | AX-11595945 | rs6940879 | 6 | 112492645 | 1.43 | 5.02E-05 |
| 176 | AX-11694740 | rs956089 | 7 | 52611144 | 1.35 | 5.03E-05 |
| 177 | AX-11273882 | rs1544428 | 22 | 30307235 | 0.5753 | 5.05E-05 |
| 178 | AX-11426794 | rs2914341 | 5 | 170703078 | 1.273 | 5.05E-05 |
| 179 | AX-11165254 | rs11648673 | 16 | 377794 | 0.804 | 5.06E-05 |
| 180 | AX-11678209 | rs9290397 | 3 | 170360106 | 0.7531 | 5.11E-05 |
| 181 | AX-11612662 | rs7182229 | 15 | 58765183 | 0.7478 | 5.19E-05 |
| 182 | AX-11384077 | rs2306792 | 3 | 113081835 | 0.533 | 5.30E-05 |
| 183 | AX-11363422 | rs2052221 | 14 | 73370334 | 1.922 | 5.31E-05 |
| 184 | AX-11254927 | rs134702 | 22 | 26494612 | 1.227 | 5.32E-05 |
| 185 | AX-11400152 | rs2591520 | 5 | 14902925 | 0.6634 | 5.32E-05 |
| 186 | AX-11287981 | rs16899063 | 8 | 102176188 | 2 | 5.39E-05 |
| 187 | AX-11241468 | rs13143632 | 4 | 77772783 | 0.728 | 5.40E-05 |
| 188 | AX-11698344 | rs9632774 | 7 | 139137562 | 1.246 | 5.42E-05 |
| 189 | AX-11516937 | rs4672884 | 2 | 219182481 | 0.8288 | 5.43E-05 |
| 190 | AX-11124251 | rs10817277 | 9 | 114934532 | 1.208 | 5.43E-05 |
| 191 | AX-11487334 | rs3914272 | 3 | 171558536 | 1.277 | 5.49E-05 |
| 192 | AX-11368883 | rs2125062 | 10 | 97980721 | 1.2 | 5.53E-05 |
| 193 | AX-11219660 | rs1263573 | 7 | 157435350 | 1.204 | 5.58E-05 |
| 194 | AX-11275238 | rs1557614 | 10 | 125247462 | 0.823 | 5.62E-05 |
| 195 | AX-11256988 | rs1367993 | 18 | 70215279 | 1.444 | 5.62E-05 |
| 196 | AX-11268998 | rs1496042 | 1 | 152159773 | 1.2 | 5.65E-05 |
| 197 | AX-11705503 | rs9842683 | 3 | 65849355 | 0.8064 | 5.65E-05 |
| 198 | AX-11369564 | rs2135159 | 2 | 213214465 | 1.29 | 5.67E-05 |
| 199 | AX-11150775 | rs11206733 | 1 | 56541706 | 1.251 | 5.75E-05 |
| 200 | AX-11675276 | rs923750 | 2 | 81152623 | 1.333 | 5.75E-05 |

| **B vs. C** | | | | | | |
| --- | --- | --- | --- | --- | --- | --- |
| RANK | SNP | RS ID | CHR | BP | OR | P |
| 1 | AX-11655269 | rs7944251 | 11 | 91996431 | 0.6432 | 2.40E-06 |
| 2 | AX-11334750 | rs17679617 | 16 | 83308001 | 1.609 | 2.68E-06 |
| 3 | AX-11276363 | N/A | 12 | 71074730 | 1.667 | 4.47E-06 |
| 4 | AX-11320385 | rs17342699 | 1 | 34339953 | 3.145 | 5.69E-06 |
| 5 | AX-11364883 | rs2066844 | 16 | 50745926 | 2.345 | 7.79E-06 |
| 6 | AX-11367824 | rs2110007 | 7 | 21193341 | 0.4907 | 8.59E-06 |
| 7 | AX-11645152 | rs7781557 | 7 | 102481891 | 0.6634 | 8.63E-06 |
| 8 | AX-11624436 | rs7425956 | 2 | 233945565 | 0.7088 | 8.76E-06 |
| 9 | AX-11539471 | rs4964759 | 12 | 109203811 | 0.7149 | 9.36E-06 |
| 10 | AX-11179111 | rs11858960 | 15 | 33534019 | 0.708 | 1.16E-05 |
| 11 | AX-11615025 | rs7215468 | 17 | 37189723 | 0.7146 | 1.18E-05 |
| 12 | AX-11331956 | rs17626900 | 2 | 195841000 | 0.4384 | 1.21E-05 |
| 13 | AX-11656473 | rs7962947 | 12 | 109141435 | 0.7 | 1.32E-05 |
| 14 | AX-11281058 | rs1676969 | 8 | 4577097 | 0.6721 | 1.54E-05 |
| 15 | AX-11690799 | rs9509469 | 13 | 21527762 | 1.409 | 1.65E-05 |
| 16 | AX-11702579 | rs979006 | 4 | 54452878 | 1.602 | 1.68E-05 |
| 17 | AX-11380410 | rs2273765 | 9 | 77697526 | 0.4833 | 1.74E-05 |
| 18 | AX-11142207 | rs1107573 | 11 | 34456208 | 0.435 | 2.54E-05 |
| 19 | AX-11501113 | rs4322510 | 13 | 21526655 | 1.419 | 3.04E-05 |
| 20 | AX-11299012 | rs17025532 | 4 | 149879415 | 1.447 | 3.31E-05 |
| 21 | AX-11418828 | rs28531799 | 9 | 79575623 | 1.895 | 3.45E-05 |
| 22 | AX-11456145 | N/A | 8 | 18148918 | 0.7127 | 3.52E-05 |
| 23 | AX-11187095 | rs12029561 | 1 | 182454652 | 1.914 | 3.64E-05 |
| 24 | AX-11200057 | rs12310405 | 12 | 71043802 | 1.455 | 3.67E-05 |
| 25 | AX-11200273 | rs12316508 | 12 | 91036272 | 0.6939 | 3.73E-05 |
| 26 | AX-11182733 | rs11934382 | 4 | 149964525 | 1.366 | 3.97E-05 |
| 27 | AX-11326186 | rs17487246 | 4 | 149970086 | 1.651 | 4.22E-05 |
| 28 | AX-11160942 | rs11592638 | 10 | 89399224 | 1.383 | 4.50E-05 |
| 29 | AX-11240830 | rs13129679 | 4 | 2576102 | 3.421 | 4.66E-05 |
| 30 | AX-11375683 | rs2215040 | 7 | 102496594 | 0.7092 | 4.74E-05 |
| 31 | AX-11276757 | rs1571648 | 9 | 27332772 | 0.677 | 4.79E-05 |
| 32 | AX-11174174 | rs11770345 | 7 | 24483969 | 0.7278 | 5.55E-05 |
| 33 | AX-11647152 | rs7810629 | 7 | 96989624 | 1.357 | 5.71E-05 |
| 34 | AX-11464908 | N/A | 10 | 100724817 | 2.918 | 5.72E-05 |
| 35 | AX-11623771 | rs739514 | 4 | 149903764 | 1.349 | 6.07E-05 |
| 36 | AX-11501634 | rs4335931 | 2 | 233955723 | 0.7379 | 6.34E-05 |
| 37 | AX-11475052 | rs36095535 | 2 | 12289869 | 1.557 | 7.18E-05 |
| 38 | AX-11692240 | rs952810 | 2 | 7370196 | 0.7381 | 7.24E-05 |
| 39 | AX-11335812 | rs17700253 | 13 | 97557480 | 2.243 | 7.44E-05 |
| 40 | AX-11240021 | rs13111399 | 4 | 149941650 | 1.382 | 7.57E-05 |
| 41 | AX-11341249 | rs17805809 | 10 | 117894402 | 1.561 | 7.70E-05 |
| 42 | AX-11335482 | rs17694321 | 3 | 6994953 | 1.389 | 7.97E-05 |
| 43 | AX-11369564 | rs2135159 | 2 | 213214465 | 1.52 | 8.23E-05 |
| 44 | AX-11711330 | rs9937227 | 16 | 83306442 | 1.626 | 8.35E-05 |
| 45 | AX-11105519 | rs1040577 | 20 | 19907294 | 1.341 | 8.54E-05 |
| 46 | AX-11698685 | rs9639327 | 7 | 19340930 | 0.6972 | 9.11E-05 |
| 47 | AX-11136747 | rs10991415 | 9 | 107686354 | 1.54 | 9.25E-05 |
| 48 | AX-11173852 | rs11766140 | 7 | 24519656 | 1.34 | 9.28E-05 |
| 49 | AX-11116853 | rs1062708 | 19 | 49513273 | 1.343 | 9.39E-05 |
| 50 | AX-11443749 | rs34250521 | 15 | 59507953 | 0.6995 | 9.43E-05 |
| 51 | AX-11433889 | rs3107431 | 4 | 107735838 | 1.483 | 9.44E-05 |
| 52 | AX-11150713 | rs11205451 | 1 | 48647860 | 2.753 | 9.55E-05 |
| 53 | AX-11578799 | rs6693264 | 1 | 48556937 | 2.752 | 9.55E-05 |
| 54 | AX-11255520 | rs1353039 | 8 | 18193939 | 0.7458 | 0.0001005 |
| 55 | AX-11637932 | rs7673586 | 4 | 149979341 | 1.329 | 0.0001022 |
| 56 | AX-11229314 | rs1285875 | 6 | 7115927 | 0.7116 | 0.0001079 |
| 57 | AX-11590140 | rs6854895 | 4 | 78851940 | 0.7207 | 0.0001096 |
| 58 | AX-11137764 | rs1101047 | 7 | 147392446 | 0.689 | 0.0001098 |
| 59 | AX-11424350 | rs28836770 | 3 | 190977465 | 1.347 | 0.0001116 |
| 60 | AX-11315256 | rs17230787 | 10 | 33708150 | 1.515 | 0.0001127 |
| 61 | AX-11415595 | rs2837983 | 21 | 42616757 | 0.6964 | 0.0001154 |
| 62 | AX-11187942 | rs12044374 | 1 | 94635986 | 1.336 | 0.0001204 |
| 63 | AX-11261780 | rs1420161 | 7 | 96988034 | 1.362 | 0.0001225 |
| 64 | AX-11687279 | rs9438466 | 1 | 203984064 | 1.44 | 0.0001249 |
| 65 | AX-11179550 | rs11868791 | 17 | 37203360 | 0.751 | 0.0001269 |
| 66 | AX-11395729 | rs250956 | 2 | 234993563 | 1.439 | 0.000128 |
| 67 | AX-11206536 | rs12442900 | 15 | 87733835 | 1.327 | 0.00013 |
| 68 | AX-11220315 | rs12644174 | 4 | 149878147 | 1.348 | 0.0001302 |
| 69 | AX-11332730 | rs17642361 | 8 | 18147962 | 0.7302 | 0.0001326 |
| 70 | AX-11455993 | rs34964149 | 9 | 84269234 | 1.403 | 0.0001333 |
| 71 | AX-11648821 | rs7834906 | 8 | 18175677 | 0.7194 | 0.0001341 |
| 72 | AX-11703414 | rs9811423 | 3 | 112822322 | 1.329 | 0.0001342 |
| 73 | AX-11517415 | rs4678773 | 3 | 35457937 | 1.476 | 0.0001366 |
| 74 | AX-11623246 | rs736368 | 20 | 37020978 | 1.44 | 0.0001381 |
| 75 | AX-11499490 | rs4280941 | 6 | 162250522 | 1.707 | 0.0001411 |
| 76 | AX-11409075 | rs2755427 | 10 | 89417472 | 0.6636 | 0.0001415 |
| 77 | AX-11487498 | rs3916981 | 12 | 109215423 | 0.7305 | 0.000143 |
| 78 | AX-11277311 | rs158123 | 14 | 77560478 | 1.462 | 0.000145 |
| 79 | AX-11134865 | rs10964101 | 9 | 1943156 | 1.558 | 0.000147 |
| 80 | AX-11255665 | rs1354299 | 10 | 111241216 | 0.4894 | 0.0001472 |
| 81 | AX-11561662 | rs622199 | 3 | 73305297 | 2.122 | 0.000149 |
| 82 | AX-11125447 | rs10832688 | 11 | 16843375 | 0.7443 | 0.0001518 |
| 83 | AX-11572166 | rs6562011 | 13 | 59714710 | 0.7184 | 0.0001555 |
| 84 | AX-11359495 | rs2007478 | 3 | 105904856 | 0.733 | 0.0001591 |
| 85 | AX-11223667 | rs12696024 | 3 | 155035437 | 0.7533 | 0.0001602 |
| 86 | AX-11591414 | rs687467 | 6 | 7128076 | 0.7157 | 0.0001613 |
| 87 | AX-11476376 | rs372747 | 9 | 107978792 | 0.5921 | 0.0001619 |
| 88 | AX-11258967 | rs1389110 | 8 | 18091656 | 0.7499 | 0.0001632 |
| 89 | AX-11202675 | rs12369524 | 12 | 29049041 | 0.6544 | 0.0001635 |
| 90 | AX-11227480 | rs12799875 | 11 | 119910275 | 0.7317 | 0.0001717 |
| 91 | AX-11349269 | rs1877720 | 1 | 48824407 | 2.307 | 0.0001748 |
| 92 | AX-11368659 | rs2121701 | 11 | 45645306 | 0.7324 | 0.0001772 |
| 93 | AX-11145388 | rs11125447 | 2 | 53055130 | 1.471 | 0.0001814 |
| 94 | AX-11304282 | rs17080342 | 13 | 19926967 | 0.6128 | 0.0001815 |
| 95 | AX-11534253 | rs4894988 | 3 | 105874441 | 0.7462 | 0.0001906 |
| 96 | AX-11487270 | rs3913351 | 15 | 87755594 | 1.317 | 0.0001938 |
| 97 | AX-11248595 | rs13291117 | 9 | 107679425 | 1.775 | 0.0001993 |
| 98 | AX-11140258 | rs11049925 | 12 | 29087868 | 0.6521 | 0.0002048 |
| 99 | AX-11312645 | rs17179503 | 7 | 81987289 | 0.5114 | 0.0002072 |
| 100 | AX-11701180 | rs972534 | 11 | 119917892 | 0.7347 | 0.0002078 |
| 101 | AX-11508209 | rs450320 | 4 | 187565156 | 0.6214 | 0.0002116 |
| 102 | AX-11420654 | rs28615938 | 15 | 35106527 | 2.304 | 0.0002146 |
| 103 | AX-11188211 | rs12049402 | 1 | 199126302 | 1.413 | 0.00022 |
| 104 | AX-11261345 | rs1415123 | 13 | 21541027 | 1.325 | 0.0002201 |
| 105 | AX-11524238 | rs4763346 | 12 | 13604672 | 0.663 | 0.0002349 |
| 106 | AX-11486440 | rs3892275 | 1 | 181054522 | 0.7514 | 0.0002351 |
| 107 | AX-11353148 | rs1924948 | 13 | 73087010 | 1.342 | 0.0002357 |
| 108 | AX-11124326 | rs10817971 | 9 | 119766782 | 1.384 | 0.0002361 |
| 109 | AX-11460370 | rs35226739 | 3 | 7470143 | 2.599 | 0.0002368 |
| 110 | AX-11117997 | rs10741077 | 10 | 25639985 | 1.37 | 0.0002388 |
| 111 | AX-11234336 | rs12968813 | 18 | 528165 | 0.5189 | 0.00024 |
| 112 | AX-11235734 | rs13006576 | 2 | 30068069 | 0.749 | 0.000241 |
| 113 | AX-11357705 | rs1984555 | 17 | 48836197 | 1.601 | 0.0002479 |
| 114 | AX-11175604 | rs11787299 | 8 | 18141181 | 0.7495 | 0.0002486 |
| 115 | AX-11322058 | rs17382698 | 8 | 129139206 | 1.517 | 0.0002502 |
| 116 | AX-11219822 | rs12637835 | 3 | 11116366 | 0.6754 | 0.0002503 |
| 117 | AX-11156410 | rs11395174 | 7 | 68198342 | 1.496 | 0.0002522 |
| 118 | AX-11523602 | rs4755329 | 11 | 45596066 | 0.6149 | 0.0002565 |
| 119 | AX-11478280 | rs375371 | 9 | 107978322 | 0.6295 | 0.0002617 |
| 120 | AX-11243877 | rs13195583 | 6 | 4575910 | 0.733 | 0.0002631 |
| 121 | AX-11235154 | rs12993382 | 2 | 30110249 | 0.7438 | 0.0002636 |
| 122 | AX-11671979 | rs882820 | 16 | 4256005 | 1.401 | 0.000264 |
| 123 | AX-11560466 | rs6127923 | 20 | 55646198 | 1.437 | 0.0002648 |
| 124 | AX-11123045 | rs10803233 | 1 | 236189722 | 1.321 | 0.0002652 |
| 125 | AX-11300357 | rs17040818 | 12 | 108988757 | 1.412 | 0.0002665 |
| 126 | AX-11689215 | rs947937 | 11 | 91991008 | 1.312 | 0.0002679 |
| 127 | AX-11464523 | rs35471617 | 6 | 56033094 | 1.651 | 0.0002705 |
| 128 | AX-11530083 | rs4837864 | 9 | 119544433 | 0.6433 | 0.0002712 |
| 129 | AX-11121338 | rs1078174 | 18 | 44168737 | 1.359 | 0.0002749 |
| 130 | AX-11430909 | rs29964 | 5 | 16365725 | 1.63 | 0.0002757 |
| 131 | AX-11233433 | rs12950712 | 17 | 14418596 | 0.742 | 0.0002769 |
| 132 | AX-11502970 | rs437061 | 6 | 4563921 | 0.7353 | 0.0002828 |
| 133 | AX-11159457 | rs115692 | 11 | 37110994 | 0.7372 | 0.0002851 |
| 134 | AX-11656204 | rs7959539 | 12 | 109134980 | 1.316 | 0.0002854 |
| 135 | AX-11135715 | rs10976035 | 9 | 7091968 | 0.7094 | 0.0002861 |
| 136 | AX-11110033 | rs10489270 | 1 | 173157764 | 1.518 | 0.0002874 |
| 137 | AX-11341690 | rs17814410 | 12 | 71017447 | 1.497 | 0.0002901 |
| 138 | AX-11419832 | rs2857640 | 22 | 30056945 | 0.7635 | 0.0002923 |
| 139 | AX-11196452 | rs12214664 | 6 | 40583870 | 1.845 | 0.0002932 |
| 140 | AX-11465883 | rs35551835 | 1 | 187942253 | 1.679 | 0.0002965 |
| 141 | AX-11366685 | rs2088823 | 1 | 19774133 | 1.324 | 0.0002979 |
| 142 | AX-11669725 | N/A | 7 | 147389581 | 0.6708 | 0.000301 |
| 143 | AX-11605908 | rs7087785 | 10 | 6984954 | 1.409 | 0.0003057 |
| 144 | AX-11655278 | rs7944397 | 11 | 34455309 | 0.6641 | 0.0003058 |
| 145 | AX-11429280 | rs2964144 | 5 | 5350574 | 1.304 | 0.000306 |
| 146 | AX-11184962 | rs11979398 | 7 | 96983349 | 1.495 | 0.0003094 |
| 147 | AX-11566379 | rs6471070 | 8 | 133482984 | 0.7023 | 0.0003095 |
| 148 | AX-11282521 | rs16838311 | 4 | 6142049 | 2.011 | 0.00031 |
| 149 | AX-11409851 | rs277354 | 1 | 75291073 | 1.314 | 0.0003173 |
| 150 | AX-11577846 | rs6678122 | 1 | 62301986 | 1.715 | 0.0003176 |
| 151 | AX-11396496 | rs2520490 | 4 | 149911469 | 1.311 | 0.0003183 |
| 152 | AX-11685929 | rs9400697 | 6 | 114484080 | 0.6685 | 0.0003186 |
| 153 | AX-11140829 | rs11058824 | 12 | 127296840 | 0.5181 | 0.0003279 |
| 154 | AX-11341545 | rs17811806 | 10 | 111617509 | 1.535 | 0.000334 |
| 155 | AX-11137857 | rs11012183 | 10 | 20892753 | 0.3744 | 0.0003342 |
| 156 | AX-11608751 | rs7130553 | 11 | 21536418 | 0.7676 | 0.0003345 |
| 157 | AX-11269404 | rs1500059 | 12 | 33164875 | 0.3825 | 0.0003403 |
| 158 | AX-11527894 | rs4809962 | 20 | 52931148 | 0.7516 | 0.0003418 |
| 159 | AX-11425473 | rs2894796 | 6 | 51950577 | 0.4958 | 0.0003421 |
| 160 | AX-11324897 | rs17455085 | 8 | 119256856 | 0.598 | 0.0003435 |
| 161 | AX-11332564 | rs1763894 | 10 | 61286319 | 1.32 | 0.000346 |
| 162 | AX-11305489 | rs17093488 | 14 | 96293259 | 0.7234 | 0.0003491 |
| 163 | AX-11637456 | rs7666738 | 4 | 98968077 | 1.342 | 0.0003492 |
| 164 | AX-11653309 | rs7911684 | 10 | 97971928 | 0.7649 | 0.0003589 |
| 165 | AX-11268872 | rs1494738 | 3 | 30885806 | 0.7554 | 0.0003602 |
| 166 | AX-11678330 | rs9291881 | 5 | 66567056 | 0.6187 | 0.0003612 |
| 167 | AX-11209697 | rs12488536 | 3 | 29433099 | 0.6419 | 0.0003653 |
| 168 | AX-11377481 | rs2238402 | 16 | 3280157 | 1.455 | 0.0003673 |
| 169 | AX-11532814 | rs4875228 | 8 | 6016650 | 0.7404 | 0.0003676 |
| 170 | AX-11150514 | rs11202472 | 10 | 89376952 | 1.311 | 0.0003694 |
| 171 | AX-11642976 | rs7750140 | 6 | 154573145 | 0.6568 | 0.0003698 |
| 172 | AX-11199505 | rs12296006 | 11 | 98229808 | 1.637 | 0.0003711 |
| 173 | AX-11642494 | rs7743188 | 6 | 54773639 | 0.6584 | 0.0003711 |
| 174 | AX-11573005 | rs6573990 | 14 | 25212506 | 1.322 | 0.0003742 |
| 175 | AX-11407355 | rs272415 | 19 | 55123632 | 1.887 | 0.000376 |
| 176 | AX-11503894 | rs4394996 | 14 | 20264019 | 0.5618 | 0.0003787 |
| 177 | AX-11321317 | rs17365438 | 15 | 76833838 | 0.6507 | 0.0003787 |
| 178 | AX-11195855 | rs1220591 | 13 | 24812797 | 1.498 | 0.0003788 |
| 179 | AX-11297127 | rs17004994 | 4 | 81730585 | 0.4885 | 0.0003789 |
| 180 | AX-11523601 | rs4755328 | 11 | 45574414 | 0.6062 | 0.0003791 |
| 181 | AX-11166586 | rs11667231 | 19 | 31119256 | 0.6047 | 0.0003809 |
| 182 | AX-11138788 | rs11026120 | 11 | 21533812 | 1.302 | 0.0003861 |
| 183 | AX-11187401 | rs12034992 | 1 | 182538063 | 1.688 | 0.0003874 |
| 184 | AX-11115184 | rs10516646 | 4 | 81605788 | 0.52 | 0.0003878 |
| 185 | AX-11212471 | rs12526148 | 6 | 161967901 | 1.339 | 0.0003923 |
| 186 | AX-11566661 | rs6475098 | 9 | 16948004 | 1.367 | 0.0003932 |
| 187 | AX-11587407 | rs6815134 | 4 | 189959922 | 1.522 | 0.0003943 |
| 188 | AX-11584547 | rs677591 | 11 | 78402702 | 1.551 | 0.0003949 |
| 189 | AX-11185863 | rs1199742 | 13 | 21529256 | 1.35 | 0.0003996 |
| 190 | AX-11270945 | rs1516561 | 3 | 6971081 | 1.365 | 0.0003996 |
| 191 | AX-11123043 | rs10803215 | 1 | 236171251 | 1.309 | 0.0004047 |
| 192 | AX-11199596 | rs12298541 | 12 | 66306441 | 1.322 | 0.0004098 |
| 193 | AX-11227598 | rs12803411 | 11 | 19081541 | 0.7655 | 0.0004144 |
| 194 | AX-11438438 | rs33409 | 5 | 150038265 | 0.7663 | 0.0004148 |
| 195 | AX-11527436 | rs4801433 | 19 | 57646570 | 1.308 | 0.0004155 |
| 196 | AX-11372617 | rs2176075 | 14 | 90459569 | 1.828 | 0.0004161 |
| 197 | AX-11698345 | rs9632853 | 8 | 24443590 | 1.592 | 0.0004166 |
| 198 | AX-11237665 | rs13059637 | 3 | 35539993 | 1.448 | 0.0004197 |
| 199 | AX-11273865 | rs1544325 | 22 | 19931668 | 1.295 | 0.0004201 |
| 200 | AX-11102292 | rs10252449 | 7 | 96981468 | 1.389 | 0.0004204 |

| **B vs. CD** | | | | | | |
| --- | --- | --- | --- | --- | --- | --- |
| RANK | SNP | RS ID | CHR | BP | OR | P |
| 1 | AX-11557827 | rs6068583 | 20 | 52176105 | 1.554 | 3.38E-07 |
| 2 | AX-11655269 | rs7944251 | 11 | 91996431 | 0.6555 | 3.97E-07 |
| 3 | AX-11460370 | rs35226739 | 3 | 7470143 | 2.647 | 8.68E-07 |
| 4 | AX-11448821 | rs34548439 | 5 | 38831131 | 1.411 | 8.79E-07 |
| 5 | AX-11173852 | rs11766140 | 7 | 24519656 | 1.366 | 1.45E-06 |
| 6 | AX-11240830 | rs13129679 | 4 | 2576102 | 2.721 | 1.64E-06 |
| 7 | AX-11237561 | rs13055076 | 22 | 30069207 | 1.455 | 1.88E-06 |
| 8 | AX-11367824 | rs2110007 | 7 | 21193341 | 0.5075 | 2.97E-06 |
| 9 | AX-11668502 | rs829883 | 12 | 98879606 | 0.7281 | 3.68E-06 |
| 10 | AX-11477473 | rs3743102 | 15 | 83427394 | 1.452 | 5.37E-06 |
| 11 | AX-11130316 | rs10898160 | 11 | 83558374 | 1.351 | 5.87E-06 |
| 12 | AX-11368883 | rs2125062 | 10 | 97980721 | 1.335 | 6.09E-06 |
| 13 | AX-11209352 | rs12482500 | 21 | 43353610 | 1.365 | 6.42E-06 |
| 14 | AX-11438728 | rs338241 | 1 | 58934155 | 1.813 | 6.43E-06 |
| 15 | AX-11099787 | rs10208443 | 2 | 72329603 | 1.348 | 6.83E-06 |
| 16 | AX-11334750 | rs17679617 | 16 | 83308001 | 1.443 | 7.33E-06 |
| 17 | AX-11624436 | rs7425956 | 2 | 233945565 | 0.7421 | 7.38E-06 |
| 18 | AX-11415595 | rs2837983 | 21 | 42616757 | 0.6904 | 7.58E-06 |
| 19 | AX-11313235 | rs17190422 | 2 | 56856260 | 2.073 | 9.05E-06 |
| 20 | AX-11098423 | rs1018697 | 10 | 104549224 | 0.7391 | 9.08E-06 |
| 21 | AX-11555574 | rs6022591 | 20 | 52172614 | 1.385 | 9.20E-06 |
| 22 | AX-11155165 | rs1131510 | 10 | 35299085 | 1.748 | 9.48E-06 |
| 23 | AX-11433889 | rs3107431 | 4 | 107735838 | 1.451 | 9.63E-06 |
| 24 | AX-11687703 | rs9450814 | 6 | 88554867 | 1.937 | 9.79E-06 |
| 25 | AX-11376629 | rs2227714 | 7 | 100781909 | 0.3966 | 9.89E-06 |
| 26 | AX-11640496 | rs7712436 | 5 | 31485230 | 2.203 | 1.00E-05 |
| 27 | AX-11706516 | rs9857702 | 3 | 11166302 | 1.347 | 1.00E-05 |
| 28 | AX-11560357 | rs6125213 | 20 | 46666287 | 1.841 | 1.01E-05 |
| 29 | AX-11270137 | rs1507675 | 2 | 123778205 | 1.436 | 1.02E-05 |
| 30 | AX-11191221 | rs12120559 | 1 | 119844431 | 1.324 | 1.05E-05 |
| 31 | AX-11438438 | rs33409 | 5 | 150038265 | 0.7506 | 1.09E-05 |
| 32 | AX-11456145 | N/A | 8 | 18148918 | 0.7283 | 1.21E-05 |
| 33 | AX-11465883 | rs35551835 | 1 | 187942253 | 1.654 | 1.27E-05 |
| 34 | AX-11261345 | rs1415123 | 13 | 21541027 | 1.328 | 1.31E-05 |
| 35 | AX-11265096 | rs1454001 | 11 | 21262591 | 1.34 | 1.35E-05 |
| 36 | AX-11419829 | rs2857638 | 22 | 30053118 | 1.334 | 1.40E-05 |
| 37 | AX-11378216 | rs2245194 | 22 | 30048182 | 1.333 | 1.43E-05 |
| 38 | AX-11527894 | rs4809962 | 20 | 52931148 | 0.7437 | 1.58E-05 |
| 39 | AX-11134865 | rs10964101 | 9 | 1943156 | 1.507 | 1.63E-05 |
| 40 | AX-11137857 | rs11012183 | 10 | 20892753 | 0.3363 | 1.64E-05 |
| 41 | AX-11455993 | rs34964149 | 9 | 84269234 | 1.378 | 1.70E-05 |
| 42 | AX-11121453 | rs10783318 | 12 | 39070576 | 2.232 | 1.74E-05 |
| 43 | AX-11443749 | rs34250521 | 15 | 59507953 | 0.7081 | 1.76E-05 |
| 44 | AX-11352136 | rs1910243 | 12 | 38951794 | 2.286 | 1.84E-05 |
| 45 | AX-11195248 | rs1219742 | 10 | 125525042 | 1.432 | 1.86E-05 |
| 46 | AX-11615025 | rs7215468 | 17 | 37189723 | 0.7515 | 1.88E-05 |
| 47 | AX-11203107 | rs12378124 | 9 | 9668869 | 1.525 | 1.90E-05 |
| 48 | AX-11470941 | rs35851221 | 16 | 8882670 | 1.953 | 2.12E-05 |
| 49 | AX-11702579 | rs979006 | 4 | 54452878 | 1.458 | 2.14E-05 |
| 50 | AX-11689215 | rs947937 | 11 | 91991008 | 1.314 | 2.14E-05 |
| 51 | AX-11402806 | rs2641479 | 12 | 74958839 | 1.353 | 2.14E-05 |
| 52 | AX-11555382 | rs6018856 | 20 | 46636528 | 1.751 | 2.15E-05 |
| 53 | AX-11561662 | rs622199 | 3 | 73305297 | 1.901 | 2.15E-05 |
| 54 | AX-11478477 | rs3756654 | 5 | 157165246 | 1.326 | 2.17E-05 |
| 55 | AX-11219822 | rs12637835 | 3 | 11116366 | 0.6662 | 2.23E-05 |
| 56 | AX-11389076 | rs2391611 | 13 | 108670546 | 0.7095 | 2.27E-05 |
| 57 | AX-11402628 | rs2637513 | 5 | 124783400 | 1.746 | 2.52E-05 |
| 58 | AX-11307785 | rs17118810 | 14 | 47972984 | 2.085 | 2.61E-05 |
| 59 | AX-11325414 | rs1746789 | 9 | 9751064 | 1.641 | 2.63E-05 |
| 60 | AX-11274093 | rs1546308 | 12 | 21176135 | 1.612 | 2.68E-05 |
| 61 | AX-11601902 | rs7020782 | 9 | 119106881 | 1.326 | 2.71E-05 |
| 62 | AX-11697834 | rs9614158 | 22 | 30597880 | 1.323 | 2.74E-05 |
| 63 | AX-11140829 | rs11058824 | 12 | 127296840 | 0.5104 | 2.79E-05 |
| 64 | AX-11246010 | rs13244061 | 7 | 137784687 | 0.759 | 2.81E-05 |
| 65 | AX-11595945 | rs6940879 | 6 | 112492645 | 1.651 | 2.83E-05 |
| 66 | AX-11096864 | rs1016268 | 12 | 130001810 | 0.76 | 2.90E-05 |
| 67 | AX-11699745 | rs9660091 | 1 | 164732727 | 1.31 | 2.91E-05 |
| 68 | AX-11535636 | rs4912239 | 1 | 56878569 | 2.348 | 3.01E-05 |
| 69 | AX-11668705 | rs833664 | 3 | 62556868 | 1.838 | 3.07E-05 |
| 70 | AX-11571179 | rs6548744 | 3 | 81082571 | 1.373 | 3.13E-05 |
| 71 | AX-11285376 | rs16870061 | 5 | 2578112 | 1.347 | 3.14E-05 |
| 72 | AX-11619293 | rs7295114 | 12 | 13593729 | 0.7123 | 3.36E-05 |
| 73 | AX-11673730 | rs905344 | 12 | 38937391 | 2.183 | 3.38E-05 |
| 74 | AX-11261815 | rs1420413 | 12 | 98199400 | 0.7559 | 3.41E-05 |
| 75 | AX-11434638 | rs3118230 | 16 | 68709359 | 1.444 | 3.42E-05 |
| 76 | AX-11483437 | rs3822844 | 6 | 160421012 | 1.324 | 3.47E-05 |
| 77 | AX-11308890 | rs17131469 | 1 | 91961322 | 2.327 | 3.57E-05 |
| 78 | AX-11653309 | rs7911684 | 10 | 97971928 | 0.7665 | 3.67E-05 |
| 79 | AX-11387064 | rs2360087 | 16 | 8157001 | 1.332 | 3.72E-05 |
| 80 | AX-11110933 | rs10494114 | 1 | 110687962 | 1.579 | 3.78E-05 |
| 81 | AX-11685929 | rs9400697 | 6 | 114484080 | 0.6592 | 3.92E-05 |
| 82 | AX-11373171 | rs2182729 | 9 | 18917347 | 1.31 | 3.94E-05 |
| 83 | AX-11625350 | rs7459717 | 8 | 107511717 | 0.7628 | 3.95E-05 |
| 84 | AX-11707456 | rs9871676 | 3 | 114072839 | 0.7651 | 3.99E-05 |
| 85 | AX-11461801 | rs35312459 | 4 | 56721403 | 1.561 | 4.01E-05 |
| 86 | AX-11356778 | rs1972065 | 12 | 37898034 | 2.12 | 4.03E-05 |
| 87 | AX-11603569 | rs7045124 | 9 | 2661766 | 0.7173 | 4.08E-05 |
| 88 | AX-11234681 | rs12980083 | 19 | 22968938 | 1.891 | 4.20E-05 |
| 89 | AX-11489946 | rs4073374 | 3 | 123055019 | 1.44 | 4.28E-05 |
| 90 | AX-11151251 | rs11213669 | 11 | 110820365 | 1.653 | 4.31E-05 |
| 91 | AX-11154789 | N/A | 20 | 54088377 | 1.614 | 4.36E-05 |
| 92 | AX-11209979 | rs12492298 | 3 | 65720358 | 1.871 | 4.36E-05 |
| 93 | AX-11631869 | rs7588841 | 2 | 177687497 | 1.41 | 4.40E-05 |
| 94 | AX-11110145 | rs10489877 | 1 | 192570150 | 1.345 | 4.45E-05 |
| 95 | AX-11304540 | rs17083254 | 4 | 63887305 | 1.522 | 4.48E-05 |
| 96 | AX-11175604 | rs11787299 | 8 | 18141181 | 0.7536 | 4.48E-05 |
| 97 | AX-11479296 | rs3769498 | 2 | 106488411 | 0.7312 | 4.52E-05 |
| 98 | AX-11346633 | rs1843640 | 12 | 39137722 | 1.644 | 4.54E-05 |
| 99 | AX-11469116 | rs35742853 | 12 | 98160685 | 1.729 | 4.59E-05 |
| 100 | AX-11181459 | rs11907654 | 20 | 30512051 | 1.508 | 4.64E-05 |
| 101 | AX-11088818 | rs10037634 | 5 | 62385531 | 1.301 | 4.68E-05 |
| 102 | AX-11664456 | rs8082768 | 18 | 57641728 | 0.6871 | 4.72E-05 |
| 103 | AX-11305657 | rs17095503 | 1 | 74984208 | 2.417 | 4.77E-05 |
| 104 | AX-11648821 | rs7834906 | 8 | 18175677 | 0.7345 | 4.85E-05 |
| 105 | AX-11421190 | rs28639642 | 4 | 71376971 | 0.7185 | 4.87E-05 |
| 106 | AX-11590140 | rs6854895 | 4 | 78851940 | 0.741 | 4.88E-05 |
| 107 | AX-11323386 | rs17416859 | 5 | 121321055 | 1.868 | 4.90E-05 |
| 108 | AX-11524048 | rs75452819 | 12 | 128165677 | 1.538 | 4.90E-05 |
| 109 | AX-11240162 | rs13114658 | 4 | 1052009 | 1.295 | 4.91E-05 |
| 110 | AX-11402714 | rs2639270 | 3 | 81089293 | 1.362 | 4.93E-05 |
| 111 | AX-11577846 | rs6678122 | 1 | 62301986 | 1.637 | 4.93E-05 |
| 112 | AX-11366685 | rs2088823 | 1 | 19774133 | 1.309 | 5.04E-05 |
| 113 | AX-11696735 | rs959324 | 15 | 48670904 | 1.442 | 5.05E-05 |
| 114 | AX-11659506 | rs8007495 | 14 | 28933330 | 1.341 | 5.14E-05 |
| 115 | AX-11374298 | rs219551 | 2 | 21540711 | 1.416 | 5.19E-05 |
| 116 | AX-11408807 | rs274713 | 5 | 6723560 | 0.7495 | 5.37E-05 |
| 117 | AX-11493878 | rs4130464 | 2 | 228342678 | 1.732 | 5.48E-05 |
| 118 | AX-11293109 | rs16955379 | 16 | 81489373 | 2.434 | 5.50E-05 |
| 119 | AX-11603767 | rs7047972 | 9 | 1945517 | 1.584 | 5.67E-05 |
| 120 | AX-11142207 | rs1107573 | 11 | 34456208 | 0.4727 | 5.73E-05 |
| 121 | AX-11123045 | rs10803233 | 1 | 236189722 | 1.298 | 5.85E-05 |
| 122 | AX-11483928 | rs3829273 | 11 | 9048668 | 0.6755 | 5.86E-05 |
| 123 | AX-11451102 | rs34680385 | 8 | 4761302 | 0.4534 | 5.88E-05 |
| 124 | AX-11259342 | rs1393109 | 5 | 38812605 | 1.321 | 5.89E-05 |
| 125 | AX-11168682 | rs11697210 | 20 | 13873821 | 1.3 | 5.93E-05 |
| 126 | AX-11286482 | rs16882479 | 8 | 34443390 | 1.585 | 5.94E-05 |
| 127 | AX-11109103 | rs10483612 | 14 | 53034269 | 1.387 | 5.95E-05 |
| 128 | AX-11243561 | rs13188627 | 5 | 2940440 | 0.7572 | 6.05E-05 |
| 129 | AX-11483301 | rs3821212 | 2 | 58443598 | 0.6577 | 6.05E-05 |
| 130 | AX-11405873 | rs2702934 | 8 | 6707885 | 1.408 | 6.14E-05 |
| 131 | AX-11088300 | rs10028954 | 4 | 70466280 | 1.292 | 6.19E-05 |
| 132 | AX-11110033 | rs10489270 | 1 | 173157764 | 1.462 | 6.36E-05 |
| 133 | AX-11712984 | rs9963872 | 18 | 64076587 | 1.33 | 6.39E-05 |
| 134 | AX-11155261 | N/A | 1 | 74227803 | 1.585 | 6.40E-05 |
| 135 | AX-11642476 | rs7742915 | 6 | 38147745 | 1.322 | 6.43E-05 |
| 136 | AX-11374526 | rs2198895 | 19 | 31369194 | 1.38 | 6.54E-05 |
| 137 | AX-11638671 | rs7685184 | 4 | 68830891 | 1.723 | 6.65E-05 |
| 138 | AX-11329503 | rs17574363 | 1 | 74122299 | 1.528 | 6.70E-05 |
| 139 | AX-11379089 | rs2253894 | 21 | 42710566 | 1.388 | 6.95E-05 |
| 140 | AX-11559669 | rs6107151 | 20 | 26158945 | 1.437 | 6.99E-05 |
| 141 | AX-11311782 | rs17166395 | 7 | 12820897 | 1.786 | 7.02E-05 |
| 142 | AX-11332730 | rs17642361 | 8 | 18147962 | 0.7504 | 7.04E-05 |
| 143 | AX-11283359 | rs16848124 | 2 | 213123460 | 0.7445 | 7.12E-05 |
| 144 | AX-11569436 | rs6517312 | 21 | 37277069 | 1.896 | 7.16E-05 |
| 145 | AX-11657204 | rs7973380 | 12 | 39569858 | 2.24 | 7.18E-05 |
| 146 | AX-11416982 | rs28437505 | 4 | 84252514 | 1.918 | 7.21E-05 |
| 147 | AX-11300153 | rs17038353 | 1 | 119046507 | 1.966 | 7.23E-05 |
| 148 | AX-11289618 | rs16917259 | 11 | 91994044 | 0.681 | 7.45E-05 |
| 149 | AX-11317075 | rs172705 | 3 | 4345033 | 1.615 | 7.48E-05 |
| 150 | AX-11331956 | rs17626900 | 2 | 195841000 | 0.502 | 7.57E-05 |
| 151 | AX-11480707 | rs3787970 | 21 | 43651556 | 0.7731 | 7.58E-05 |
| 152 | AX-11711772 | rs9944240 | 15 | 47504936 | 1.292 | 7.65E-05 |
| 153 | AX-11478264 | rs3753366 | 1 | 68195133 | 1.374 | 7.80E-05 |
| 154 | AX-11337675 | rs17737005 | 12 | 81795085 | 2.003 | 7.81E-05 |
| 155 | AX-11563839 | rs6435236 | 2 | 205253405 | 0.7463 | 7.83E-05 |
| 156 | AX-11127411 | rs10859713 | 12 | 94907751 | 0.7716 | 7.86E-05 |
| 157 | AX-11524710 | rs4769 | 11 | 8968885 | 0.6521 | 7.88E-05 |
| 158 | AX-11437070 | rs321198 | 7 | 137029838 | 1.287 | 7.93E-05 |
| 159 | AX-11112099 | rs10500593 | 11 | 4093962 | 2.019 | 8.07E-05 |
| 160 | AX-11180935 | rs11896830 | 2 | 208237126 | 1.429 | 8.12E-05 |
| 161 | AX-11117874 | rs10739534 | 9 | 122071105 | 1.287 | 8.23E-05 |
| 162 | AX-11436952 | rs32060 | 5 | 156067682 | 0.6838 | 8.33E-05 |
| 163 | AX-11432475 | rs305191 | 2 | 36252564 | 0.7453 | 8.42E-05 |
| 164 | AX-11308376 | rs17126072 | 14 | 53507684 | 0.5724 | 8.45E-05 |
| 165 | AX-11427010 | rs2919328 | 8 | 137498842 | 0.634 | 8.52E-05 |
| 166 | AX-11357768 | rs1986059 | 21 | 29362621 | 1.513 | 8.53E-05 |
| 167 | AX-11277295 | rs1580930 | 5 | 8812449 | 0.7706 | 8.54E-05 |
| 168 | AX-11252970 | rs13407070 | 2 | 161426931 | 1.336 | 8.55E-05 |
| 169 | AX-11507309 | rs447938 | 5 | 155986366 | 0.6801 | 8.61E-05 |
| 170 | AX-11650909 | rs7866906 | 9 | 110314946 | 1.349 | 8.68E-05 |
| 171 | AX-11702406 | rs9787473 | 10 | 117864680 | 0.7692 | 8.73E-05 |
| 172 | AX-11338170 | rs17746658 | 10 | 84571269 | 1.503 | 8.74E-05 |
| 173 | AX-11614030 | rs7200499 | 16 | 8154645 | 1.331 | 8.77E-05 |
| 174 | AX-11652858 | rs7904348 | 10 | 35068814 | 1.311 | 8.81E-05 |
| 175 | AX-11231026 | rs12898967 | 15 | 59469098 | 0.7689 | 8.81E-05 |
| 176 | AX-11455474 | rs34934281 | 12 | 124330311 | 0.6443 | 8.86E-05 |
| 177 | AX-11643287 | rs7754329 | 6 | 112526404 | 1.495 | 8.92E-05 |
| 178 | AX-11424350 | rs28836770 | 3 | 190977465 | 1.29 | 8.99E-05 |
| 179 | AX-11164202 | rs11635454 | 15 | 98456576 | 1.294 | 9.05E-05 |
| 180 | AX-11156410 | rs11395174 | 7 | 68198342 | 1.421 | 9.06E-05 |
| 181 | AX-11182733 | rs11934382 | 4 | 149964525 | 1.29 | 9.17E-05 |
| 182 | AX-11568560 | rs6501072 | 16 | 8139350 | 1.33 | 9.17E-05 |
| 183 | AX-11205195 | rs12426074 | 12 | 26643310 | 1.345 | 9.24E-05 |
| 184 | AX-11332995 | rs17647019 | 7 | 17481089 | 0.507 | 9.31E-05 |
| 185 | AX-11115828 | rs10519956 | 15 | 35061841 | 0.7574 | 9.39E-05 |
| 186 | AX-11504364 | rs4406722 | 10 | 19901013 | 1.405 | 9.41E-05 |
| 187 | AX-11607232 | rs710705 | 12 | 70366573 | 0.6948 | 9.57E-05 |
| 188 | AX-11567795 | rs6490917 | 13 | 24997257 | 0.7651 | 9.59E-05 |
| 189 | AX-11648903 | rs7836091 | 8 | 135388625 | 1.284 | 9.61E-05 |
| 190 | AX-11098701 | rs10191480 | 2 | 45281360 | 0.7786 | 9.65E-05 |
| 191 | AX-11655278 | rs7944397 | 11 | 34455309 | 0.6729 | 9.66E-05 |
| 192 | AX-11211875 | rs12518444 | 5 | 81915148 | 1.519 | 9.72E-05 |
| 193 | AX-11102396 | rs1025438 | 11 | 8905461 | 0.6275 | 9.74E-05 |
| 194 | AX-11278494 | rs1605864 | 15 | 81420918 | 1.444 | 9.75E-05 |
| 195 | AX-11676990 | rs927240 | 20 | 57349312 | 1.287 | 9.77E-05 |
| 196 | AX-11504827 | rs4417246 | 11 | 18212384 | 1.294 | 9.94E-05 |
| 197 | AX-11616728 | rs7239484 | 18 | 55443712 | 1.343 | 0.0001007 |
| 198 | AX-11352184 | rs1911169 | 1 | 119085449 | 1.723 | 0.0001008 |
| 199 | AX-11314371 | rs17212065 | 14 | 25695907 | 1.433 | 0.0001011 |
| 200 | AX-11528541 | rs4816779 | 21 | 20141055 | 0.7806 | 0.0001016 |

| **B vs. CD** | | | | | | |
| --- | --- | --- | --- | --- | --- | --- |
| RANK | SNP | RS ID | CHR | BP | OR | P |
| 1 | AX-11557827 | rs6068583 | 20 | 52176105 | 1.554 | 3.38E-07 |
| 2 | AX-11655269 | rs7944251 | 11 | 91996431 | 0.6555 | 3.97E-07 |
| 3 | AX-11460370 | rs35226739 | 3 | 7470143 | 2.647 | 8.68E-07 |
| 4 | AX-11448821 | rs34548439 | 5 | 38831131 | 1.411 | 8.79E-07 |
| 5 | AX-11173852 | rs11766140 | 7 | 24519656 | 1.366 | 1.45E-06 |
| 6 | AX-11240830 | rs13129679 | 4 | 2576102 | 2.721 | 1.64E-06 |
| 7 | AX-11237561 | rs13055076 | 22 | 30069207 | 1.455 | 1.88E-06 |
| 8 | AX-11367824 | rs2110007 | 7 | 21193341 | 0.5075 | 2.97E-06 |
| 9 | AX-11668502 | rs829883 | 12 | 98879606 | 0.7281 | 3.68E-06 |
| 10 | AX-11477473 | rs3743102 | 15 | 83427394 | 1.452 | 5.37E-06 |
| 11 | AX-11130316 | rs10898160 | 11 | 83558374 | 1.351 | 5.87E-06 |
| 12 | AX-11368883 | rs2125062 | 10 | 97980721 | 1.335 | 6.09E-06 |
| 13 | AX-11209352 | rs12482500 | 21 | 43353610 | 1.365 | 6.42E-06 |
| 14 | AX-11438728 | rs338241 | 1 | 58934155 | 1.813 | 6.43E-06 |
| 15 | AX-11099787 | rs10208443 | 2 | 72329603 | 1.348 | 6.83E-06 |
| 16 | AX-11334750 | rs17679617 | 16 | 83308001 | 1.443 | 7.33E-06 |
| 17 | AX-11624436 | rs7425956 | 2 | 233945565 | 0.7421 | 7.38E-06 |
| 18 | AX-11415595 | rs2837983 | 21 | 42616757 | 0.6904 | 7.58E-06 |
| 19 | AX-11313235 | rs17190422 | 2 | 56856260 | 2.073 | 9.05E-06 |
| 20 | AX-11098423 | rs1018697 | 10 | 104549224 | 0.7391 | 9.08E-06 |
| 21 | AX-11555574 | rs6022591 | 20 | 52172614 | 1.385 | 9.20E-06 |
| 22 | AX-11155165 | rs1131510 | 10 | 35299085 | 1.748 | 9.48E-06 |
| 23 | AX-11433889 | rs3107431 | 4 | 107735838 | 1.451 | 9.63E-06 |
| 24 | AX-11687703 | rs9450814 | 6 | 88554867 | 1.937 | 9.79E-06 |
| 25 | AX-11376629 | rs2227714 | 7 | 100781909 | 0.3966 | 9.89E-06 |
| 26 | AX-11640496 | rs7712436 | 5 | 31485230 | 2.203 | 1.00E-05 |
| 27 | AX-11706516 | rs9857702 | 3 | 11166302 | 1.347 | 1.00E-05 |
| 28 | AX-11560357 | rs6125213 | 20 | 46666287 | 1.841 | 1.01E-05 |
| 29 | AX-11270137 | rs1507675 | 2 | 123778205 | 1.436 | 1.02E-05 |
| 30 | AX-11191221 | rs12120559 | 1 | 119844431 | 1.324 | 1.05E-05 |
| 31 | AX-11438438 | rs33409 | 5 | 150038265 | 0.7506 | 1.09E-05 |
| 32 | AX-11456145 | N/A | 8 | 18148918 | 0.7283 | 1.21E-05 |
| 33 | AX-11465883 | rs35551835 | 1 | 187942253 | 1.654 | 1.27E-05 |
| 34 | AX-11261345 | rs1415123 | 13 | 21541027 | 1.328 | 1.31E-05 |
| 35 | AX-11265096 | rs1454001 | 11 | 21262591 | 1.34 | 1.35E-05 |
| 36 | AX-11419829 | rs2857638 | 22 | 30053118 | 1.334 | 1.40E-05 |
| 37 | AX-11378216 | rs2245194 | 22 | 30048182 | 1.333 | 1.43E-05 |
| 38 | AX-11527894 | rs4809962 | 20 | 52931148 | 0.7437 | 1.58E-05 |
| 39 | AX-11134865 | rs10964101 | 9 | 1943156 | 1.507 | 1.63E-05 |
| 40 | AX-11137857 | rs11012183 | 10 | 20892753 | 0.3363 | 1.64E-05 |
| 41 | AX-11455993 | rs34964149 | 9 | 84269234 | 1.378 | 1.70E-05 |
| 42 | AX-11121453 | rs10783318 | 12 | 39070576 | 2.232 | 1.74E-05 |
| 43 | AX-11443749 | rs34250521 | 15 | 59507953 | 0.7081 | 1.76E-05 |
| 44 | AX-11352136 | rs1910243 | 12 | 38951794 | 2.286 | 1.84E-05 |
| 45 | AX-11195248 | rs1219742 | 10 | 125525042 | 1.432 | 1.86E-05 |
| 46 | AX-11615025 | rs7215468 | 17 | 37189723 | 0.7515 | 1.88E-05 |
| 47 | AX-11203107 | rs12378124 | 9 | 9668869 | 1.525 | 1.90E-05 |
| 48 | AX-11470941 | rs35851221 | 16 | 8882670 | 1.953 | 2.12E-05 |
| 49 | AX-11702579 | rs979006 | 4 | 54452878 | 1.458 | 2.14E-05 |
| 50 | AX-11689215 | rs947937 | 11 | 91991008 | 1.314 | 2.14E-05 |
| 51 | AX-11402806 | rs2641479 | 12 | 74958839 | 1.353 | 2.14E-05 |
| 52 | AX-11555382 | rs6018856 | 20 | 46636528 | 1.751 | 2.15E-05 |
| 53 | AX-11561662 | rs622199 | 3 | 73305297 | 1.901 | 2.15E-05 |
| 54 | AX-11478477 | rs3756654 | 5 | 157165246 | 1.326 | 2.17E-05 |
| 55 | AX-11219822 | rs12637835 | 3 | 11116366 | 0.6662 | 2.23E-05 |
| 56 | AX-11389076 | rs2391611 | 13 | 108670546 | 0.7095 | 2.27E-05 |
| 57 | AX-11402628 | rs2637513 | 5 | 124783400 | 1.746 | 2.52E-05 |
| 58 | AX-11307785 | rs17118810 | 14 | 47972984 | 2.085 | 2.61E-05 |
| 59 | AX-11325414 | rs1746789 | 9 | 9751064 | 1.641 | 2.63E-05 |
| 60 | AX-11274093 | rs1546308 | 12 | 21176135 | 1.612 | 2.68E-05 |
| 61 | AX-11601902 | rs7020782 | 9 | 119106881 | 1.326 | 2.71E-05 |
| 62 | AX-11697834 | rs9614158 | 22 | 30597880 | 1.323 | 2.74E-05 |
| 63 | AX-11140829 | rs11058824 | 12 | 127296840 | 0.5104 | 2.79E-05 |
| 64 | AX-11246010 | rs13244061 | 7 | 137784687 | 0.759 | 2.81E-05 |
| 65 | AX-11595945 | rs6940879 | 6 | 112492645 | 1.651 | 2.83E-05 |
| 66 | AX-11096864 | rs1016268 | 12 | 130001810 | 0.76 | 2.90E-05 |
| 67 | AX-11699745 | rs9660091 | 1 | 164732727 | 1.31 | 2.91E-05 |
| 68 | AX-11535636 | rs4912239 | 1 | 56878569 | 2.348 | 3.01E-05 |
| 69 | AX-11668705 | rs833664 | 3 | 62556868 | 1.838 | 3.07E-05 |
| 70 | AX-11571179 | rs6548744 | 3 | 81082571 | 1.373 | 3.13E-05 |
| 71 | AX-11285376 | rs16870061 | 5 | 2578112 | 1.347 | 3.14E-05 |
| 72 | AX-11619293 | rs7295114 | 12 | 13593729 | 0.7123 | 3.36E-05 |
| 73 | AX-11673730 | rs905344 | 12 | 38937391 | 2.183 | 3.38E-05 |
| 74 | AX-11261815 | rs1420413 | 12 | 98199400 | 0.7559 | 3.41E-05 |
| 75 | AX-11434638 | rs3118230 | 16 | 68709359 | 1.444 | 3.42E-05 |
| 76 | AX-11483437 | rs3822844 | 6 | 160421012 | 1.324 | 3.47E-05 |
| 77 | AX-11308890 | rs17131469 | 1 | 91961322 | 2.327 | 3.57E-05 |
| 78 | AX-11653309 | rs7911684 | 10 | 97971928 | 0.7665 | 3.67E-05 |
| 79 | AX-11387064 | rs2360087 | 16 | 8157001 | 1.332 | 3.72E-05 |
| 80 | AX-11110933 | rs10494114 | 1 | 110687962 | 1.579 | 3.78E-05 |
| 81 | AX-11685929 | rs9400697 | 6 | 114484080 | 0.6592 | 3.92E-05 |
| 82 | AX-11373171 | rs2182729 | 9 | 18917347 | 1.31 | 3.94E-05 |
| 83 | AX-11625350 | rs7459717 | 8 | 107511717 | 0.7628 | 3.95E-05 |
| 84 | AX-11707456 | rs9871676 | 3 | 114072839 | 0.7651 | 3.99E-05 |
| 85 | AX-11461801 | rs35312459 | 4 | 56721403 | 1.561 | 4.01E-05 |
| 86 | AX-11356778 | rs1972065 | 12 | 37898034 | 2.12 | 4.03E-05 |
| 87 | AX-11603569 | rs7045124 | 9 | 2661766 | 0.7173 | 4.08E-05 |
| 88 | AX-11234681 | rs12980083 | 19 | 22968938 | 1.891 | 4.20E-05 |
| 89 | AX-11489946 | rs4073374 | 3 | 123055019 | 1.44 | 4.28E-05 |
| 90 | AX-11151251 | rs11213669 | 11 | 110820365 | 1.653 | 4.31E-05 |
| 91 | AX-11154789 | N/A | 20 | 54088377 | 1.614 | 4.36E-05 |
| 92 | AX-11209979 | rs12492298 | 3 | 65720358 | 1.871 | 4.36E-05 |
| 93 | AX-11631869 | rs7588841 | 2 | 177687497 | 1.41 | 4.40E-05 |
| 94 | AX-11110145 | rs10489877 | 1 | 192570150 | 1.345 | 4.45E-05 |
| 95 | AX-11304540 | rs17083254 | 4 | 63887305 | 1.522 | 4.48E-05 |
| 96 | AX-11175604 | rs11787299 | 8 | 18141181 | 0.7536 | 4.48E-05 |
| 97 | AX-11479296 | rs3769498 | 2 | 106488411 | 0.7312 | 4.52E-05 |
| 98 | AX-11346633 | rs1843640 | 12 | 39137722 | 1.644 | 4.54E-05 |
| 99 | AX-11469116 | rs35742853 | 12 | 98160685 | 1.729 | 4.59E-05 |
| 100 | AX-11181459 | rs11907654 | 20 | 30512051 | 1.508 | 4.64E-05 |
| 101 | AX-11088818 | rs10037634 | 5 | 62385531 | 1.301 | 4.68E-05 |
| 102 | AX-11664456 | rs8082768 | 18 | 57641728 | 0.6871 | 4.72E-05 |
| 103 | AX-11305657 | rs17095503 | 1 | 74984208 | 2.417 | 4.77E-05 |
| 104 | AX-11648821 | rs7834906 | 8 | 18175677 | 0.7345 | 4.85E-05 |
| 105 | AX-11421190 | rs28639642 | 4 | 71376971 | 0.7185 | 4.87E-05 |
| 106 | AX-11590140 | rs6854895 | 4 | 78851940 | 0.741 | 4.88E-05 |
| 107 | AX-11323386 | rs17416859 | 5 | 121321055 | 1.868 | 4.90E-05 |
| 108 | AX-11524048 | rs75452819 | 12 | 128165677 | 1.538 | 4.90E-05 |
| 109 | AX-11240162 | rs13114658 | 4 | 1052009 | 1.295 | 4.91E-05 |
| 110 | AX-11402714 | rs2639270 | 3 | 81089293 | 1.362 | 4.93E-05 |
| 111 | AX-11577846 | rs6678122 | 1 | 62301986 | 1.637 | 4.93E-05 |
| 112 | AX-11366685 | rs2088823 | 1 | 19774133 | 1.309 | 5.04E-05 |
| 113 | AX-11696735 | rs959324 | 15 | 48670904 | 1.442 | 5.05E-05 |
| 114 | AX-11659506 | rs8007495 | 14 | 28933330 | 1.341 | 5.14E-05 |
| 115 | AX-11374298 | rs219551 | 2 | 21540711 | 1.416 | 5.19E-05 |
| 116 | AX-11408807 | rs274713 | 5 | 6723560 | 0.7495 | 5.37E-05 |
| 117 | AX-11493878 | rs4130464 | 2 | 228342678 | 1.732 | 5.48E-05 |
| 118 | AX-11293109 | rs16955379 | 16 | 81489373 | 2.434 | 5.50E-05 |
| 119 | AX-11603767 | rs7047972 | 9 | 1945517 | 1.584 | 5.67E-05 |
| 120 | AX-11142207 | rs1107573 | 11 | 34456208 | 0.4727 | 5.73E-05 |
| 121 | AX-11123045 | rs10803233 | 1 | 236189722 | 1.298 | 5.85E-05 |
| 122 | AX-11483928 | rs3829273 | 11 | 9048668 | 0.6755 | 5.86E-05 |
| 123 | AX-11451102 | rs34680385 | 8 | 4761302 | 0.4534 | 5.88E-05 |
| 124 | AX-11259342 | rs1393109 | 5 | 38812605 | 1.321 | 5.89E-05 |
| 125 | AX-11168682 | rs11697210 | 20 | 13873821 | 1.3 | 5.93E-05 |
| 126 | AX-11286482 | rs16882479 | 8 | 34443390 | 1.585 | 5.94E-05 |
| 127 | AX-11109103 | rs10483612 | 14 | 53034269 | 1.387 | 5.95E-05 |
| 128 | AX-11243561 | rs13188627 | 5 | 2940440 | 0.7572 | 6.05E-05 |
| 129 | AX-11483301 | rs3821212 | 2 | 58443598 | 0.6577 | 6.05E-05 |
| 130 | AX-11405873 | rs2702934 | 8 | 6707885 | 1.408 | 6.14E-05 |
| 131 | AX-11088300 | rs10028954 | 4 | 70466280 | 1.292 | 6.19E-05 |
| 132 | AX-11110033 | rs10489270 | 1 | 173157764 | 1.462 | 6.36E-05 |
| 133 | AX-11712984 | rs9963872 | 18 | 64076587 | 1.33 | 6.39E-05 |
| 134 | AX-11155261 | N/A | 1 | 74227803 | 1.585 | 6.40E-05 |
| 135 | AX-11642476 | rs7742915 | 6 | 38147745 | 1.322 | 6.43E-05 |
| 136 | AX-11374526 | rs2198895 | 19 | 31369194 | 1.38 | 6.54E-05 |
| 137 | AX-11638671 | rs7685184 | 4 | 68830891 | 1.723 | 6.65E-05 |
| 138 | AX-11329503 | rs17574363 | 1 | 74122299 | 1.528 | 6.70E-05 |
| 139 | AX-11379089 | rs2253894 | 21 | 42710566 | 1.388 | 6.95E-05 |
| 140 | AX-11559669 | rs6107151 | 20 | 26158945 | 1.437 | 6.99E-05 |
| 141 | AX-11311782 | rs17166395 | 7 | 12820897 | 1.786 | 7.02E-05 |
| 142 | AX-11332730 | rs17642361 | 8 | 18147962 | 0.7504 | 7.04E-05 |
| 143 | AX-11283359 | rs16848124 | 2 | 213123460 | 0.7445 | 7.12E-05 |
| 144 | AX-11569436 | rs6517312 | 21 | 37277069 | 1.896 | 7.16E-05 |
| 145 | AX-11657204 | rs7973380 | 12 | 39569858 | 2.24 | 7.18E-05 |
| 146 | AX-11416982 | rs28437505 | 4 | 84252514 | 1.918 | 7.21E-05 |
| 147 | AX-11300153 | rs17038353 | 1 | 119046507 | 1.966 | 7.23E-05 |
| 148 | AX-11289618 | rs16917259 | 11 | 91994044 | 0.681 | 7.45E-05 |
| 149 | AX-11317075 | rs172705 | 3 | 4345033 | 1.615 | 7.48E-05 |
| 150 | AX-11331956 | rs17626900 | 2 | 195841000 | 0.502 | 7.57E-05 |
| 151 | AX-11480707 | rs3787970 | 21 | 43651556 | 0.7731 | 7.58E-05 |
| 152 | AX-11711772 | rs9944240 | 15 | 47504936 | 1.292 | 7.65E-05 |
| 153 | AX-11478264 | rs3753366 | 1 | 68195133 | 1.374 | 7.80E-05 |
| 154 | AX-11337675 | rs17737005 | 12 | 81795085 | 2.003 | 7.81E-05 |
| 155 | AX-11563839 | rs6435236 | 2 | 205253405 | 0.7463 | 7.83E-05 |
| 156 | AX-11127411 | rs10859713 | 12 | 94907751 | 0.7716 | 7.86E-05 |
| 157 | AX-11524710 | rs4769 | 11 | 8968885 | 0.6521 | 7.88E-05 |
| 158 | AX-11437070 | rs321198 | 7 | 137029838 | 1.287 | 7.93E-05 |
| 159 | AX-11112099 | rs10500593 | 11 | 4093962 | 2.019 | 8.07E-05 |
| 160 | AX-11180935 | rs11896830 | 2 | 208237126 | 1.429 | 8.12E-05 |
| 161 | AX-11117874 | rs10739534 | 9 | 122071105 | 1.287 | 8.23E-05 |
| 162 | AX-11436952 | rs32060 | 5 | 156067682 | 0.6838 | 8.33E-05 |
| 163 | AX-11432475 | rs305191 | 2 | 36252564 | 0.7453 | 8.42E-05 |
| 164 | AX-11308376 | rs17126072 | 14 | 53507684 | 0.5724 | 8.45E-05 |
| 165 | AX-11427010 | rs2919328 | 8 | 137498842 | 0.634 | 8.52E-05 |
| 166 | AX-11357768 | rs1986059 | 21 | 29362621 | 1.513 | 8.53E-05 |
| 167 | AX-11277295 | rs1580930 | 5 | 8812449 | 0.7706 | 8.54E-05 |
| 168 | AX-11252970 | rs13407070 | 2 | 161426931 | 1.336 | 8.55E-05 |
| 169 | AX-11507309 | rs447938 | 5 | 155986366 | 0.6801 | 8.61E-05 |
| 170 | AX-11650909 | rs7866906 | 9 | 110314946 | 1.349 | 8.68E-05 |
| 171 | AX-11702406 | rs9787473 | 10 | 117864680 | 0.7692 | 8.73E-05 |
| 172 | AX-11338170 | rs17746658 | 10 | 84571269 | 1.503 | 8.74E-05 |
| 173 | AX-11614030 | rs7200499 | 16 | 8154645 | 1.331 | 8.77E-05 |
| 174 | AX-11652858 | rs7904348 | 10 | 35068814 | 1.311 | 8.81E-05 |
| 175 | AX-11231026 | rs12898967 | 15 | 59469098 | 0.7689 | 8.81E-05 |
| 176 | AX-11455474 | rs34934281 | 12 | 124330311 | 0.6443 | 8.86E-05 |
| 177 | AX-11643287 | rs7754329 | 6 | 112526404 | 1.495 | 8.92E-05 |
| 178 | AX-11424350 | rs28836770 | 3 | 190977465 | 1.29 | 8.99E-05 |
| 179 | AX-11164202 | rs11635454 | 15 | 98456576 | 1.294 | 9.05E-05 |
| 180 | AX-11156410 | rs11395174 | 7 | 68198342 | 1.421 | 9.06E-05 |
| 181 | AX-11182733 | rs11934382 | 4 | 149964525 | 1.29 | 9.17E-05 |
| 182 | AX-11568560 | rs6501072 | 16 | 8139350 | 1.33 | 9.17E-05 |
| 183 | AX-11205195 | rs12426074 | 12 | 26643310 | 1.345 | 9.24E-05 |
| 184 | AX-11332995 | rs17647019 | 7 | 17481089 | 0.507 | 9.31E-05 |
| 185 | AX-11115828 | rs10519956 | 15 | 35061841 | 0.7574 | 9.39E-05 |
| 186 | AX-11504364 | rs4406722 | 10 | 19901013 | 1.405 | 9.41E-05 |
| 187 | AX-11607232 | rs710705 | 12 | 70366573 | 0.6948 | 9.57E-05 |
| 188 | AX-11567795 | rs6490917 | 13 | 24997257 | 0.7651 | 9.59E-05 |
| 189 | AX-11648903 | rs7836091 | 8 | 135388625 | 1.284 | 9.61E-05 |
| 190 | AX-11098701 | rs10191480 | 2 | 45281360 | 0.7786 | 9.65E-05 |
| 191 | AX-11655278 | rs7944397 | 11 | 34455309 | 0.6729 | 9.66E-05 |
| 192 | AX-11211875 | rs12518444 | 5 | 81915148 | 1.519 | 9.72E-05 |
| 193 | AX-11102396 | rs1025438 | 11 | 8905461 | 0.6275 | 9.74E-05 |
| 194 | AX-11278494 | rs1605864 | 15 | 81420918 | 1.444 | 9.75E-05 |
| 195 | AX-11676990 | rs927240 | 20 | 57349312 | 1.287 | 9.77E-05 |
| 196 | AX-11504827 | rs4417246 | 11 | 18212384 | 1.294 | 9.94E-05 |
| 197 | AX-11616728 | rs7239484 | 18 | 55443712 | 1.343 | 0.0001007 |
| 198 | AX-11352184 | rs1911169 | 1 | 119085449 | 1.723 | 0.0001008 |
| 199 | AX-11314371 | rs17212065 | 14 | 25695907 | 1.433 | 0.0001011 |
| 200 | AX-11528541 | rs4816779 | 21 | 20141055 | 0.7806 | 0.0001016 |

| **C vs. D** | | | | | | |
| --- | --- | --- | --- | --- | --- | --- |
| RANK | SNP | RS ID | CHR | BP | OR | P |
| 1 | AX-11384835 | rs2323183 | 17 | 14307405 | 1.98 | 2.23E-08 |
| 2 | AX-11427227 | rs29234 | 6 | 29624112 | 2.219 | 2.45E-08 |
| 3 | AX-11239937 | rs13109974 | 4 | 43787479 | 1.669 | 2.56E-08 |
| 4 | AX-11434550 | rs3117286 | 6 | 29629774 | 2.484 | 6.15E-08 |
| 5 | AX-11397331 | rs2535248 | 6 | 29634927 | 2.483 | 6.23E-08 |
| 6 | AX-11397332 | rs2535250 | 6 | 29634417 | 2.479 | 6.51E-08 |
| 7 | AX-11430417 | N/A | 6 | 29630360 | 2.467 | 7.38E-08 |
| 8 | AX-11427420 | rs29272 | 6 | 29618366 | 2.173 | 1.13E-07 |
| 9 | AX-11597822 | rs6967514 | 7 | 47896158 | 1.408 | 1.81E-07 |
| 10 | AX-11195327 | rs12198456 | 6 | 120371988 | 2.909 | 1.82E-07 |
| 11 | AX-11669448 | rs847851 | 6 | 34904584 | 1.442 | 2.01E-07 |
| 12 | AX-11669447 | rs847848 | 6 | 34906168 | 1.536 | 2.09E-07 |
| 13 | AX-11493053 | rs41290575 | 5 | 150633108 | 1.806 | 2.45E-07 |
| 14 | AX-11713135 | rs9965938 | 18 | 70885038 | 1.72 | 2.83E-07 |
| 15 | AX-11151194 | rs11212737 | 11 | 108510682 | 0.7024 | 2.86E-07 |
| 16 | AX-11523829 | rs4757965 | 11 | 12914979 | 0.5099 | 3.13E-07 |
| 17 | AX-11681091 | rs932287 | 11 | 9044767 | 1.413 | 3.27E-07 |
| 18 | AX-11628652 | rs7542767 | 1 | 184313660 | 0.5778 | 3.39E-07 |
| 19 | AX-11109554 | rs10485879 | 7 | 77905130 | 0.6444 | 3.95E-07 |
| 20 | AX-11705096 | rs9836145 | 3 | 70251995 | 2.39 | 3.96E-07 |
| 21 | AX-11113230 | rs10506682 | 12 | 75366735 | 1.554 | 4.45E-07 |
| 22 | AX-11473642 | rs36012009 | 2 | 215440962 | 1.83 | 4.67E-07 |
| 23 | AX-11656628 | rs7965164 | 12 | 105052813 | 1.434 | 5.01E-07 |
| 24 | AX-11213657 | rs12542260 | 8 | 125502440 | 1.47 | 5.01E-07 |
| 25 | AX-11256613 | rs1364064 | 16 | 73462486 | 1.393 | 5.12E-07 |
| 26 | AX-11408828 | rs2747459 | 6 | 29657071 | 2.704 | 5.34E-07 |
| 27 | AX-11263415 | rs1436294 | 4 | 20168391 | 0.7156 | 5.91E-07 |
| 28 | AX-11191609 | rs12126082 | 1 | 216928519 | 1.573 | 8.74E-07 |
| 29 | AX-11570789 | rs6543990 | 2 | 36240074 | 0.7078 | 9.00E-07 |
| 30 | AX-11412459 | rs2821158 | 9 | 11664363 | 2.584 | 9.11E-07 |
| 31 | AX-11111213 | rs10495672 | 2 | 18494455 | 0.685 | 9.99E-07 |
| 32 | AX-11312897 | rs17184633 | 6 | 29672415 | 2.654 | 1.01E-06 |
| 33 | AX-11192346 | rs12136737 | 1 | 184451582 | 0.3749 | 1.05E-06 |
| 34 | AX-11526980 | rs4796354 | 17 | 6682884 | 1.389 | 1.07E-06 |
| 35 | AX-11544712 | rs553685 | 3 | 172089253 | 0.4443 | 1.19E-06 |
| 36 | AX-11645753 | rs7790642 | 7 | 148600310 | 1.383 | 1.19E-06 |
| 37 | AX-11334563 | rs17676109 | 12 | 63050251 | 0.4069 | 1.27E-06 |
| 38 | AX-11606138 | rs709082 | 3 | 191447446 | 1.382 | 1.45E-06 |
| 39 | AX-11098880 | rs10193935 | 2 | 42419829 | 1.532 | 1.46E-06 |
| 40 | AX-11517565 | rs4680748 | 3 | 28293360 | 0.7211 | 1.58E-06 |
| 41 | AX-11688427 | rs9465187 | 6 | 18684871 | 1.372 | 1.71E-06 |
| 42 | AX-11427232 | rs29235 | 6 | 29624078 | 2.163 | 1.74E-06 |
| 43 | AX-11233083 | rs12941828 | 17 | 6427079 | 1.4 | 1.74E-06 |
| 44 | AX-11668712 | rs833769 | 2 | 36228472 | 0.7309 | 1.77E-06 |
| 45 | AX-11408814 | rs2747420 | 6 | 29642434 | 2.159 | 1.91E-06 |
| 46 | AX-11447642 | rs34481134 | 2 | 22868209 | 2.199 | 1.92E-06 |
| 47 | AX-11271782 | rs1525293 | 7 | 70473005 | 1.37 | 1.93E-06 |
| 48 | AX-11257007 | rs1368141 | 3 | 116510192 | 1.867 | 1.93E-06 |
| 49 | AX-11321708 | rs17373662 | 16 | 73466538 | 1.367 | 1.94E-06 |
| 50 | AX-11157832 | rs1148737 | 21 | 14669931 | 1.369 | 1.96E-06 |
| 51 | AX-11327469 | rs17519683 | 11 | 83161160 | 1.396 | 2.02E-06 |
| 52 | AX-11557117 | rs6052563 | 20 | 4414604 | 1.945 | 2.03E-06 |
| 53 | AX-11454170 | rs34856929 | 5 | 147185679 | 0.3425 | 2.08E-06 |
| 54 | AX-11621755 | rs7328279 | 13 | 42047398 | 2.274 | 2.15E-06 |
| 55 | AX-11206664 | rs12444232 | 16 | 20321467 | 1.996 | 2.15E-06 |
| 56 | AX-11313214 | rs17189998 | 8 | 73803750 | 1.665 | 2.19E-06 |
| 57 | AX-11145745 | rs11129766 | 3 | 435143 | 2.797 | 2.31E-06 |
| 58 | AX-11456025 | rs34965331 | 2 | 88993291 | 1.746 | 2.35E-06 |
| 59 | AX-11429712 | rs2971100 | 7 | 9922243 | 1.911 | 2.40E-06 |
| 60 | AX-11506992 | rs4471510 | 12 | 75246981 | 1.388 | 2.52E-06 |
| 61 | AX-11265736 | rs1461596 | 4 | 91903467 | 1.881 | 2.54E-06 |
| 62 | AX-11172700 | rs11750868 | 5 | 159264675 | 1.466 | 2.60E-06 |
| 63 | AX-11565538 | rs6460638 | 7 | 70546596 | 1.734 | 2.63E-06 |
| 64 | AX-11687762 | rs9451766 | 6 | 65204953 | 2.795 | 2.68E-06 |
| 65 | AX-11654988 | rs7939569 | 11 | 84377621 | 0.6504 | 2.70E-06 |
| 66 | AX-11654244 | rs7927484 | 11 | 123138960 | 1.468 | 2.86E-06 |
| 67 | AX-11341156 | rs17804008 | 17 | 6433659 | 1.371 | 2.91E-06 |
| 68 | AX-11586366 | rs6800659 | 3 | 28270669 | 0.722 | 3.03E-06 |
| 69 | AX-11501869 | rs4342066 | 3 | 28575932 | 0.7102 | 3.14E-06 |
| 70 | AX-11350602 | rs1889642 | 13 | 80406220 | 1.361 | 3.15E-06 |
| 71 | AX-11281933 | rs16831235 | 2 | 135745129 | 1.459 | 3.24E-06 |
| 72 | AX-11138921 | rs11028546 | 11 | 25291015 | 2.881 | 3.40E-06 |
| 73 | AX-11323242 | rs17413224 | 2 | 222038917 | 0.5658 | 3.44E-06 |
| 74 | AX-11517223 | rs4676482 | 3 | 39237190 | 0.66 | 3.49E-06 |
| 75 | AX-11397327 | N/A | 6 | 29652163 | 2.556 | 3.49E-06 |
| 76 | AX-11397333 | rs2535255 | 6 | 29631690 | 2.556 | 3.49E-06 |
| 77 | AX-11397334 | rs2535256 | 6 | 29631628 | 2.556 | 3.49E-06 |
| 78 | AX-11408824 | N/A | 6 | 29654898 | 2.556 | 3.49E-06 |
| 79 | AX-11408831 | rs2747466 | 6 | 29658569 | 2.556 | 3.49E-06 |
| 80 | AX-11419853 | rs2857768 | 6 | 29634511 | 2.556 | 3.49E-06 |
| 81 | AX-11419855 | rs2857783 | 6 | 29631588 | 2.556 | 3.49E-06 |
| 82 | AX-11419856 | rs2857784 | 6 | 29632350 | 2.556 | 3.49E-06 |
| 83 | AX-11480571 | rs378596 | 6 | 29643287 | 2.556 | 3.49E-06 |
| 84 | AX-11496791 | rs416560 | 6 | 29648785 | 2.556 | 3.49E-06 |
| 85 | AX-11315256 | rs17230787 | 10 | 33708150 | 0.6405 | 3.52E-06 |
| 86 | AX-11213706 | rs12542896 | 8 | 108284910 | 0.5494 | 3.53E-06 |
| 87 | AX-11434554 | rs3117298 | 6 | 29653843 | 2.554 | 3.56E-06 |
| 88 | AX-11655815 | rs7953792 | 12 | 97801072 | 1.474 | 3.59E-06 |
| 89 | AX-11435080 | rs3129040 | 6 | 29657033 | 2.55 | 3.64E-06 |
| 90 | AX-11422813 | rs28719689 | 8 | 1270670 | 1.584 | 3.74E-06 |
| 91 | AX-11138524 | rs11022523 | 11 | 12894257 | 0.7001 | 3.76E-06 |
| 92 | AX-11486014 | rs387603 | 6 | 29645578 | 2.545 | 3.82E-06 |
| 93 | AX-11526721 | rs4793259 | 17 | 69387909 | 0.591 | 3.93E-06 |
| 94 | AX-11365867 | rs2075466 | 16 | 4872970 | 1.386 | 3.94E-06 |
| 95 | AX-11153186 | rs1124356 | 16 | 78929313 | 1.354 | 3.95E-06 |
| 96 | AX-11209375 | rs12482963 | 21 | 23972323 | 1.393 | 3.96E-06 |
| 97 | AX-11309308 | rs17137294 | 16 | 4867908 | 1.628 | 4.02E-06 |
| 98 | AX-11113432 | rs10507824 | 13 | 74628682 | 2.265 | 4.11E-06 |
| 99 | AX-11486189 | rs38864 | 7 | 116818293 | 2.105 | 4.15E-06 |
| 100 | AX-11163538 | rs11627061 | 14 | 78580717 | 2.19 | 4.21E-06 |
| 101 | AX-11558688 | rs6082278 | 20 | 20987511 | 1.367 | 4.27E-06 |
| 102 | AX-11231785 | rs12913832 | 15 | 28365618 | 1.397 | 4.35E-06 |
| 103 | AX-11475110 | rs36098945 | 4 | 133349983 | 0.4412 | 4.36E-06 |
| 104 | AX-11427598 | rs2930353 | 8 | 3717468 | 1.363 | 4.51E-06 |
| 105 | AX-11624387 | rs7424369 | 2 | 221799497 | 2.638 | 4.59E-06 |
| 106 | AX-11382338 | rs229100 | 21 | 28223092 | 0.649 | 4.67E-06 |
| 107 | AX-11397326 | rs2535232 | 6 | 29656265 | 2.517 | 4.78E-06 |
| 108 | AX-11087310 | rs10012525 | 4 | 16912717 | 1.836 | 4.81E-06 |
| 109 | AX-11588210 | rs6826653 | 4 | 20127041 | 1.354 | 4.89E-06 |
| 110 | AX-11116524 | rs1056692 | 12 | 53687387 | 1.37 | 4.94E-06 |
| 111 | AX-11592712 | rs6895024 | 5 | 124529484 | 1.348 | 4.98E-06 |
| 112 | AX-11208196 | rs12466960 | 2 | 28567626 | 0.7375 | 4.98E-06 |
| 113 | AX-11520308 | rs4714902 | 6 | 46044237 | 1.363 | 5.07E-06 |
| 114 | AX-11459375 | rs35166252 | 20 | 37695601 | 1.369 | 5.12E-06 |
| 115 | AX-11643623 | rs7759216 | 6 | 106588614 | 0.7365 | 5.15E-06 |
| 116 | AX-11408648 | rs274437 | 6 | 104776654 | 0.6865 | 5.23E-06 |
| 117 | AX-11131135 | rs10910660 | 1 | 233242006 | 0.7005 | 5.25E-06 |
| 118 | AX-11498113 | rs4244207 | 5 | 90425042 | 1.484 | 5.50E-06 |
| 119 | AX-11547349 | rs579113 | 20 | 48075912 | 1.347 | 5.61E-06 |
| 120 | AX-11437598 | rs324126 | 19 | 52886137 | 1.36 | 5.69E-06 |
| 121 | AX-11620530 | rs7311952 | 12 | 62953773 | 1.37 | 5.69E-06 |
| 122 | AX-11351819 | rs1905108 | 8 | 49545585 | 1.358 | 5.89E-06 |
| 123 | AX-11622912 | rs735129 | 5 | 65540329 | 1.598 | 5.90E-06 |
| 124 | AX-11279873 | rs1639906 | 7 | 2219966 | 1.341 | 6.12E-06 |
| 125 | AX-11489822 | rs40719 | 5 | 9171202 | 1.339 | 6.21E-06 |
| 126 | AX-11495065 | rs41364745 | 8 | 132433065 | 2.109 | 6.34E-06 |
| 127 | AX-11692235 | rs9528056 | 13 | 60614522 | 0.3824 | 6.39E-06 |
| 128 | AX-11359120 | rs2000796 | 11 | 123136552 | 1.467 | 6.41E-06 |
| 129 | AX-11402864 | rs2642611 | 10 | 87093001 | 1.791 | 6.43E-06 |
| 130 | AX-11304627 | rs17084111 | 6 | 122336568 | 2.15 | 6.48E-06 |
| 131 | AX-11427050 | rs2920003 | 18 | 28792998 | 1.719 | 6.72E-06 |
| 132 | AX-11637456 | rs7666738 | 4 | 98968077 | 0.7215 | 6.82E-06 |
| 133 | AX-11377179 | rs2235769 | 20 | 37779569 | 1.411 | 6.87E-06 |
| 134 | AX-11625473 | rs7463659 | 8 | 135432140 | 0.744 | 6.92E-06 |
| 135 | AX-11394683 | rs2490361 | 1 | 237326974 | 1.36 | 7.05E-06 |
| 136 | AX-11406573 | rs2712475 | 3 | 106208882 | 0.6563 | 7.15E-06 |
| 137 | AX-11558687 | rs6082276 | 20 | 20985138 | 1.397 | 7.15E-06 |
| 138 | AX-11408821 | rs2747444 | 6 | 29653292 | 2.576 | 7.35E-06 |
| 139 | AX-11415544 | rs2837862 | 21 | 42260055 | 1.589 | 7.39E-06 |
| 140 | AX-11124326 | rs10817971 | 9 | 119766782 | 0.7107 | 7.39E-06 |
| 141 | AX-11506317 | rs4454940 | 15 | 88272469 | 0.7362 | 7.42E-06 |
| 142 | AX-11177258 | rs11818462 | 10 | 107077272 | 0.5969 | 7.52E-06 |
| 143 | AX-11538172 | rs4948237 | 10 | 59874367 | 0.4374 | 7.55E-06 |
| 144 | AX-11533645 | rs4887200 | 15 | 88311039 | 1.803 | 7.60E-06 |
| 145 | AX-11512141 | rs4574657 | 6 | 124319710 | 1.855 | 7.62E-06 |
| 146 | AX-11562070 | rs629236 | 11 | 35524561 | 1.376 | 7.67E-06 |
| 147 | AX-11434556 | rs3117300 | 6 | 29654624 | 2.526 | 7.81E-06 |
| 148 | AX-11168490 | rs11694459 | 2 | 46423447 | 0.4514 | 7.85E-06 |
| 149 | AX-11709890 | rs9913564 | 17 | 9001241 | 1.368 | 7.92E-06 |
| 150 | AX-11194771 | rs12190843 | 6 | 102526507 | 1.656 | 7.97E-06 |
| 151 | AX-11502046 | rs4346635 | 4 | 81486272 | 1.474 | 7.98E-06 |
| 152 | AX-11537621 | rs49413 | 3 | 59757484 | 1.405 | 8.04E-06 |
| 153 | AX-11503744 | rs4391125 | 5 | 65858018 | 1.476 | 8.07E-06 |
| 154 | AX-11370013 | rs2142109 | 21 | 40301956 | 1.481 | 8.10E-06 |
| 155 | AX-11658341 | rs7989397 | 13 | 104311156 | 1.957 | 8.12E-06 |
| 156 | AX-11668500 | rs829880 | 12 | 98845331 | 1.348 | 8.16E-06 |
| 157 | AX-11693647 | rs9545245 | 13 | 80445558 | 1.341 | 8.17E-06 |
| 158 | AX-11406977 | rs2718930 | 15 | 56543546 | 0.6896 | 8.29E-06 |
| 159 | AX-11260530 | rs1406084 | 2 | 154688728 | 1.432 | 8.29E-06 |
| 160 | AX-11199711 | rs12301672 | 12 | 75549401 | 1.395 | 8.33E-06 |
| 161 | AX-11658064 | rs7985095 | 13 | 108681130 | 0.7383 | 8.39E-06 |
| 162 | AX-11612691 | rs7182621 | 15 | 100424605 | 0.7352 | 8.49E-06 |
| 163 | AX-11344433 | rs179760 | 16 | 27441283 | 1.391 | 8.57E-06 |
| 164 | AX-11533662 | rs4887328 | 15 | 88349075 | 0.7154 | 8.64E-06 |
| 165 | AX-11452327 | rs34749153 | 9 | 119899453 | 1.368 | 8.65E-06 |
| 166 | AX-11388009 | rs237477 | 20 | 48057448 | 0.7362 | 8.74E-06 |
| 167 | AX-11523305 | rs4751006 | 10 | 128795523 | 0.5784 | 8.84E-06 |
| 168 | AX-11445781 | rs34368983 | 10 | 23160820 | 0.4082 | 8.88E-06 |
| 169 | AX-11170878 | rs11727186 | 4 | 20164834 | 1.412 | 8.91E-06 |
| 170 | AX-11509870 | rs45449196 | 3 | 8922458 | 2.28 | 9.06E-06 |
| 171 | AX-11320522 | rs17345786 | 3 | 101318318 | 0.6984 | 9.22E-06 |
| 172 | AX-11669320 | rs845868 | 6 | 998813 | 1.402 | 9.39E-06 |
| 173 | AX-11302148 | rs17059979 | 13 | 40038474 | 2.04 | 9.40E-06 |
| 174 | AX-11643411 | rs7756089 | 6 | 11241625 | 1.639 | 9.44E-06 |
| 175 | AX-11242744 | rs13171158 | 5 | 3171701 | 0.6837 | 9.51E-06 |
| 176 | AX-11098299 | rs10185229 | 2 | 77918355 | 1.395 | 9.59E-06 |
| 177 | AX-11123957 | rs10814587 | 9 | 3770175 | 0.6401 | 9.68E-06 |
| 178 | AX-11106857 | rs10439295 | 2 | 169569931 | 2.648 | 9.69E-06 |
| 179 | AX-11228584 | rs12830749 | 12 | 119216720 | 0.5104 | 9.72E-06 |
| 180 | AX-11419513 | rs28563040 | 4 | 4996573 | 1.343 | 9.81E-06 |
| 181 | AX-11125062 | rs10828093 | 10 | 20928039 | 0.6535 | 9.84E-06 |
| 182 | AX-11522725 | rs4743999 | 9 | 93417992 | 1.341 | 9.98E-06 |
| 183 | AX-11169106 | rs11704546 | 22 | 28204797 | 1.355 | 9.99E-06 |
| 184 | AX-11107510 | rs10456163 | 6 | 53574787 | 0.6979 | 1.01E-05 |
| 185 | AX-11288942 | rs16909124 | 12 | 13617949 | 1.916 | 1.01E-05 |
| 186 | AX-11142023 | rs11073746 | 15 | 88361491 | 0.7409 | 1.03E-05 |
| 187 | AX-11265845 | rs1462450 | 18 | 49204707 | 0.6483 | 1.03E-05 |
| 188 | AX-11394653 | rs248989 | 5 | 80436584 | 0.7501 | 1.05E-05 |
| 189 | AX-11281737 | rs16828452 | 3 | 157580410 | 1.542 | 1.06E-05 |
| 190 | AX-11669445 | rs847844 | 6 | 34920326 | 1.738 | 1.06E-05 |
| 191 | AX-11432147 | rs3025650 | 6 | 29542947 | 2.086 | 1.08E-05 |
| 192 | AX-11213035 | rs12533442 | 7 | 117597969 | 2.315 | 1.10E-05 |
| 193 | AX-11445320 | rs34341155 | 2 | 181788598 | 1.731 | 1.12E-05 |
| 194 | AX-11378037 | rs2243368 | 6 | 117045491 | 0.5425 | 1.14E-05 |
| 195 | AX-11196441 | rs12214473 | 6 | 102474325 | 1.509 | 1.16E-05 |
| 196 | AX-11666391 | rs811690 | 9 | 119404723 | 1.502 | 1.17E-05 |
| 197 | AX-11497040 | rs422001 | 20 | 15249445 | 1.369 | 1.18E-05 |
| 198 | AX-11530142 | rs4838964 | 1 | 113292658 | 1.329 | 1.18E-05 |
| 199 | AX-11331940 | rs17626544 | 6 | 18404450 | 0.6184 | 1.19E-05 |
| 200 | AX-11366798 | rs2091275 | 7 | 70514550 | 1.335 | 1.21E-05 |

The four different case and control groups were colorectal cancer cases **(A)**, advanced colorectal adenomas **(B)**, CORSA controls **(C)** and KORA controls **(D)**.

Abbreviations: RANK (SNP position according to assortment by P-value), SNP (Affymetrix SNP ID), RS ID (rs SNP ID), CHR (chromosome), BP (base position), OR, P (odds ratios and P-values based on the univariate logistic model).
